# Dynamic Proteomic Analysis of Pancreatic Mesenchyme Reveals Novel Factors That Enhance Human Embryonic Stem Cell to Pancreatic Cell Differentiation

**DOI:** 10.1155/2016/6183562

**Published:** 2015-11-22

**Authors:** Holger A. Russ, Limor Landsman, Christopher L. Moss, Roger Higdon, Renee L. Greer, Kelly Kaihara, Randy Salamon, Eugene Kolker, Matthias Hebrok

**Affiliations:** ^1^Diabetes Center, Department of Medicine, University of California, San Francisco, San Francisco, CA 94143, USA; ^2^Department of Cell and Developmental Biology, Faculty of Medicine, Tel Aviv University, 69978 Tel Aviv, Israel; ^3^Bioinformatics and High-Throughput Analysis Laboratory and High-Throughput Analysis Core, Center for Developmental Therapeutics, Seattle Children's Research Institute, Seattle, WA 98105, USA; ^4^Predictive Analytics, Seattle Children's Hospital, Seattle, WA 98105, USA; ^5^Data-Enabled Life Sciences Alliance (DELSA), Seattle, WA 98105, USA; ^6^Department of Biomedical Informatics & Medical Education and Pediatrics, Medical School, University of Washington, Seattle, WA 98101, USA

## Abstract

Current approaches in human embryonic stem cell (hESC) to pancreatic beta cell differentiation have largely been based on knowledge gained from developmental studies of the epithelial pancreas, while the potential roles of other supporting tissue compartments have not been fully explored. One such tissue is the pancreatic mesenchyme that supports epithelial organogenesis throughout embryogenesis. We hypothesized that detailed characterization of the pancreatic mesenchyme might result in the identification of novel factors not used in current differentiation protocols. Supplementing existing hESC differentiation conditions with such factors might create a more comprehensive simulation of normal development in cell culture. To validate our hypothesis, we took advantage of a novel transgenic mouse model to isolate the pancreatic mesenchyme at distinct embryonic and postnatal stages for subsequent proteomic analysis. Refined sample preparation and analysis conditions across four embryonic and prenatal time points resulted in the identification of 21,498 peptides with high-confidence mapping to 1,502 proteins. Expression analysis of pancreata confirmed the presence of three potentially important factors in cell differentiation: Galectin-1 (LGALS1), Neuroplastin (NPTN), and the Laminin *α*-2 subunit (LAMA2). Two of the three factors (LGALS1 and LAMA2) increased expression of pancreatic progenitor transcript levels in a published hESC to beta cell differentiation protocol. In addition, LAMA2 partially blocks cell culture induced beta cell dedifferentiation. Summarily, we provide evidence that proteomic analysis of supporting tissues such as the pancreatic mesenchyme allows for the identification of potentially important factors guiding hESC to pancreas differentiation.

## 1. Introduction

Generation of functional insulin-producing beta cells from human stem cell populations would provide an abundant resource for cell replacement therapies used in the treatment of type 1 diabetic patients. Several cell culture protocols detailing the guided differentiation of hESC [1, 2] and human induced pluripotent stem (iPS) cells [3, 4] into insulin-producing beta-like cells have been generated. However, most approaches have resulted in low numbers of immature, nonfunctional insulin expressing cells. A possible reason for the failure is the absence of critical factors present during embryonic pancreas development. Current approaches are largely based on knowledge gained from developmental studies into the pathways and transcriptional programs underlying murine pancreatic epithelium specification [1–4]. In contrast, the contribution of surrounding supportive tissues, including the mesenchyme critical for* in vivo* pancreas formation [3–5], has not been explored fully. Recent evidence underscoring the importance of the mesenchyme comes from coculture experiments demonstrating that mesenchymal cell lines promote the expansion of hESC-derived endocrine progenitors [6]; however, the factors responsible for these effects have not been identified. We hypothesized that a detailed proteomic characterization of factors produced by the pancreatic mesenchyme would result in the identification of proteins that could be added to current ES differentiation protocols to enable a more comprehensive simulation of normal development* in vitro*.

## 2. Experimental Methods

### 2.1. Mice

Mice used in this study were maintained according to protocols approved by the University of California, San Francisco, Committee on Laboratory Animal Resource Center.* Nkx3.2 (Bapx1)-*Cre mice were described previously [7].* R26-YFP* ^*flox*^ (*Gt(ROSA)26Sor* ^*tm1(EYFP)Cos*^) mice were obtained from Jackson Laboratories.

### 2.2. Sorting

Dissected pancreata were digested in 0.4 mg/mL Collagenase P (Roche) and 0.1 ng/mL DNase (Sigma) diluted in HBSS for 30 min at 37°C and filtered through a 40 mm filter. Following staining with PECAM1-PE (eBioscience, 1 : 200) and/or DAPI to exclude dead cells, cell isolation was performed using FACS Aria (BD).

### 2.3. Cell Culture

Undifferentiated CyT49 hES cells (ViaCyte, Inc.) were maintained on mouse embryo fibroblast feeder layers (Millipore). Differentiation was carried out as described previously [8]. LGALS1 (1 *μ*g/mL or 5 *μ*g/mL), LAMA2 (5 *μ*g/mL), and Narpin (3 *μ*M) were obtained from Peprotech (USA), Millipore (USA), and Bio Basics (Canada), respectively. Control fibroblast lines were established by culturing sorted YFP^+^ mesenchymal cells in DMEM containing 10% fetal bovine serum for at least 4 passages. For dedifferentiation analysis, isolated islets were dispersed into single cells by 5–10 min incubation with 0.25% Trypsin (Gibco), followed by cell filtration. Cells were grown for 3 days either on poly-D-Lysine coated-plates (Millipore) or on plates coated overnight with 5 *μ*g/mL human Merosin (Millipore), which is comprised of a mixture of LM211 and LM221.

### 2.4. Immunofluorescence Analysis

Fixed human cadaver pancreatic tissue (Prodo) was incubated in 30% sucrose solution followed by embedding in OCT (TissueTek), cryopreservation, and sectioning. Dissected mouse pancreatic tissues were fixed with Z-fix (Anatech), incubated in 30% sucrose overnight, and cryopreserved. Tissue sections were stained with primary antibodies, anti-mouse vimentin (1 : 200, Sigma), anti-human vimentin (1 : 50, Calbiochem), anti-YFP/GFP (1 : 500, Abcam), anti-Galectin-1 (1 : 50, RnD Systems), anti-LAMA2 (1 : 200, Enzo), and anti-PDX1 (1 : 200, RnD Systems), followed by staining with AlexaFluor tagged secondary antibodies (1 : 500, Invitrogen) and mounting Vectashield media (Vector). Nuclei were visualized with DAPI. Images were acquired using a Leica SP5 microscope or an InCell Analyzer 2000 for quantification (GE Healthcare). 16 fields from each condition were randomly selected by the InCell Analyzer and imaged for quantification. PDX1 positive nuclei over total nuclei were determined using InCell Developer software (GE Healthcare).

### 2.5. qPCR Analysis

Total RNA was isolated with TRIZOL (Sigma) and 500 ng was reverse transcribed using the iScript cDNA Kit (Bio-Rad) according to manufacturer's instructions. qPCR analysis was performed on an ABI 7900 HT Fast Real-Time PCR System (Applied Biosystems) using standard protocols. Primer sequences are as follows: LGALS1 ATCGTGTGCAACAGCAAGG and CCTGGTCGAAGGTGATGC; LAMA2 AAAGATCCTTCCAAGAACAAAATC and CGGTCAGCTTCCTGTTCTAAA; NPTN TCTCGCTGTTGCTGGTCTC and CCTCTTCACTGGTGACAATCC; NGN3 AAGTCTACCAAAGCTCACGCG and GCTCATCGCTCTCTATTCTTTTGC; PDX1 AAGTCTACCAAAGCTCACGCG and GTAGGCGCCGCCTGC; TBP TGTGCACAGGAGCCAAGAGT and ATTTTCTTGCTGCCAGTCTGG. Taqman Probes: LAMA2 Mm00550083_m1; ACTB 4352933-0810025.

### 2.6. MS Methods

Filter-Aided Sample Prep (FASP) was used to lyse cells, digest with trypsin, and recover the tryptic peptides [9]. Peptides were analyzed by nano-LC/MS/MS. Peptides were eluted across a 3-hour gradient on a 20 cm, self-packed C18 capillary column on an Eksigent NanoLC-2D system. Mass spectra were acquired on a Thermo LTQ Velos linear ion-trap mass spectrometer. The 5 highest intensity 2^+^ or 3^+^ ions from each MS scan were selected for collision induced dissociation (CID).

### 2.7. Data Analysis

Protein identification and quantitation were carried out using the SPIRE (Systematic Protein Identification and Relative Expression) analysis platform for high-throughput proteomics data analysis [10]. SPIRE employs a novel approach to combining the open source search algorithms X! Tandem and OMSSA to increase the number of peptide and protein identifications and significantly improve statistical assessment of these identifications. SPIRE generates peptide identification probabilities based on a combination of logistic regression models and a randomized protein sequence database search, in this case versus the Uniprot complete mouse proteome. SPIRE uses a newly modified protein ID model to aggregate peptide spectra identifications into protein identifications and generate a false discovery rate threshold value (*q*-value) for each identified protein. SPIRE also generates quantitation estimates for relative expression analysis based upon spectral counts (number of high-confidence peptide spectrum matches) normalized by total expression in each sample. Visualization of the proteomics expression data was carried out by a combination of heatmaps and hierarchical clustering using R statistical software (R foundation for statistical computing). The protein identifications were mapped to functional keywords in the Gene Ontology using amiGO [11]. The data were revisualized as separated protein groups based upon these results. Human ES differentiation data are shown as the mean  ±  SD. Data were evaluated using a two-tailed Student's *t*-test.

### 2.8. Targeted Proteomics

Laminin alpha-2 was quantified in samples using selected reaction monitoring (SRM) coupled with stable isotope dilution mass spectrometry (SID-MS) [12]. Two peptides identified in spectral libraries from previous experiments were used as characteristic peptides for Laminin alpha-2. We had these peptides synthesized and isotopically labeled. The highest intensity y-ions for each peptide from the MS/MS spectra in the spectral library were chosen as reporter ions to determine concentration. Calibration curves of reporter ion intensity ratios were determined for the two different synthesized peptides and their isotope-labeled partners. The isotope-labeled synthesized peptide standards were spiked into samples at 0.5 fmol/*μ*L; then absolute concentration of target peptides was determined by fitting isotope ratios of the MS/MS reporter ions to the calibration curve. Skyline analysis software was used to set up inclusion lists for peptide sequences extract peak intensity values and provide spectral libraries [13].

## 3. Results and Discussion

**Figure 1:**
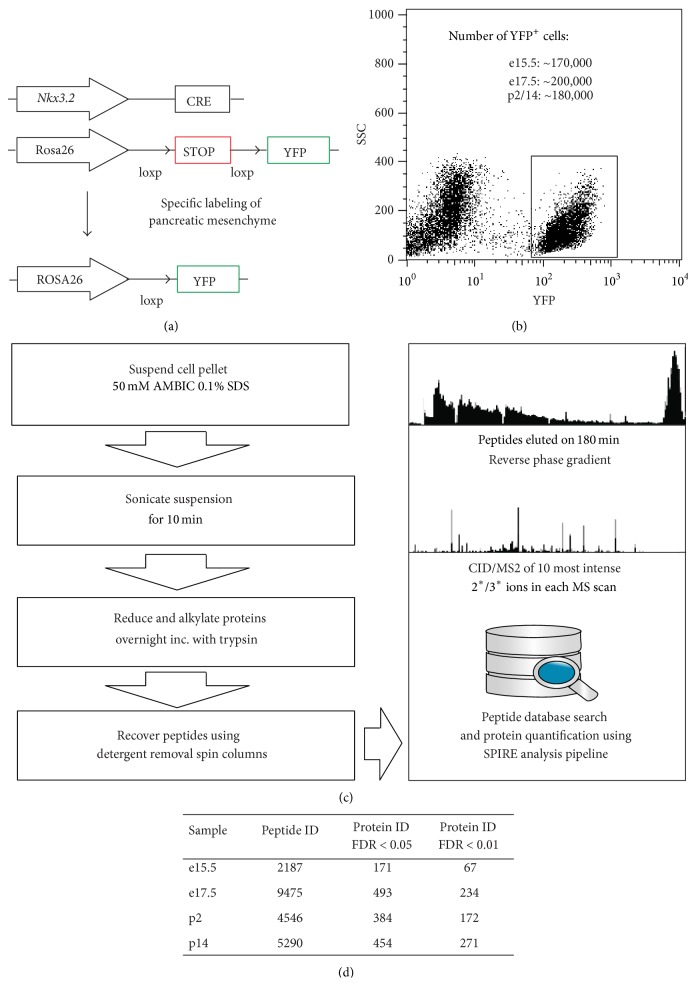
Isolation of pancreatic mesenchyme for proteomic analysis. (a) Schematic representation of transgenic mouse models employed to permanently label pancreatic mesenchymal cells. The mesenchyme specific* Nkx3.2* promoter drives Cre recombinase expression during organogenesis and results in the excision of a loxp site flanked stop sequence. This excision in turn gives rise to constitutive expression of the fluorescence reporter YFP specifically in mesenchymal cells. (b) Representative dot plot of fluorescence activated cell sorting of YFP labeled mesenchymal cells. Approximate numbers of sorted YFP^+^ cells per litter and postpartum mouse are given in the inset. (c) Schematic illustrating the sample preparation and mass spectrometric analysis. (d) Table with total number of identified peptides per time point and corresponding protein discoveries with false discovery rates (FDR) of 5% and 1%, respectively.

**Figure 2:**
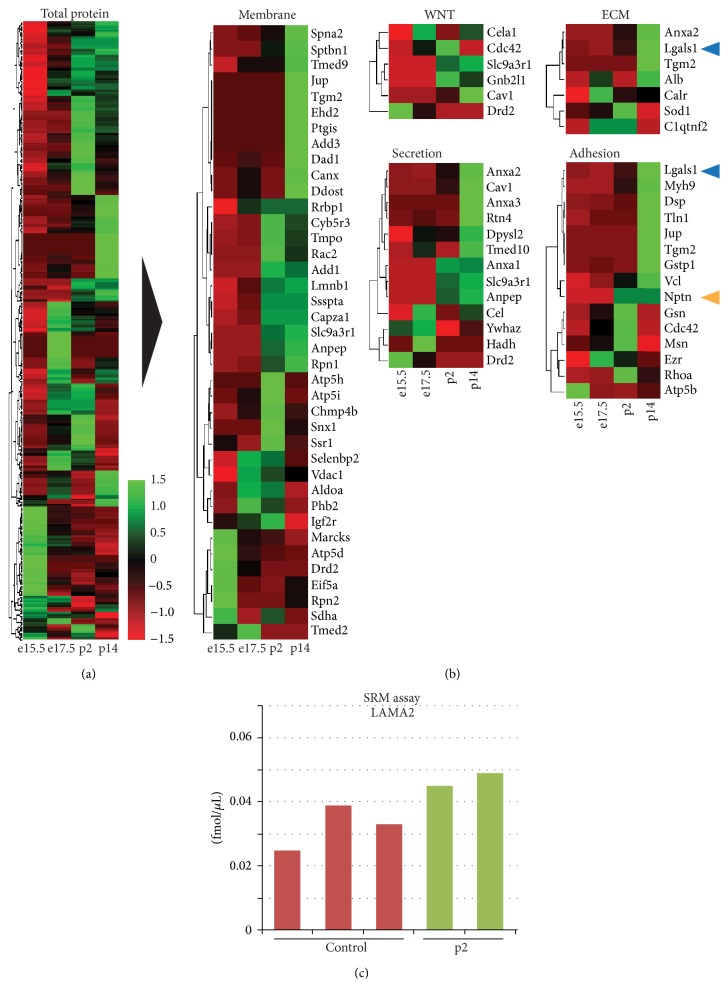
Expression pattern analysis of mesenchymal proteome and absolute quantification of candidate protein Laminin *α*-2. ((a) and (b)) Identified proteins were visualized as heat maps with high and low expression visualized in green or red, respectively. Expression values were normalized to *z* scores by subtracting raw means and dividing by standard deviations. (a) All proteins with at least 5 peptide spectrum matches are visualized. (b) Proteins were further broken down by GO terms: membrane, WNT pathway, extracellular matrix (ECM), secretion, and adhesion. Two factors, Galectin-1 (LGALS1, blue arrowheads) and Neuroplastin (NPTN, yellow arrowhead), were chosen for further analysis. (c) Absolute quantification of Laminin *α*-2 chain (LAMA2) in p2 samples by selected reaction monitoring (SRM) revealed a concentration of 0.47 fmol/*μ*L in comparison to 0.30 fmol/*μ*L in control fibroblast samples.

**Figure 3:**
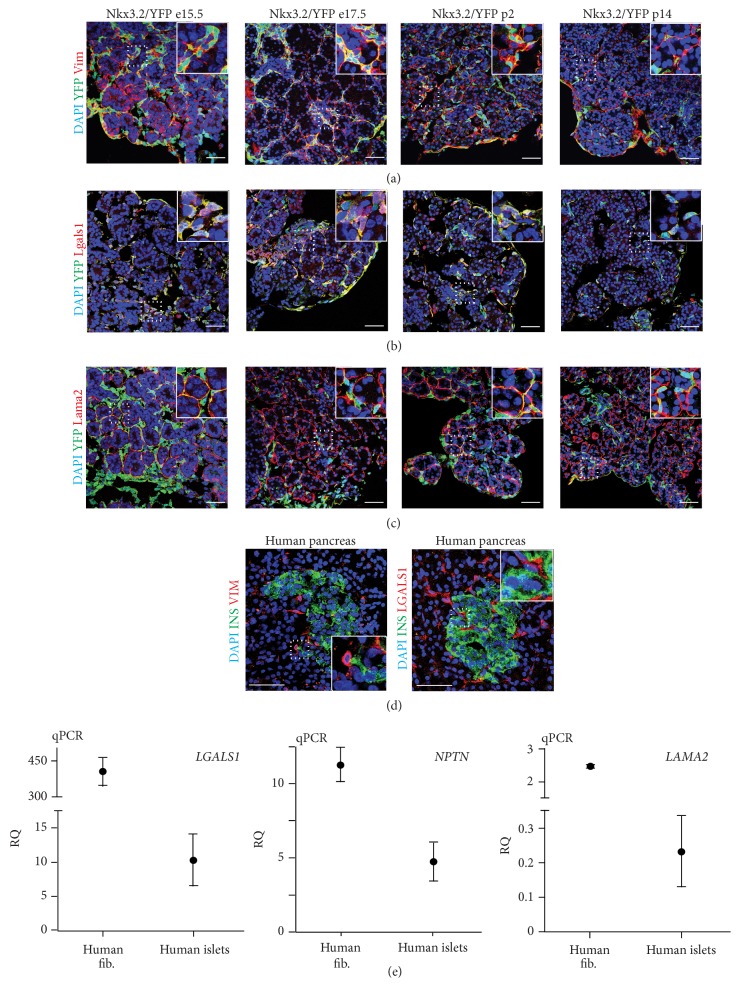
Mesenchymal factors, Galectin-1, Neuroplastin, and Laminin *α*-2, are expressed in mouse and human pancreata. ((a), (b), and (c)) Coimmunofluorescence analysis of YFP linage-traced transgenic pancreata at e15.5, e17.5, p2, and p14 for coexpression of YFP marker, the common mesenchymal marker Vimentin (Vim, in (a)), Galectin-1 (Lgals1, in (b)), and Laminin *α*-2 (Lama2, in (c)). Size bar = 50 *μ*m. (d) Healthy human pancreas sections showing vimentin (VIM) and galectin-1 (LGALS1) positive cells with mesenchymal morphology intermingled and in close proximity to insulin expressing beta cells organized in islets of Langerhans. Size bar = 50 *μ*m. (e) Quantitative PCR analysis of Galectin-1 (*LGALS1*), Neuroplastin (*NPTN*), and Laminin *α*-2 (*LAMA2*) transcripts in human foreskin fibroblasts (*n* = 2) and purified human islet preparations (*n* = 5).

In our proteomic analysis we employed the previously described* Nkx*3.2-Cre mouse line, which permits specific expression of Cre recombinase in mesenchyme, but not epithelial, endothelial, or neuronal cells of the developing pancreas [5]. This mouse line was used in conjunction with a Rosa26 loxp STOP loxp yellow fluorescence protein (YFP) reporter mouse to isolate pancreatic mesenchymal cells by fluorescence activated cell sorting (FACS) for global proteomic analysis (Figures 1(a) and 1(b)). YFP^+^ mesenchymal cell aliquots of ~500,000 cells from embryonic day (e)15.5 (peak of Ngn3 expression, a marker for endocrine cell differentiation), e17.5 (islet formation initiated), postnatal day (p)2 (immature beta cells present), and p14 (mature beta cells present) pancreata with purities of 96.8 ± 1.6% (see Figure S1 in Supplementary Material available online at http://dx.doi.org/10.1155/2016/6183562) were sorted under protein-free conditions and snap frozen prior to analysis by tandem MS based shotgun proteomics. Notably, the sample amount obtained was an order of magnitude lower than what is typically employed in such experiments. Given this constraint we used a protocol (Figure 1(c)) that minimizes necessary sample clean-up and resulted in the recovery of 8.6 to 16.3 *μ*g of protein from each aliquot. Aliquots were analyzed in triplicate and protein identification and quantification were carried out using SPIRE software [10] and peptide spectra were matched to the UniProt [14] mouse complete proteome sequence database. Overall, we identified 21,498 high-confidence (greater than 90%) peptide spectrum matches across time points using our approach (Figure 1(d)). Using SPIRE generated false discovery rate (FDR) thresholds of either 1% or 5% resulted in the identification of 842 and 1502 proteins, respectively (Figure 1(d)). Visualization using heatmaps of the normalized spectral counts and hierarchical clustering revealed distinct sets of proteins based on expression changes over time (Figure 2(a)). Further filtering of proteins by Gene Ontology (GO) [11] terms including extracellular matrix (ECM), secretion, adhesion, wingless signaling transduction pathway (WNT), and membrane localization resulted in matches to 143 total proteins. From these, we chose two, Galectin-1 (LGALS1) and Neuroplastin (NPTN) (Figure 2(b)), for further evaluation, based on their expression patterns and information from published studies [15, 16]. Although the approach we employed is an excellent strategy for evaluating a large number of protein candidates, many very low abundance proteins still remain below the detection limit. Given this caveat, we also pursued a targeted approach based on preliminary results suggesting a role for Laminins 211 and 221 containing the *α*-2 subunit (LAMA2) in beta cell function. We had noted that FACS sorted YFP^+^ mesenchymal cells revealed a significant enrichment of* Lama2* transcripts compared to whole pancreas. Of note,* Lama2* was not detected in pancreatic endothelial cells (Figure S2A and [17]), suggesting the mesenchyme as the primary source of Laminin *α*-2 chain in the pancreas. Addition of LAMA2 partially blocks cell culture induced dedifferentiation of beta cells (Figure S2B). mRNA transcript levels for beta cell marker genes* Ins1* and* Mafa* in cultured islets treated with LAMA2 were unchanged compared to fresh islets, while Lysine treated control cultures showed a significant reduction. Glucose transporter* Glut2* gene expression was reduced in both cultured islet groups compared to fresh islets, albeit significantly less in LAMA2 treated cultured islets. In addition,* Hes1*, a marker for beta cell dedifferentiation [18], was less induced in LAMA2 treated islets compared to control cultures (Figure S2B). Collectively, these data indicate a functional role for LAMA2 in supporting the beta cell differentiation state. We designed a selected reaction-monitoring (SRM) assay using two synthesized peptide standards from LAMA2 and determined the mean concentration of protein present in three independent fibroblast cell lines established from sorted YFP^+^ mesenchymal cells and in two samples of freshly sorted P2 neonatal YFP^+^ cells to be 0.032 and 0.047 fmol/*μ*L, respectively (Figure 2(c)). Notably, concentrations are an order of magnitude below what is typically detected in shotgun proteomics experiments using the instrumentation employed in this study [19]. Tandem MS and SRM results were confirmed by employing immunofluorescence analysis of lineage-traced YFP^+^ mesenchymal cells of pancreata from e15.5, e17.5, p2, and p14. As expected, YFP^+^ cells costain for the mesenchymal marker Vimentin (Vim) that was used as a positive control (Figure 3(a)). These cells also exhibit immune reactivity to an LGALS1 specific antibody, confirming the proteomic identification of this factor in pancreas mesenchyme (Figure 3(b)). Staining by Lama2 revealed an intricate network in close proximity to YFP-labeled mesenchymal cells (Figure 3(c)). We also detected cells positive for the mesenchymal marker VIM or LGALS1 with a typical mesenchymal morphology immediately adjacent to insulin-producing beta cells. These putative mesenchymal cells can be found both surrounding and within islets of healthy human pancreata (Figure 3(d)). Although we could not achieve reliable staining with existing antibodies for LAMA2 in human pancreas sections and NPTN in mouse or human sections, qPCR analysis of purified human islets revealed on average 0.2- and 5-fold expression of the* LAMA2* and* NPTN* transcripts, respectively, over the endogenous control gene TATA box binding protein (*TBP*) (Figure 3(e)). Cultured human fibroblasts expressed on average 2.5- and 11-fold more* LAMA2* and* NPTN*, respectively.* LGALS1* transcripts in cultured human fibroblasts and purified human islets were on average 406- and 10-fold enriched, respectively (Figure 3(e)). These results confirm the presence of the factors identified by proteomic analysis in mouse and human islet tissues.

**Figure 4:**
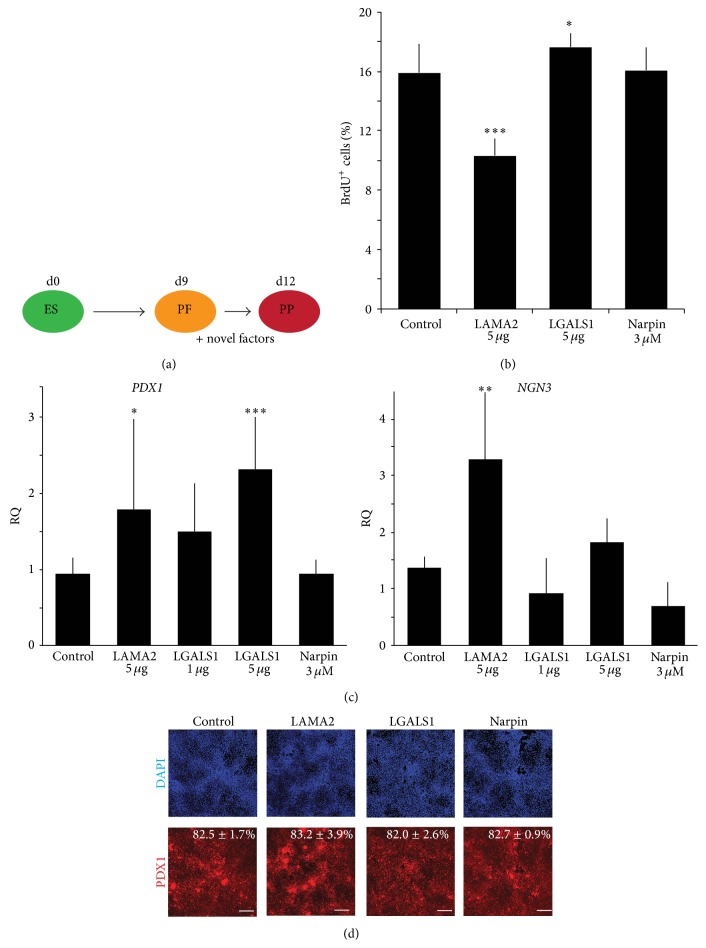
Mesenchymal factors, Laminins containing a *α*-2 chain and Galectin-1, improve human embryonic stem cell differentiation. (a) Schematic of the experimental setup to test possible effects of novel mesenchymal factors on direct differentiation of human embryonic stem (ES) cells. Factors were added at day 9 to posterior foregut-like (PF) cells for three days before analysis of pancreatic progenitor (PP) cells. (b) Flow cytometric analysis of proliferating cells in control and treated cultures at day 12 after 24-hour incubation with BrdU. Data shown are from 2 independent experiments, *n* = 7. (c) Quantitative PCR analysis for the pancreatic progenitor marker* PDX1* and endocrine marker* NGN3*. Data shown are average ± standard deviation from 3 independent experiments, *n* = 5–8, relative to untreated control cultures at day 12 of differentiation and normalized to the endogenous control gene* TBP*. (d) Quantification of immunofluorescence analysis of PDX1 positive as a percentage of total cells with an InCell Analyzer. Data shown are average ± standard deviation of 16 independent fields assayed in 2 independent experiments. Size bar = 250 *μ*m.

Next we examined whether our novel factors could promote directed differentiation of hESCs towards pancreatic endocrine cells employing our recently published protocol [8]. Analysis by qPCR revealed very low or undetectable transcript levels for* LAMA2* and* LGALS1* in control differentiated hESC cultures at the pancreatic progenitor stage. In contrast,* NPTN* transcripts levels, albeit lower than in cultured fibroblast or islets, were on average 1.3-fold higher than* TBP* levels (Figure S3). We added recombinant LGALS1 and LAMA2, as well as the peptide Narpin (shown to mimic the binding activity of NPTN* in vitro* [16]), to hESC-derived foregut-like cells for three days to allow further differentiation into pancreatic progenitors (Figure 4(a)). We observed significant differences relative to control cultures in the proliferation of cells treated with either LAMA2 or LGALS1 (Figure 4(b)). We previously showed that mesenchymal cells support proliferation of pancreatic precursors and later staged differentiated endocrine cells* in vivo* [5]. While LGALS1 addition resulted in a significant increase of proliferation, LAMA2 reduced proliferation of pancreatic progenitors. These data indicate discrete effects of individual mesenchyme factors at specific developmental stages and that such might differ from the combined effects of all factors provided by a supportive tissue, like the pancreatic mesenchyme, during development* in vivo*. Pancreatic progenitor marker* PDX-1* mRNA levels were significantly increased in experiments incubated with either LGALS1 or LAMA2 while Narpin incubation did not show any changes (Figure 4(c)). The observed increase in* PDX-1* transcript levels after addition of LGALS was concentration dependent. However, quantification of pancreatic progenitor cells identified by PDX-1 protein expression did not reveal significant changes after treatment with LGALS1, LAMA2, or Narpin (Figure 4(d)), indicating that the factors promote PDX-1 expression without increasing the already high percentage of PDX-1+ cells present under control conditions. In addition, LAMA2 enhanced transcript levels of the endocrine progenitor marker* NGN3* (Figure 4(c)), known to be required for the generation of all hormone positive endocrine cells [20].

In summary our data demonstrate the ability to identify novel factors potentially important in beta cell differentiation using proteomic analysis of the pancreatic mesenchyme. In addition, we show that specific proteins can be quantified by our approach in low abundance samples. Most importantly, we show that two out of three novel factors selected for additional validation can enhance hESC to beta cell differentiation. Our approach described here not only is restricted to the pancreas but also can be easily adopted to better characterize even small numbers of supportive cell types and tissues in differentiation of other lineages and organs. We anticipate that fine-tuning existing hESC differentiation approaches will enable the generation of fully matured beta cells under cell culture conditions.

## Supplementary Material

Supplementary Table 1: Spreadsheet containing all and GO term proteomic results.
Supplementary Figure 2S: A: RNA was isolated from bulk pancreatic tissues (total pancreas), pancreatic endothelial cells (Pecam1+ cells, isolated by FACS from total pancreata) and pancreatic mesenchymal cells (Nkx3.2/YFP+ cells, isolated by FACS from Nkx3.2-Cre;YFP total pancreata). Expression levels were analyzed by qPCR. N = 4. ∗∗∗P < 0.005 as compared to total untreated pancreata. ND = Not detected. B: Treatment with LAMA2 partially inhibits culture-induced beta-cell dedifferentiation. RNA was extracted from freshly isolated islets (white) or from trypsin-dispersed islet cells after 3 days of culture on plates coated with either Poly-D-Lysine (blue) or human Merosin (a mixture of Laminin-211 and -221, green). N = 4. Data show one representative of two independent experiments with comparable results. ∗P < 0.05, ∗∗P < 0.01, ∗∗∗P < 0.005, NS = non significant, as compared to freshly isolated islets.
Supplementary Figure 3S: Galectin-1 and α-2 chain Laminins are expressed only at very low levels after 12 days of differentiation of hES cells compared to human fibroblasts and islets while Neuroplastin is readily detectable. Quantitative PCR analysis of LGALS1, NPTN and LAMA2 transcripts in human foreskin fibroblasts (n=2) and purified human islet preparations (n=5) (as shown in figures 2&3) and hES after 12 days of differentiation (n=4, 2 independent experiments). Expression values are shown as average fold change ± standard deviation compared to expression levels of the endogenous control gene TBP.
