# Supplementary material for: Dynamic Proteomic Analysis of Pancreatic Mesenchyme Reveals Novel Factors That Enhance Human Embryonic Stem Cell to Pancreatic Cell Differentiation
Source: Stem Cells Int. 2015 Nov 22;2016:6183562. doi: 10.1155/2016/6183562 (PMC4670689; doi:10.1155/2016/6183562)
Supplement: Supplementary file 1 — Supplementary Table 1: Spreadsheet containing all and GO term proteomic results. Supplementary Figure 2S: A: RNA was isolated from bulk pancreatic tissues (total pancreas), pancreatic endothelial cells (Pecam1+ cells, isolated by FACS from total pancreata) and pancreatic mesenchymal cells (Nkx3.2/YFP+ cells, isolated by FACS from Nkx3.2-Cre;YFP total pancreata). Expression levels were analyzed by qPCR. N = 4. ∗∗∗P < 0.005 as compared to total untreated pancreata. ND = Not detected. B: Treatment with LAMA2 partially inhibits culture-induced beta-cell dedifferentiation. RNA was extracted from freshly isolated islets (white) or from trypsin-dispersed islet cells after 3 days of culture on plates coated with either Poly-D-Lysine (blue) or human Merosin (a mixture of Laminin-211 and -221, green). N = 4. Data show one representative of two independent experiments with comparable results. ∗P < 0.05, ∗∗P < 0.01, ∗∗∗P < 0.005, NS = non significant, as compared to freshly isolated islets. Supplementary Figure 3S: Galectin-1 and α-2 chain Laminins are expressed only at very low levels after 12 days of differentiation of hES cells compared to human fibroblasts and islets while Neuroplastin is readily detectable. Quantitative PCR analysis of LGALS1, NPTN and LAMA2 transcripts in human foreskin fibroblasts (n=2) and purified human islet preparations (n=5) (as shown in figures 2&3) and hES after 12 days of differentiation (n=4, 2 independent experiments). Expression values are shown as average fold change ± standard deviation compared to expression levels of the endogenous control gene TBP. [file 6183562.f1.pdf]

		FDR			
protein	annotation	e15.5	e17.5	p2	p14
A2ABG4	TBC1 domain family, member 16 OS=Mus musculus GN=Tbc1d16 PE=4 SV=1	1.00	1.00	0.05	1.00
A2ARZ3	Fibrous sheath-interacting protein 2 OS=Mus musculus GN=Fsip2 PE=1 SV=3	1.00	0.05	1.00	1.00
A2ASQ1	Agrin OS=Mus musculus GN=Agrn PE=2 SV=1	0.07	1.00	1.00	1.00
A8DUK4	Beta-globin OS=Mus musculus GN=Hbb-b1 PE=3 SV=1	1.00	0.00	0.00	0.00
B2RS76	Carboxypeptidase B1 (Tissue) OS=Mus musculus GN=Cpb1 PE=2 SV=1	1.00	0.00	0.00	0.00
B2RX14	Terminal uridylyltransferase 4 OS=Mus musculus GN=Zcchc11 PE=1 SV=2	1.00	1.00	1.00	0.06
B7ZNL3	Tpm1 protein OS=Mus musculus GN=Tpm1 PE=2 SV=1	1.00	0.03	0.02	0.02
D3YY62	Uncharacterized protein OS=Mus musculus GN=Gm9858 PE=4 SV=1	0.03	0.03	0.02	1.00
D3Z5Y1	Uncharacterized protein OS=Mus musculus GN=Stard13 PE=4 SV=1	0.03	1.00	1.00	1.00
D3Z6P0	Protein disulfide-isomerase A2 OS=Mus musculus GN=Pdia2 PE=1 SV=1	1.00	0.02	0.00	0.00
E9PUV7	Uncharacterized protein OS=Mus musculus GN=4930485B16Rik PE=4 SV=1	1.00	0.08	1.00	1.00
E9PVM7	Uncharacterized protein OS=Mus musculus GN=Gstm5 PE=3 SV=1	1.00	1.00	1.00	0.03
E9PVZ3	Uncharacterized protein OS=Mus musculus GN=Ccdc144b PE=4 SV=1	1.00	1.00	1.00	0.04
E9PZQ0	Uncharacterized protein OS=Mus musculus GN=Ryr1 PE=4 SV=1	1.00	0.07	1.00	0.04
E9Q191	Uncharacterized protein OS=Mus musculus GN=Olfr288 PE=3 SV=1	1.00	1.00	0.08	1.00
E9Q1Y9	Uncharacterized protein OS=Mus musculus GN=5430421N21Rik PE=3 SV=1	1.00	0.00	1.00	1.00
E9Q447	Uncharacterized protein OS=Mus musculus GN=Spna2 PE=4 SV=1	1.00	0.00	0.00	0.00
E9Q455	Uncharacterized protein OS=Mus musculus GN=Tpm1 PE=3 SV=1	1.00	0.03	0.02	0.00
E9Q557	Desmoplakin OS=Mus musculus GN=Dsp PE=3 SV=1	1.00	0.00	0.00	0.00
E9Q616	Uncharacterized protein OS=Mus musculus GN=Ahnak PE=4 SV=1	1.00	0.00	0.00	0.00
E9Q7L1	Uncharacterized protein OS=Mus musculus GN=Urb2 PE=4 SV=1	0.01	0.01	0.02	1.00
E9Q9C6	Uncharacterized protein OS=Mus musculus GN=Fcgbp PE=4 SV=1	1.00	0.03	1.00	1.00
F6Q3G7	Uncharacterized protein (Fragment) OS=Mus musculus GN=Upf2 PE=4 SV=1	0.03	0.03	1.00	1.00
F6R5A0	Uncharacterized protein (Fragment) OS=Mus musculus GN=D1Ertd622e PE=4 SV=1	1.00	0.04	1.00	1.00
F6VBB8	Uncharacterized protein (Fragment) OS=Mus musculus PE=4 SV=1	0.02	0.03	0.03	1.00
F6W6S4	Uncharacterized protein (Fragment) OS=Mus musculus GN=Hpn PE=4 SV=1	1.00	1.00	0.04	1.00
F7DBB3	Uncharacterized protein (Fragment) OS=Mus musculus GN=Ahnak2 PE=4 SV=1	1.00	1.00	1.00	0.00
O08547	Vesicle-trafficking protein SEC22b OS=Mus musculus GN=Sec22b PE=1 SV=3	1.00	1.00	1.00	0.02
O08553	Dihydropyrimidinase-related protein 2 OS=Mus musculus GN=Dpysl2 PE=1 SV=2	1.00	0.02	0.02	0.00
O08749	Dihydrolipoyl dehydrogenase, mitochondrial OS=Mus musculus GN=Dld PE=1 SV=2	1.00	1.00	0.08	1.00
O08795	Glucosidase 2 subunit beta OS=Mus musculus GN=Prkcsh PE=1 SV=1	0.02	0.00	1.00	0.06
O08807	Peroxiredoxin-4 OS=Mus musculus GN=Prdx4 PE=1 SV=1	1.00	0.00	0.02	1.00
O35074	Prostacyclin synthase OS=Mus musculus GN=Ptgis PE=2 SV=1	1.00	1.00	1.00	0.00
O35098	Dihydropyrimidinase-related protein 4 OS=Mus musculus GN=Dpysl4 PE=1 SV=1	1.00	1.00	1.00	0.02
O35129	Prohibitin-2 OS=Mus musculus GN=Phb2 PE=1 SV=1	1.00	0.02	0.02	1.00
O35326	Serine/arginine-rich splicing factor 5 OS=Mus musculus GN=Srsf5 PE=1 SV=1	1.00	1.00	0.02	1.00
O35350	Calpain-1 catalytic subunit OS=Mus musculus GN=Capn1 PE=2 SV=1	1.00	1.00	1.00	0.07
O35381	Acidic leucine-rich nuclear phosphoprotein 32 family member A OS=Mus musculus GN=Anp32a PE=1 SV=1	1.00	0.00	1.00	1.00
O35639	Annexin A3 OS=Mus musculus GN=Anxa3 PE=1 SV=4	1.00	1.00	1.00	0.00
O35640	Annexin A8 OS=Mus musculus GN=Anxa8 PE=2 SV=2	1.00	1.00	1.00	0.05
O35682	Myeloid-associated differentiation marker OS=Mus musculus GN=Myadm PE=2 SV=2	1.00	1.00	1.00	0.02
O35685	Nuclear migration protein nudC OS=Mus musculus GN=Nudc PE=1 SV=1	1.00	0.04	1.00	1.00
O35855	Branched-chain-amino-acid aminotransferase, mitochondrial OS=Mus musculus GN=Bcat2 PE=2 SV=2	1.00	0.03	1.00	1.00
O35887	Calumenin OS=Mus musculus GN=Calu PE=1 SV=1	1.00	0.00	0.00	0.00
O54724	Polymerase I and transcript release factor OS=Mus musculus GN=Ptrf PE=1 SV=1	1.00	1.00	0.02	0.00
O54734	Dolichyl-diphosphooligosaccharide--protein glycosyltransferase 48 kDa subunit OS=Mus musculus GN=Ddost PE	1.00	0.02	1.00	0.00
O54754	Aldehyde oxidase OS=Mus musculus GN=Aox1 PE=2 SV=2	0.04	1.00	1.00	1.00
O54879	High mobility group protein B3 OS=Mus musculus GN=Hmgb3 PE=2 SV=3	1.00	1.00	0.02	1.00
O54962	Barrier-to-autointegration factor OS=Mus musculus GN=Banf1 PE=1 SV=1	0.02	1.00	1.00	1.00
O55022	Membrane-associated progesterone receptor component 1 OS=Mus musculus GN=Pgrmc1 PE=1 SV=4	1.00	0.02	1.00	1.00
O55023	Inositol monophosphatase 1 OS=Mus musculus GN=Impa1 PE=1 SV=1	1.00	1.00	1.00	0.07
O55026	Ectonucleoside triphosphate diphosphohydrolase 2 OS=Mus musculus GN=Entpd2 PE=1 SV=2	1.00	1.00	1.00	0.00
O55029	Coatomer subunit beta' OS=Mus musculus GN=Copb2 PE=2 SV=2	1.00	1.00	1.00	0.02
O55131	Septin-7 OS=Mus musculus GN=Sept7 PE=1 SV=1	1.00	1.00	0.00	0.02
O55142	60S ribosomal protein L35a OS=Mus musculus GN=Rpl35a PE=2 SV=2	1.00	1.00	0.02	0.02
O55234	Proteasome subunit beta type-5 OS=Mus musculus GN=Psmb5 PE=1 SV=3	1.00	0.02	0.00	1.00
O70251	Elongation factor 1-beta OS=Mus musculus GN=Eef1b PE=1 SV=5	0.02	0.00	1.00	0.00
O70318	Band 4.1-like protein 2 OS=Mus musculus GN=Epb41l2 PE=1 SV=1	1.00	0.05	1.00	1.00
O70435	Proteasome subunit alpha type-3 OS=Mus musculus GN=Psma3 PE=1 SV=3	1.00	0.02	0.02	1.00
O70456	14-3-3 protein sigma OS=Mus musculus GN=Sfn PE=1 SV=2	1.00	1.00	0.02	1.00
O70475	UDP-glucose 6-dehydrogenase OS=Mus musculus GN=Ugdh PE=1 SV=1	1.00	0.00	0.00	0.00
O70492	Sorting nexin-3 OS=Mus musculus GN=Snx3 PE=1 SV=3	1.00	1.00	0.05	1.00
O88342	WD repeat-containing protein 1 OS=Mus musculus GN=Wdr1 PE=1 SV=3	1.00	1.00	0.06	1.00
O88456	Calpain small subunit 1 OS=Mus musculus GN=Capns1 PE=2 SV=1	1.00	1.00	1.00	0.02
O88487	Cytoplasmic dynein 1 intermediate chain 2 OS=Mus musculus GN=Dync1i2 PE=2 SV=1	1.00	0.00	1.00	1.00
O88569	Heterogeneous nuclear ribonucleoproteins A2/B1 OS=Mus musculus GN=Hnrnpa2b1 PE=1 SV=2	0.00	0.00	0.00	0.00
O88844	Isocitrate dehydrogenase [NADP] cytoplasmic OS=Mus musculus GN=Idh1 PE=1 SV=2	1.00	0.00	0.00	0.00
O88962	7-alpha-hydroxycholest-4-en-3-one 12-alpha-hydroxylase OS=Mus musculus GN=Cyp8b1 PE=2 SV=1	1.00	1.00	1.00	0.07
O89086	Putative RNA-binding protein 3 OS=Mus musculus GN=Rbm3 PE=1 SV=1	0.03	0.02	0.02	1.00

P00683	Ribonuclease pancreatic OS=Mus musculus GN=Rnase1 PE=1 SV=2	1.00	0.02	0.02	1.00
P00688	Pancreatic alpha-amylase OS=Mus musculus GN=Amy2 PE=1 SV=2	1.00	0.00	0.00	0.00
P04104	Keratin, type II cytoskeletal 1 OS=Mus musculus GN=Krt1 PE=1 SV=4	1.00	0.02	1.00	0.02
P04117	Fatty acid-binding protein, adipocyte OS=Mus musculus GN=Fabp4 PE=1 SV=3	1.00	1.00	1.00	0.00
P05064	Fructose-bisphosphate aldolase A OS=Mus musculus GN=Aldoa PE=1 SV=2	0.02	0.00	0.00	0.00
P05202	Aspartate aminotransferase, mitochondrial OS=Mus musculus GN=Got2 PE=1 SV=1	1.00	1.00	0.02	1.00
P05208	Chymotrypsin-like elastase family member 2A OS=Mus musculus GN=Cela2a PE=2 SV=1	1.00	0.00	0.00	0.00
P05213	Tubulin alpha-1B chain OS=Mus musculus GN=Tuba1b PE=1 SV=2	0.00	1.00	1.00	0.00
P06151	L-lactate dehydrogenase A chain OS=Mus musculus GN=Ldha PE=1 SV=3	0.02	0.00	0.00	0.00
P06745	Glucose-6-phosphate isomerase OS=Mus musculus GN=Gpi PE=1 SV=4	1.00	1.00	0.00	0.02
P07146	Anionic trypsin-2 OS=Mus musculus GN=Prss2 PE=2 SV=1	1.00	0.00	0.02	0.00
P07356	Annexin A2 OS=Mus musculus GN=Anxa2 PE=1 SV=2	0.00	0.00	0.00	0.00
P07724	Serum albumin OS=Mus musculus GN=Alb PE=1 SV=3	1.00	0.02	1.00	0.00
P07901	Heat shock protein HSP 90-alpha OS=Mus musculus GN=Hsp90aa1 PE=1 SV=4	0.00	0.00	0.00	0.00
P08003	Protein disulfide-isomerase A4 OS=Mus musculus GN=Pdia4 PE=1 SV=3	0.00	0.00	0.00	0.00
P08113	Endoplasmin OS=Mus musculus GN=Hsp90b1 PE=1 SV=2	0.00	0.00	0.00	0.00
P08207	Protein S100-A10 OS=Mus musculus GN=S100a10 PE=2 SV=2	1.00	0.02	0.02	0.00
P08228	Superoxide dismutase [Cu-Zn] OS=Mus musculus GN=Sod1 PE=1 SV=2	0.02	0.00	0.00	0.00
P08249	Malate dehydrogenase, mitochondrial OS=Mus musculus GN=Mdh2 PE=1 SV=3	1.00	0.00	0.00	0.00
P08730	Keratin, type I cytoskeletal 13 OS=Mus musculus GN=Krt13 PE=1 SV=2	0.03	1.00	1.00	0.02
P08752	Guanine nucleotide-binding protein G(i) subunit alpha-2 OS=Mus musculus GN=Gnai2 PE=1 SV=5	1.00	1.00	1.00	0.02
P09055	Integrin beta-1 OS=Mus musculus GN=Itgb1 PE=1 SV=1	1.00	0.03	0.08	0.04
P09103	Protein disulfide-isomerase OS=Mus musculus GN=P4hb PE=1 SV=2	0.00	0.00	0.00	0.00
P09405	Nucleolin OS=Mus musculus GN=Ncl PE=1 SV=2	0.00	0.00	0.00	0.00
P09411	Phosphoglycerate kinase 1 OS=Mus musculus GN=Pgk1 PE=1 SV=4	1.00	0.08	1.00	0.03
P09602	Non-histone chromosomal protein HMG-17 OS=Mus musculus GN=Hmgn2 PE=1 SV=2	1.00	0.00	1.00	1.00
P10107	Annexin A1 OS=Mus musculus GN=Anxa1 PE=1 SV=2	1.00	1.00	0.00	0.00
P10126	Elongation factor 1-alpha 1 OS=Mus musculus GN=Eef1a1 PE=1 SV=3	0.00	0.00	0.00	0.00
P10639	Thioredoxin OS=Mus musculus GN=Txn PE=1 SV=3	0.02	0.00	0.00	0.00
P10649	Glutathione S-transferase Mu 1 OS=Mus musculus GN=Gstm1 PE=1 SV=2	1.00	0.00	0.00	0.00
P10922	Histone H1.0 OS=Mus musculus GN=H1f0 PE=2 SV=4	0.02	0.00	0.00	0.00
P11031	Activated RNA polymerase II transcriptional coactivator p15 OS=Mus musculus GN=Sub1 PE=1 SV=3	0.02	0.00	0.02	1.00
P11499	Heat shock protein HSP 90-beta OS=Mus musculus GN=Hsp90ab1 PE=1 SV=3	0.05	0.00	0.00	0.00
P11688	Integrin alpha-5 OS=Mus musculus GN=Itga5 PE=1 SV=3	1.00	0.04	0.02	1.00
P12970	60S ribosomal protein L7a OS=Mus musculus GN=Rpl7a PE=2 SV=2	1.00	0.00	0.02	0.00
P13020	Gelsolin OS=Mus musculus GN=Gsn PE=1 SV=3	0.05	0.00	0.00	0.00
P14069	Protein S100-A6 OS=Mus musculus GN=S100a6 PE=1 SV=3	0.04	0.02	0.02	0.03
P14115	60S ribosomal protein L27a OS=Mus musculus GN=Rpl27a PE=2 SV=5	1.00	0.02	1.00	0.02
P14131	40S ribosomal protein S16 OS=Mus musculus GN=Rps16 PE=2 SV=4	1.00	0.00	0.02	0.05
P14148	60S ribosomal protein L7 OS=Mus musculus GN=Rpl7 PE=2 SV=2	1.00	0.06	1.00	0.02
P14152	Malate dehydrogenase, cytoplasmic OS=Mus musculus GN=Mdh1 PE=1 SV=3	0.02	0.00	0.00	0.00
P14206	40S ribosomal protein SA OS=Mus musculus GN=Rpsa PE=1 SV=4	0.00	0.00	0.00	0.00
P14211	Calreticulin OS=Mus musculus GN=Calr PE=1 SV=1	0.00	0.00	0.00	0.00
P14431	H-2 class I histocompatibility antigen, Q9 alpha chain (Fragment) OS=Mus musculus GN=H2-Q9 PE=2 SV=1	1.00	0.04	1.00	1.00
P14733	Lamin-B1 OS=Mus musculus GN=Lmnb1 PE=1 SV=3	0.00	0.00	0.00	0.00
P14824	Annexin A6 OS=Mus musculus GN=Anxa6 PE=1 SV=3	0.03	0.00	0.00	0.00
P14869	60S acidic ribosomal protein P0 OS=Mus musculus GN=Rplp0 PE=1 SV=3	0.02	0.00	0.02	0.00
P15626	Glutathione S-transferase Mu 2 OS=Mus musculus GN=Gstm2 PE=1 SV=2	1.00	0.02	0.00	0.00
P15864	Histone H1.2 OS=Mus musculus GN=Hist1h1c PE=1 SV=2	0.00	0.00	0.00	0.00
P15947	Kallikrein-1 OS=Mus musculus GN=Klk1 PE=1 SV=3	1.00	1.00	0.02	1.00
P16045	Galectin-1 OS=Mus musculus GN=Lgals1 PE=1 SV=3	0.02	0.00	0.00	0.00
P16858	Glyceraldehyde-3-phosphate dehydrogenase OS=Mus musculus GN=Gapdh PE=1 SV=2	0.02	0.00	0.02	0.00
P17047	Lysosome-associated membrane glycoprotein 2 OS=Mus musculus GN=Lamp2 PE=2 SV=2	1.00	1.00	0.07	1.00
P17182	Alpha-enolase OS=Mus musculus GN=Eno1 PE=1 SV=3	0.00	0.00	0.00	0.00
P17742	Peptidyl-prolyl cis-trans isomerase A OS=Mus musculus GN=Ppia PE=1 SV=2	0.06	0.00	0.00	0.00
P17751	Triosephosphate isomerase OS=Mus musculus GN=Tpi1 PE=1 SV=3	0.00	0.00	0.00	0.00
P17897	Lysozyme C-1 OS=Mus musculus GN=Lyz1 PE=1 SV=1	1.00	1.00	1.00	0.03
P17918	Proliferating cell nuclear antigen OS=Mus musculus GN=Pcna PE=1 SV=2	0.02	0.00	0.02	1.00
P18242	Cathepsin D OS=Mus musculus GN=Ctsd PE=1 SV=1	1.00	1.00	1.00	0.02
P18572	Basigin OS=Mus musculus GN=Bsg PE=1 SV=2	1.00	1.00	0.03	0.04
P18760	Cofilin-1 OS=Mus musculus GN=Cfl1 PE=1 SV=3	0.00	0.00	0.02	0.00
P19001	Keratin, type I cytoskeletal 19 OS=Mus musculus GN=Krt19 PE=2 SV=1	1.00	0.06	1.00	1.00
P19157	Glutathione S-transferase P 1 OS=Mus musculus GN=Gstp1 PE=1 SV=2	1.00	0.02	1.00	0.00
P19253	60S ribosomal protein L13a OS=Mus musculus GN=Rpl13a PE=1 SV=4	1.00	0.00	0.02	0.00
P19324	Serpin H1 OS=Mus musculus GN=Serpinh1 PE=1 SV=3	0.00	0.00	0.00	0.00
P20029	78 kDa glucose-regulated protein OS=Mus musculus GN=Hspa5 PE=1 SV=3	0.00	0.00	0.00	0.00
P20065	Thymosin beta-4 OS=Mus musculus GN=Tmsb4x PE=1 SV=1	1.00	1.00	0.06	1.00
P20108	Thioredoxin-dependent peroxide reductase, mitochondrial OS=Mus musculus GN=Prdx3 PE=1 SV=1	1.00	1.00	0.04	1.00
P20152	Vimentin OS=Mus musculus GN=Vim PE=1 SV=3	0.02	0.00	0.00	0.00
P21981	Protein-glutamine gamma-glutamyltransferase 2 OS=Mus musculus GN=Tgm2 PE=1 SV=4	1.00	1.00	1.00	0.00
P23198	Chromobox protein homolog 3 OS=Mus musculus GN=Cbx3 PE=1 SV=2	1.00	0.00	0.00	0.02

P23492	Purine nucleoside phosphorylase OS=Mus musculus GN=Pnp PE=1 SV=2	1.00	0.07	0.03	0.03
P24270	Catalase OS=Mus musculus GN=Cat PE=1 SV=4	1.00	0.02	1.00	1.00
P24369	Peptidyl-prolyl cis-trans isomerase B OS=Mus musculus GN=Ppib PE=2 SV=2	0.03	0.00	0.00	0.00
P25206	DNA replication licensing factor MCM3 OS=Mus musculus GN=Mcm3 PE=1 SV=2	0.02	1.00	1.00	1.00
P25444	40S ribosomal protein S2 OS=Mus musculus GN=Rps2 PE=1 SV=3	1.00	0.02	0.00	0.00
P26039	Talin-1 OS=Mus musculus GN=Tln1 PE=1 SV=2	1.00	0.02	0.02	0.00
P26040	Ezrin OS=Mus musculus GN=Ezr PE=1 SV=3	1.00	0.02	0.02	0.00
P26041	Moesin OS=Mus musculus GN=Msn PE=1 SV=3	0.03	0.00	0.00	0.00
P26043	Radixin OS=Mus musculus GN=Rdx PE=1 SV=3	1.00	0.00	1.00	1.00
P26350	Prothymosin alpha OS=Mus musculus GN=Ptma PE=1 SV=2	0.00	0.00	0.00	0.00
P26443	Glutamate dehydrogenase 1, mitochondrial OS=Mus musculus GN=Glud1 PE=1 SV=1	1.00	1.00	0.02	1.00
P26638	Seryl-tRNA synthetase, cytoplasmic OS=Mus musculus GN=Sars PE=2 SV=3	1.00	0.06	1.00	1.00
P26645	Myristoylated alanine-rich C-kinase substrate OS=Mus musculus GN=Marcks PE=1 SV=2	0.00	0.00	0.00	0.00
P27546	Microtubule-associated protein 4 OS=Mus musculus GN=Map4 PE=1 SV=3	1.00	0.02	0.00	0.00
P27659	60S ribosomal protein L3 OS=Mus musculus GN=Rpl3 PE=2 SV=3	1.00	0.03	0.02	0.00
P27773	Protein disulfide-isomerase A3 OS=Mus musculus GN=Pdia3 PE=1 SV=2	0.00	0.00	0.00	0.00
P28352	DNA-(apurinic or apyrimidinic site) lyase OS=Mus musculus GN=Apex1 PE=1 SV=2	1.00	1.00	0.02	1.00
P28656	Nucleosome assembly protein 1-like 1 OS=Mus musculus GN=Nap1l1 PE=1 SV=2	0.04	1.00	1.00	1.00
P28660	Nck-associated protein 1 OS=Mus musculus GN=Nckap1 PE=2 SV=2	1.00	0.01	1.00	1.00
P28667	MARCKS-related protein OS=Mus musculus GN=Marcksl1 PE=1 SV=2	0.00	0.00	0.02	1.00
P28843	Dipeptidyl peptidase 4 OS=Mus musculus GN=Dpp4 PE=1 SV=3	1.00	1.00	1.00	0.02
P29341	Polyadenylate-binding protein 1 OS=Mus musculus GN=Pabpc1 PE=1 SV=2	0.04	1.00	0.03	1.00
P29758	Ornithine aminotransferase, mitochondrial OS=Mus musculus GN=Oat PE=1 SV=1	1.00	0.04	0.00	0.00
P30412	Peptidyl-prolyl cis-trans isomerase C OS=Mus musculus GN=Ppic PE=1 SV=1	1.00	0.05	1.00	0.02
P30416	Peptidyl-prolyl cis-trans isomerase FKBP4 OS=Mus musculus GN=Fkbp4 PE=1 SV=5	1.00	0.02	0.02	1.00
P30681	High mobility group protein B2 OS=Mus musculus GN=Hmgb2 PE=1 SV=3	0.04	0.00	0.00	0.07
P31001	Desmin OS=Mus musculus GN=Des PE=1 SV=3	1.00	0.02	0.00	0.00
P31786	Acyl-CoA-binding protein OS=Mus musculus GN=Dbi PE=1 SV=2	1.00	1.00	0.04	1.00
P34022	Ran-specific GTPase-activating protein OS=Mus musculus GN=Ranbp1 PE=1 SV=2	0.00	0.02	0.04	0.03
P34884	Macrophage migration inhibitory factor OS=Mus musculus GN=Mif PE=1 SV=2	0.04	0.02	1.00	1.00
P35278	Ras-related protein Rab-5C OS=Mus musculus GN=Rab5c PE=1 SV=2	1.00	1.00	1.00	0.00
P35486	Pyruvate dehydrogenase E1 component subunit alpha, somatic form, mitochondrial OS=Mus musculus GN=Pdha	1.00	0.02	1.00	1.00
P35492	Histidine ammonia-lyase OS=Mus musculus GN=Hal PE=1 SV=1	1.00	1.00	1.00	0.04
P35564	Calnexin OS=Mus musculus GN=Canx PE=1 SV=1	1.00	0.00	1.00	0.00
P35700	Peroxiredoxin-1 OS=Mus musculus GN=Prdx1 PE=1 SV=1	0.00	0.00	0.00	0.00
P35979	60S ribosomal protein L12 OS=Mus musculus GN=Rpl12 PE=1 SV=2	1.00	0.00	0.00	0.00
P35980	60S ribosomal protein L18 OS=Mus musculus GN=Rpl18 PE=2 SV=3	1.00	0.00	0.00	0.00
P37804	Transgelin OS=Mus musculus GN=Tagln PE=1 SV=3	1.00	0.02	0.00	0.00
P38060	Hydroxymethylglutaryl-CoA lyase, mitochondrial OS=Mus musculus GN=Hmgcl PE=1 SV=2	1.00	1.00	0.08	1.00
P40124	Adenylyl cyclase-associated protein 1 OS=Mus musculus GN=Cap1 PE=1 SV=4	1.00	0.06	0.02	0.02
P40142	Transketolase OS=Mus musculus GN=Tkt PE=1 SV=1	0.02	0.00	0.00	0.00
P42208	Septin-2 OS=Mus musculus GN=Sept2 PE=1 SV=2	1.00	0.05	1.00	0.00
P42932	T-complex protein 1 subunit theta OS=Mus musculus GN=Cct8 PE=1 SV=3	1.00	0.02	1.00	0.05
P43137	Lithostathine-1 OS=Mus musculus GN=Reg1 PE=2 SV=1	1.00	1.00	0.00	0.00
P43274	Histone H1.4 OS=Mus musculus GN=Hist1h1e PE=1 SV=2	1.00	0.00	0.02	0.00
P43275	Histone H1.1 OS=Mus musculus GN=Hist1h1a PE=2 SV=2	0.00	0.00	0.00	0.00
P43276	Histone H1.5 OS=Mus musculus GN=Hist1h1b PE=1 SV=2	0.00	0.00	0.00	0.00
P43277	Histone H1.3 OS=Mus musculus GN=Hist1h1d PE=1 SV=2	1.00	0.05	1.00	1.00
P45376	Aldose reductase OS=Mus musculus GN=Akr1b1 PE=1 SV=3	1.00	1.00	1.00	0.02
P45591	Cofilin-2 OS=Mus musculus GN=Cfl2 PE=1 SV=1	1.00	1.00	1.00	0.02
P45878	Peptidyl-prolyl cis-trans isomerase FKBP2 OS=Mus musculus GN=Fkbp2 PE=1 SV=1	1.00	0.02	1.00	1.00
P47738	Aldehyde dehydrogenase, mitochondrial OS=Mus musculus GN=Aldh2 PE=1 SV=1	1.00	0.00	0.00	0.00
P47740	Fatty aldehyde dehydrogenase OS=Mus musculus GN=Aldh3a2 PE=2 SV=2	1.00	1.00	1.00	0.02
P47753	F-actin-capping protein subunit alpha-1 OS=Mus musculus GN=Capza1 PE=1 SV=4	1.00	0.02	0.02	0.02
P47757	F-actin-capping protein subunit beta OS=Mus musculus GN=Capzb PE=1 SV=3	1.00	0.03	0.03	0.02
P47878	Insulin-like growth factor-binding protein 3 OS=Mus musculus GN=Igfbp3 PE=2 SV=1	1.00	0.03	1.00	1.00
P47911	60S ribosomal protein L6 OS=Mus musculus GN=Rpl6 PE=1 SV=3	1.00	1.00	0.02	0.00
P47915	60S ribosomal protein L29 OS=Mus musculus GN=Rpl29 PE=2 SV=2	1.00	0.02	1.00	1.00
P47955	60S acidic ribosomal protein P1 OS=Mus musculus GN=Rplp1 PE=1 SV=1	1.00	0.02	1.00	0.02
P47962	60S ribosomal protein L5 OS=Mus musculus GN=Rpl5 PE=1 SV=3	0.03	0.00	0.00	0.08
P47963	60S ribosomal protein L13 OS=Mus musculus GN=Rpl13 PE=2 SV=3	1.00	0.00	0.00	0.04
P47964	60S ribosomal protein L36 OS=Mus musculus GN=Rpl36 PE=2 SV=2	1.00	0.00	0.04	1.00
P48036	Annexin A5 OS=Mus musculus GN=Anxa5 PE=1 SV=1	0.02	0.00	0.00	0.00
P48678	Prelamin-A/C OS=Mus musculus GN=Lmna PE=1 SV=2	0.00	0.00	0.00	0.00
P48754	Breast cancer type 1 susceptibility protein homolog OS=Mus musculus GN=Brca1 PE=1 SV=2	1.00	0.04	1.00	1.00
P48758	Carbonyl reductase [NADPH] 1 OS=Mus musculus GN=Cbr1 PE=1 SV=3	1.00	0.02	1.00	1.00
P48771	Cytochrome c oxidase subunit 7A2, mitochondrial OS=Mus musculus GN=Cox7a2 PE=1 SV=2	1.00	0.02	1.00	1.00
P49183	Deoxyribonuclease-1 OS=Mus musculus GN=Dnase1 PE=2 SV=2	0.02	0.02	1.00	1.00
P49312	Heterogeneous nuclear ribonucleoprotein A1 OS=Mus musculus GN=Hnrnpa1 PE=1 SV=2	0.00	0.00	0.00	0.00
P49722	Proteasome subunit alpha type-2 OS=Mus musculus GN=Psma2 PE=1 SV=2	1.00	0.08	1.00	1.00
P49817	Caveolin-1 OS=Mus musculus GN=Cav1 PE=1 SV=1	1.00	1.00	0.05	0.00

P49919	Cyclin-dependent kinase inhibitor 1C OS=Mus musculus GN=Cdkn1c PE=2 SV=1	1.00	0.02	1.00	1.00
P50247	Adenosylhomocysteinase OS=Mus musculus GN=Ahcy PE=1 SV=3	0.05	0.00	0.00	0.00
P50543	Protein S100-A11 OS=Mus musculus GN=S100a11 PE=2 SV=1	1.00	0.02	1.00	0.02
P50580	Proliferation-associated protein 2G4 OS=Mus musculus GN=Pa2g4 PE=1 SV=3	0.00	0.00	0.00	1.00
P51150	Ras-related protein Rab-7a OS=Mus musculus GN=Rab7a PE=1 SV=2	1.00	0.07	0.02	0.00
P51174	Long-chain specific acyl-CoA dehydrogenase, mitochondrial OS=Mus musculus GN=Acadl PE=2 SV=2	1.00	1.00	0.02	0.02
P51410	60S ribosomal protein L9 OS=Mus musculus GN=Rpl9 PE=2 SV=2	1.00	1.00	1.00	0.00
P51660	Peroxisomal multifunctional enzyme type 2 OS=Mus musculus GN=Hsd17b4 PE=1 SV=3	1.00	1.00	0.03	1.00
P51859	Hepatoma-derived growth factor OS=Mus musculus GN=Hdgf PE=1 SV=2	1.00	0.00	0.02	0.04
P52480	Pyruvate kinase isozymes M1/M2 OS=Mus musculus GN=Pkm2 PE=1 SV=4	0.00	0.00	0.00	0.00
P52793	Ephrin-A1 OS=Mus musculus GN=Efna1 PE=2 SV=1	1.00	0.07	1.00	1.00
P52927	High mobility group protein HMGI-C OS=Mus musculus GN=Hmga2 PE=1 SV=1	0.02	0.05	1.00	1.00
P53026	60S ribosomal protein L10a OS=Mus musculus GN=Rpl10a PE=1 SV=3	1.00	0.03	0.02	0.05
P54071	Isocitrate dehydrogenase [NADP], mitochondrial OS=Mus musculus GN=Idh2 PE=1 SV=3	1.00	0.00	0.00	0.00
P54227	Stathmin OS=Mus musculus GN=Stmn1 PE=1 SV=2	0.05	0.00	1.00	1.00
P56395	Cytochrome b5 OS=Mus musculus GN=Cyb5a PE=1 SV=2	1.00	1.00	1.00	0.02
P56480	ATP synthase subunit beta, mitochondrial OS=Mus musculus GN=Atp5b PE=1 SV=2	0.00	0.00	0.00	0.00
P56501	Mitochondrial uncoupling protein 3 OS=Mus musculus GN=Ucp3 PE=2 SV=1	0.05	0.03	1.00	0.08
P56959	RNA-binding protein FUS OS=Mus musculus GN=Fus PE=2 SV=1	0.02	0.00	0.00	0.00
P57759	Endoplasmic reticulum resident protein 29 OS=Mus musculus GN=Erp29 PE=1 SV=2	1.00	0.05	1.00	1.00
P57776	Elongation factor 1-delta OS=Mus musculus GN=Eef1d PE=1 SV=3	1.00	0.02	0.02	0.00
P57780	Alpha-actinin-4 OS=Mus musculus GN=Actn4 PE=1 SV=1	1.00	0.05	0.02	0.00
P58252	Elongation factor 2 OS=Mus musculus GN=Eef2 PE=1 SV=2	0.02	0.00	0.00	0.00
P58771	Tropomyosin alpha-1 chain OS=Mus musculus GN=Tpm1 PE=1 SV=1	0.02	0.00	0.00	0.00
P58871	182 kDa tankyrase-1-binding protein OS=Mus musculus GN=Tnks1bp1 PE=1 SV=2	1.00	1.00	1.00	0.06
P59158	Solute carrier family 12 member 3 OS=Mus musculus GN=Slc12a3 PE=2 SV=1	1.00	0.04	1.00	1.00
P60122	RuvB-like 1 OS=Mus musculus GN=Ruvbl1 PE=1 SV=1	1.00	1.00	0.02	1.00
P60335	Poly(rC)-binding protein 1 OS=Mus musculus GN=Pcbp1 PE=1 SV=1	1.00	0.02	0.02	0.02
P60766	Cell division control protein 42 homolog OS=Mus musculus GN=Cdc42 PE=1 SV=2	1.00	0.00	0.02	1.00
P60824	Cold-inducible RNA-binding protein OS=Mus musculus GN=Cirbp PE=1 SV=1	1.00	1.00	1.00	0.05
P60843	Eukaryotic initiation factor 4A-I OS=Mus musculus GN=Eif4a1 PE=2 SV=1	0.03	0.00	0.02	0.00
P60867	40S ribosomal protein S20 OS=Mus musculus GN=Rps20 PE=1 SV=1	0.05	0.00	0.00	0.00
P61089	Ubiquitin-conjugating enzyme E2 N OS=Mus musculus GN=Ube2n PE=1 SV=1	0.02	0.00	0.02	0.00
P61168	D(2) dopamine receptor OS=Mus musculus GN=Drd2 PE=2 SV=2	0.04	0.03	1.00	1.00
P61255	60S ribosomal protein L26 OS=Mus musculus GN=Rpl26 PE=2 SV=1	0.00	0.00	0.05	1.00
P61358	60S ribosomal protein L27 OS=Mus musculus GN=Rpl27 PE=2 SV=2	1.00	0.04	0.02	0.02
P61750	ADP-ribosylation factor 4 OS=Mus musculus GN=Arf4 PE=2 SV=2	0.02	0.02	0.02	0.02
P61804	Dolichyl-diphosphooligosaccharide--protein glycosyltransferase subunit DAD1 OS=Mus musculus GN=Dad1 PE=	1.00	0.03	1.00	0.00
P61957	Small ubiquitin-related modifier 2 OS=Mus musculus GN=Sumo2 PE=2 SV=1	1.00	0.02	0.00	1.00
P61979	Heterogeneous nuclear ribonucleoprotein K OS=Mus musculus GN=Hnrnpk PE=1 SV=1	0.00	0.00	0.00	0.00
P61982	14-3-3 protein gamma OS=Mus musculus GN=Ywhag PE=1 SV=2	1.00	0.02	0.00	0.00
P62071	Ras-related protein R-Ras2 OS=Mus musculus GN=Rras2 PE=1 SV=1	1.00	1.00	1.00	0.02
P62082	40S ribosomal protein S7 OS=Mus musculus GN=Rps7 PE=2 SV=1	1.00	0.00	1.00	0.00
P62137	Serine/threonine-protein phosphatase PP1-alpha catalytic subunit OS=Mus musculus GN=Ppp1ca PE=1 SV=1	1.00	0.02	1.00	1.00
P62141	Serine/threonine-protein phosphatase PP1-beta catalytic subunit OS=Mus musculus GN=Ppp1cb PE=1 SV=3	1.00	0.02	1.00	1.00
P62204	Calmodulin OS=Mus musculus GN=Calm1 PE=1 SV=2	1.00	0.00	0.02	0.00
P62242	40S ribosomal protein S8 OS=Mus musculus GN=Rps8 PE=1 SV=2	0.02	0.00	0.00	0.00
P62245	40S ribosomal protein S15a OS=Mus musculus GN=Rps15a PE=2 SV=2	1.00	0.02	0.03	0.00
P62259	14-3-3 protein epsilon OS=Mus musculus GN=Ywhae PE=1 SV=1	0.00	0.00	0.00	0.00
P62264	40S ribosomal protein S14 OS=Mus musculus GN=Rps14 PE=2 SV=3	0.02	0.00	0.00	0.02
P62270	40S ribosomal protein S18 OS=Mus musculus GN=Rps18 PE=2 SV=3	0.00	0.00	0.00	0.07
P62281	40S ribosomal protein S11 OS=Mus musculus GN=Rps11 PE=2 SV=3	1.00	1.00	0.07	1.00
P62301	40S ribosomal protein S13 OS=Mus musculus GN=Rps13 PE=1 SV=2	1.00	0.05	1.00	1.00
P62305	Small nuclear ribonucleoprotein E OS=Mus musculus GN=Snrpe PE=2 SV=1	1.00	0.00	1.00	0.02
P62307	Small nuclear ribonucleoprotein F OS=Mus musculus GN=Snrpf PE=2 SV=1	1.00	0.00	0.02	1.00
P62309	Small nuclear ribonucleoprotein G OS=Mus musculus GN=Snrpg PE=1 SV=1	0.03	1.00	1.00	1.00
P62315	Small nuclear ribonucleoprotein Sm D1 OS=Mus musculus GN=Snrpd1 PE=2 SV=1	1.00	0.02	1.00	0.02
P62317	Small nuclear ribonucleoprotein Sm D2 OS=Mus musculus GN=Snrpd2 PE=2 SV=1	1.00	0.02	0.00	1.00
P62320	Small nuclear ribonucleoprotein Sm D3 OS=Mus musculus GN=Snrpd3 PE=1 SV=1	1.00	0.02	1.00	1.00
P62492	Ras-related protein Rab-11A OS=Mus musculus GN=Rab11a PE=1 SV=3	1.00	0.00	0.00	0.00
P62500	TSC22 domain family protein 1 OS=Mus musculus GN=Tsc22d1 PE=2 SV=2	1.00	0.02	0.02	1.00
P62702	40S ribosomal protein S4, X isoform OS=Mus musculus GN=Rps4x PE=2 SV=2	0.02	0.00	0.04	0.00
P62717	60S ribosomal protein L18a OS=Mus musculus GN=Rpl18a PE=1 SV=1	1.00	1.00	0.02	1.00
P62751	60S ribosomal protein L23a OS=Mus musculus GN=Rpl23a PE=1 SV=1	0.02	0.00	0.00	0.00
P62754	40S ribosomal protein S6 OS=Mus musculus GN=Rps6 PE=1 SV=1	1.00	0.00	1.00	1.00
P62806	Histone H4 OS=Mus musculus GN=Hist1h4a PE=1 SV=2	0.00	0.00	0.00	0.00
P62821	Ras-related protein Rab-1A OS=Mus musculus GN=Rab1A PE=1 SV=3	1.00	1.00	1.00	0.02
P62827	GTP-binding nuclear protein Ran OS=Mus musculus GN=Ran PE=1 SV=3	0.00	0.00	0.00	0.00
P62830	60S ribosomal protein L23 OS=Mus musculus GN=Rpl23 PE=1 SV=1	1.00	1.00	1.00	0.00
P62843	40S ribosomal protein S15 OS=Mus musculus GN=Rps15 PE=2 SV=2	1.00	0.02	0.02	0.05
P62849	40S ribosomal protein S24 OS=Mus musculus GN=Rps24 PE=1 SV=1	0.08	1.00	1.00	0.02

P62852	40S ribosomal protein S25 OS=Mus musculus GN=Rps25 PE=2 SV=1	1.00	0.00	0.02	0.00
P62855	40S ribosomal protein S26 OS=Mus musculus GN=Rps26 PE=2 SV=3	1.00	0.00	0.02	0.02
P62858	40S ribosomal protein S28 OS=Mus musculus GN=Rps28 PE=2 SV=1	1.00	0.00	0.02	0.02
P62869	Transcription elongation factor B polypeptide 2 OS=Mus musculus GN=Tceb2 PE=1 SV=1	1.00	0.02	1.00	1.00
P62880	Guanine nucleotide-binding protein G(I)/G(S)/G(T) subunit beta-2 OS=Mus musculus GN=Gnb2 PE=1 SV=3	1.00	0.05	1.00	0.02
P62889	60S ribosomal protein L30 OS=Mus musculus GN=Rpl30 PE=2 SV=2	1.00	0.05	1.00	0.00
P62900	60S ribosomal protein L31 OS=Mus musculus GN=Rpl31 PE=2 SV=1	0.07	0.02	0.02	0.03
P62908	40S ribosomal protein S3 OS=Mus musculus GN=Rps3 PE=1 SV=1	0.02	0.05	0.02	0.00
P62911	60S ribosomal protein L32 OS=Mus musculus GN=Rpl32 PE=2 SV=2	1.00	1.00	0.02	0.02
P62918	60S ribosomal protein L8 OS=Mus musculus GN=Rpl8 PE=2 SV=2	0.00	0.00	0.00	0.00
P62960	Nuclease-sensitive element-binding protein 1 OS=Mus musculus GN=Ybx1 PE=1 SV=3	1.00	0.00	0.02	0.00
P62962	Profilin-1 OS=Mus musculus GN=Pfn1 PE=1 SV=2	0.08	0.00	0.03	0.00
P62965	Cellular retinoic acid-binding protein 1 OS=Mus musculus GN=Crabp1 PE=1 SV=2	1.00	0.08	1.00	1.00
P62984	Ubiquitin-60S ribosomal protein L40 OS=Mus musculus GN=Uba52 PE=1 SV=2	0.00	0.00	0.00	0.00
P62996	Transformer-2 protein homolog beta OS=Mus musculus GN=Tra2b PE=1 SV=1	1.00	1.00	1.00	0.02
P63005	Platelet-activating factor acetylhydrolase IB subunit alpha OS=Mus musculus GN=Pafah1b1 PE=1 SV=2	1.00	1.00	0.02	1.00
P63017	Heat shock cognate 71 kDa protein OS=Mus musculus GN=Hspa8 PE=1 SV=1	0.00	0.00	0.00	0.00
P63028	Translationally-controlled tumor protein OS=Mus musculus GN=Tpt1 PE=1 SV=1	1.00	1.00	1.00	0.02
P63038	60 kDa heat shock protein, mitochondrial OS=Mus musculus GN=Hspd1 PE=1 SV=1	0.00	0.00	0.00	0.00
P63085	Mitogen-activated protein kinase 1 OS=Mus musculus GN=Mapk1 PE=1 SV=3	1.00	0.02	1.00	0.02
P63101	14-3-3 protein zeta/delta OS=Mus musculus GN=Ywhaz PE=1 SV=1	0.00	0.00	0.00	0.00
P63158	High mobility group protein B1 OS=Mus musculus GN=Hmgb1 PE=1 SV=2	0.00	0.00	0.00	0.00
P63163	Small nuclear ribonucleoprotein-associated protein N OS=Mus musculus GN=Snrpn PE=2 SV=1	0.05	1.00	1.00	1.00
P63242	Eukaryotic translation initiation factor 5A-1 OS=Mus musculus GN=Eif5a PE=1 SV=2	0.02	0.00	0.04	0.00
P63254	Cysteine-rich protein 1 OS=Mus musculus GN=Crip1 PE=2 SV=2	1.00	0.02	0.02	0.02
P63260	Actin, cytoplasmic 2 OS=Mus musculus GN=Actg1 PE=1 SV=1	0.00	0.00	0.00	0.00
P63276	40S ribosomal protein S17 OS=Mus musculus GN=Rps17 PE=1 SV=2	1.00	0.02	1.00	0.02
P63323	40S ribosomal protein S12 OS=Mus musculus GN=Rps12 PE=1 SV=2	1.00	0.02	0.02	0.03
P63325	40S ribosomal protein S10 OS=Mus musculus GN=Rps10 PE=1 SV=1	0.02	0.07	0.02	1.00
P67778	Prohibitin OS=Mus musculus GN=Phb PE=1 SV=1	1.00	1.00	0.02	1.00
P67871	Casein kinase II subunit beta OS=Mus musculus GN=Csnk2b PE=1 SV=1	1.00	1.00	0.06	1.00
P67984	60S ribosomal protein L22 OS=Mus musculus GN=Rpl22 PE=2 SV=2	1.00	0.00	0.00	0.00
P68037	Ubiquitin-conjugating enzyme E2 L3 OS=Mus musculus GN=Ube2l3 PE=2 SV=1	1.00	0.04	0.06	1.00
P68040	Guanine nucleotide-binding protein subunit beta-2-like 1 OS=Mus musculus GN=Gnb2l1 PE=1 SV=3	1.00	1.00	0.02	0.05
P68134	Actin, alpha skeletal muscle OS=Mus musculus GN=Acta1 PE=1 SV=1	0.00	0.00	0.00	0.00
P68254	14-3-3 protein theta OS=Mus musculus GN=Ywhaq PE=1 SV=1	0.00	0.00	0.02	0.00
P68369	Tubulin alpha-1A chain OS=Mus musculus GN=Tuba1a PE=1 SV=1	0.07	0.00	0.02	0.00
P68372	Tubulin beta-2C chain OS=Mus musculus GN=Tubb2c PE=1 SV=1	1.00	1.00	1.00	0.00
P68433	Histone H3.1 OS=Mus musculus GN=Hist1h3a PE=1 SV=2	0.00	0.00	0.03	0.02
P68510	14-3-3 protein eta OS=Mus musculus GN=Ywhah PE=1 SV=2	0.03	0.00	0.00	0.00
P70121	Zinc fingers and homeoboxes protein 1 OS=Mus musculus GN=Zhx1 PE=1 SV=2	1.00	0.07	1.00	1.00
P70168	Importin subunit beta-1 OS=Mus musculus GN=Kpnb1 PE=1 SV=2	1.00	1.00	0.04	0.00
P70195	Proteasome subunit beta type-7 OS=Mus musculus GN=Psmb7 PE=1 SV=1	1.00	0.00	0.03	1.00
P70296	Phosphatidylethanolamine-binding protein 1 OS=Mus musculus GN=Pebp1 PE=1 SV=3	1.00	0.04	1.00	0.00
P70333	Heterogeneous nuclear ribonucleoprotein H2 OS=Mus musculus GN=Hnrnph2 PE=1 SV=1	1.00	0.00	0.02	0.00
P70349	Histidine triad nucleotide-binding protein 1 OS=Mus musculus GN=Hint1 PE=1 SV=3	1.00	1.00	0.02	0.02
P70372	ELAV-like protein 1 OS=Mus musculus GN=Elavl1 PE=1 SV=2	1.00	0.02	0.02	1.00
P70441	Na(+)/H(+) exchange regulatory cofactor NHE-RF1 OS=Mus musculus GN=Slc9a3r1 PE=1 SV=3	1.00	1.00	0.02	0.00
P80313	T-complex protein 1 subunit eta OS=Mus musculus GN=Cct7 PE=1 SV=1	1.00	1.00	0.02	1.00
P80315	T-complex protein 1 subunit delta OS=Mus musculus GN=Cct4 PE=1 SV=3	1.00	0.06	1.00	0.04
P80316	T-complex protein 1 subunit epsilon OS=Mus musculus GN=Cct5 PE=1 SV=1	1.00	1.00	1.00	0.02
P83917	Chromobox protein homolog 1 OS=Mus musculus GN=Cbx1 PE=1 SV=1	1.00	0.02	0.04	0.02
P84078	ADP-ribosylation factor 1 OS=Mus musculus GN=Arf1 PE=1 SV=2	1.00	0.00	0.04	0.00
P84099	60S ribosomal protein L19 OS=Mus musculus GN=Rpl19 PE=1 SV=1	0.02	0.02	0.02	0.00
P84104	Serine/arginine-rich splicing factor 3 OS=Mus musculus GN=Srsf3 PE=2 SV=1	0.02	0.02	1.00	0.02
P84228	Histone H3.2 OS=Mus musculus GN=Hist1h3b PE=1 SV=2	0.02	0.02	1.00	0.02
P84244	Histone H3.3 OS=Mus musculus GN=H3f3a PE=1 SV=2	0.02	0.02	1.00	0.02
P97300	Neuroplastin OS=Mus musculus GN=Nptn PE=1 SV=3	1.00	1.00	0.07	0.08
P97310	DNA replication licensing factor MCM2 OS=Mus musculus GN=Mcm2 PE=1 SV=3	1.00	1.00	0.02	1.00
P97315	Cysteine and glycine-rich protein 1 OS=Mus musculus GN=Csrp1 PE=1 SV=3	1.00	1.00	0.05	1.00
P97350	Plakophilin-1 OS=Mus musculus GN=Pkp1 PE=1 SV=1	1.00	1.00	1.00	0.02
P97351	40S ribosomal protein S3a OS=Mus musculus GN=Rps3a PE=1 SV=3	1.00	0.00	0.00	0.02
P97352	Protein S100-A13 OS=Mus musculus GN=S100a13 PE=1 SV=1	1.00	1.00	0.02	0.02
P97371	Proteasome activator complex subunit 1 OS=Mus musculus GN=Psme1 PE=2 SV=2	0.03	1.00	0.02	0.02
P97372	Proteasome activator complex subunit 2 OS=Mus musculus GN=Psme2 PE=2 SV=4	1.00	1.00	1.00	0.05
P97429	Annexin A4 OS=Mus musculus GN=Anxa4 PE=2 SV=4	1.00	0.04	0.00	0.00
P97447	Four and a half LIM domains protein 1 OS=Mus musculus GN=Fhl1 PE=2 SV=3	1.00	1.00	0.02	0.02
P97449	Aminopeptidase N OS=Mus musculus GN=Anpep PE=1 SV=4	1.00	1.00	0.03	0.00
P97450	ATP synthase-coupling factor 6, mitochondrial OS=Mus musculus GN=Atp5j PE=1 SV=1	1.00	0.04	1.00	1.00
P97497	Sex hormone-binding globulin OS=Mus musculus GN=Shbg PE=2 SV=1	1.00	0.07	1.00	1.00
P97807	Fumarate hydratase, mitochondrial OS=Mus musculus GN=Fh PE=1 SV=3	1.00	0.02	1.00	0.00

P97855	Ras GTPase-activating protein-binding protein 1 OS=Mus musculus GN=G3bp1 PE=1 SV=1	1.00	1.00	1.00	0.00
P99024	Tubulin beta-5 chain OS=Mus musculus GN=Tubb5 PE=1 SV=1	1.00	0.02	0.00	0.00
P99026	Proteasome subunit beta type-4 OS=Mus musculus GN=Psmb4 PE=1 SV=1	1.00	0.00	1.00	1.00
P99027	60S acidic ribosomal protein P2 OS=Mus musculus GN=Rplp2 PE=1 SV=3	0.00	0.00	0.02	0.00
P99029	Peroxiredoxin-5, mitochondrial OS=Mus musculus GN=Prdx5 PE=1 SV=2	1.00	0.02	1.00	0.00
Q00519	Xanthine dehydrogenase/oxidase OS=Mus musculus GN=Xdh PE=1 SV=5	1.00	1.00	1.00	0.02
Q00915	Retinol-binding protein 1 OS=Mus musculus GN=Rbp1 PE=2 SV=2	1.00	0.02	0.02	0.00
Q00PI9	Heterogeneous nuclear ribonucleoprotein U-like protein 2 OS=Mus musculus GN=Hnrnpul2 PE=1 SV=1	1.00	1.00	0.02	1.00
Q01730	Ras suppressor protein 1 OS=Mus musculus GN=Rsu1 PE=2 SV=3	1.00	1.00	1.00	0.02
Q01768	Nucleoside diphosphate kinase B OS=Mus musculus GN=Nme2 PE=1 SV=1	0.02	0.00	0.00	0.00
Q01853	Transitional endoplasmic reticulum ATPase OS=Mus musculus GN=Vcp PE=1 SV=4	0.03	0.00	1.00	0.00
Q02053	Ubiquitin-like modifier-activating enzyme 1 OS=Mus musculus GN=Uba1 PE=1 SV=1	0.08	0.00	0.00	0.00
Q02257	Junction plakoglobin OS=Mus musculus GN=Jup PE=1 SV=3	1.00	1.00	1.00	0.00
Q03265	ATP synthase subunit alpha, mitochondrial OS=Mus musculus GN=Atp5a1 PE=1 SV=1	0.07	0.00	0.02	0.00
Q04447	Creatine kinase B-type OS=Mus musculus GN=Ckb PE=1 SV=1	1.00	1.00	1.00	0.00
Q04857	Collagen alpha-1(VI) chain OS=Mus musculus GN=Col6a1 PE=2 SV=1	1.00	1.00	0.02	0.04
Q05144	Ras-related C3 botulinum toxin substrate 2 OS=Mus musculus GN=Rac2 PE=2 SV=1	1.00	1.00	0.02	0.02
Q05186	Reticulocalbin-1 OS=Mus musculus GN=Rcn1 PE=1 SV=1	0.02	0.00	0.02	0.00
Q05816	Fatty acid-binding protein, epidermal OS=Mus musculus GN=Fabp5 PE=1 SV=3	0.02	0.00	0.00	0.00
Q06185	ATP synthase subunit e, mitochondrial OS=Mus musculus GN=Atp5i PE=1 SV=2	1.00	0.02	0.02	1.00
Q07113	Cation-independent mannose-6-phosphate receptor OS=Mus musculus GN=Igf2r PE=1 SV=1	0.02	0.00	0.00	1.00
Q07475	Neuronal protein 3.1 OS=Mus musculus GN=P311 PE=2 SV=1	1.00	1.00	0.05	1.00
Q08189	Protein-glutamine gamma-glutamyltransferase E OS=Mus musculus GN=Tgm3 PE=1 SV=2	1.00	1.00	1.00	0.03
Q09163	Protein delta homolog 1 OS=Mus musculus GN=Dlk1 PE=1 SV=1	1.00	0.04	1.00	1.00
Q0VBK2	Keratin, type II cytoskeletal 80 OS=Mus musculus GN=Krt80 PE=2 SV=1	1.00	1.00	1.00	0.00
Q2VIS4	Filaggrin-2 OS=Mus musculus GN=Flg2 PE=1 SV=2	1.00	1.00	1.00	0.02
Q3TEA8	Heterochromatin protein 1-binding protein 3 OS=Mus musculus GN=Hp1bp3 PE=1 SV=1	1.00	1.00	1.00	0.04
Q3THE2	Myosin regulatory light chain 12B OS=Mus musculus GN=Myl12b PE=1 SV=2	1.00	0.00	0.00	0.00
Q3THW5	Histone H2A.V OS=Mus musculus GN=H2afv PE=1 SV=3	0.02	0.05	1.00	0.00
Q3TJ91	Lethal(2) giant larvae protein homolog 2 OS=Mus musculus GN=Llgl2 PE=2 SV=2	1.00	0.04	1.00	1.00
Q3TML0	Uncharacterized protein OS=Mus musculus GN=Pdia6 PE=2 SV=1	0.02	0.00	0.00	0.00
Q3TWW8	Splicing factor, arginine/serine-rich 6 OS=Mus musculus GN=Srsf6 PE=2 SV=1	1.00	0.02	0.02	1.00
Q3TYA6	RIKEN cDNA 4930548G07 OS=Mus musculus GN=Mphosph8 PE=2 SV=1	0.04	1.00	1.00	1.00
Q3U0V1	Far upstream element-binding protein 2 OS=Mus musculus GN=Khsrp PE=1 SV=2	1.00	0.00	0.00	0.03
Q3U1J4	DNA damage-binding protein 1 OS=Mus musculus GN=Ddb1 PE=1 SV=2	1.00	1.00	0.08	1.00
Q3U7R1	Extended synaptotagmin-1 OS=Mus musculus GN=Esyt1 PE=2 SV=2	1.00	1.00	1.00	0.03
Q3UHD9	Arf-GAP with GTPase, ANK repeat and PH domain-containing protein 2 OS=Mus musculus GN=Agap2 PE=1 SV	1.00	0.04	1.00	1.00
Q3UHX2	28 kDa heat- and acid-stable phosphoprotein OS=Mus musculus GN=Pdap1 PE=1 SV=1	1.00	1.00	1.00	0.05
Q3UJB0	Splicing factor 3b, subunit 2 OS=Mus musculus GN=Sf3b2 PE=2 SV=1	0.04	1.00	1.00	1.00
Q3USB7	Inactive phospholipase C-like protein 1 OS=Mus musculus GN=Plcl1 PE=1 SV=3	1.00	0.06	1.00	1.00
Q3UW53	Protein Niban OS=Mus musculus GN=Fam129a PE=1 SV=2	1.00	1.00	1.00	0.00
Q3V0P4	Gene model 1661 OS=Mus musculus GN=Gm1661 PE=2 SV=1	1.00	1.00	0.08	1.00
Q3V0Q1	Dynein heavy chain 12, axonemal OS=Mus musculus GN=Dnah12 PE=1 SV=2	1.00	1.00	1.00	0.05
Q3V1T4	Prolyl 3-hydroxylase 1 OS=Mus musculus GN=Lepre1 PE=2 SV=2	1.00	1.00	1.00	0.02
Q4KL66	Pregnancy-specific glycoprotein 28 OS=Mus musculus GN=Psg28 PE=2 SV=1	0.01	1.00	1.00	1.00
Q4U4S6	Xin actin-binding repeat-containing protein 2 OS=Mus musculus GN=Xirp2 PE=1 SV=1	1.00	1.00	1.00	0.07
Q4V9X0	Ppp3ca protein OS=Mus musculus GN=Ppp3ca PE=2 SV=1	1.00	0.07	1.00	1.00
Q501J6	Probable ATP-dependent RNA helicase DDX17 OS=Mus musculus GN=Ddx17 PE=2 SV=1	0.02	0.02	1.00	0.00
Q504N0	Carboxypeptidase A2 OS=Mus musculus GN=Cpa2 PE=2 SV=1	1.00	0.00	0.03	1.00
Q52KH6	Novel protein OS=Mus musculus GN=DXBay18 PE=2 SV=1	1.00	1.00	1.00	0.03
Q58E70	Tpm3 protein OS=Mus musculus GN=Tpm3 PE=2 SV=1	1.00	0.00	0.00	0.00
Q5BKQ4	Pancreatic lipase-related protein 1 OS=Mus musculus GN=Pnliprp1 PE=2 SV=2	0.02	0.00	0.00	0.00
Q5DTT4	Retrotransposon gag domain-containing protein 4 OS=Mus musculus GN=Rgag4 PE=2 SV=2	1.00	0.01	1.00	1.00
Q5F4T0	Transient receptor potential cation channel, subfamily M, member 3 OS=Mus musculus GN=Trpm3 PE=2 SV=1	1.00	0.02	1.00	1.00
Q5XJY5	Coatomer subunit delta OS=Mus musculus GN=Arcn1 PE=2 SV=2	1.00	1.00	1.00	0.02
Q60605	Myosin light polypeptide 6 OS=Mus musculus GN=Myl6 PE=1 SV=3	1.00	0.02	0.00	0.00
Q60662	A-kinase anchor protein 4 OS=Mus musculus GN=Akap4 PE=1 SV=1	0.06	0.02	1.00	0.04
Q60668	Heterogeneous nuclear ribonucleoprotein D0 OS=Mus musculus GN=Hnrnpd PE=1 SV=2	1.00	0.00	0.02	0.00
Q60692	Proteasome subunit beta type-6 OS=Mus musculus GN=Psmb6 PE=1 SV=3	1.00	0.00	0.02	0.02
Q60715	Prolyl 4-hydroxylase subunit alpha-1 OS=Mus musculus GN=P4ha1 PE=2 SV=2	1.00	1.00	1.00	0.00
Q60716	Prolyl 4-hydroxylase subunit alpha-2 OS=Mus musculus GN=P4ha2 PE=2 SV=1	1.00	0.02	0.02	1.00
Q60739	BAG family molecular chaperone regulator 1 OS=Mus musculus GN=Bag1 PE=1 SV=3	1.00	0.07	0.02	0.03
Q60751	Insulin-like growth factor 1 receptor OS=Mus musculus GN=Igf1r PE=1 SV=3	1.00	0.01	1.00	1.00
Q60817	Nascent polypeptide-associated complex subunit alpha OS=Mus musculus GN=Naca PE=1 SV=1	1.00	0.00	1.00	0.02
Q60854	Serpin B6 OS=Mus musculus GN=Serpinb6 PE=2 SV=1	1.00	1.00	1.00	0.00
Q60864	Stress-induced-phosphoprotein 1 OS=Mus musculus GN=Stip1 PE=1 SV=1	1.00	0.02	0.04	1.00
Q60865	Caprin-1 OS=Mus musculus GN=Caprin1 PE=1 SV=2	1.00	1.00	1.00	0.07
Q60870	Receptor expression-enhancing protein 5 OS=Mus musculus GN=Reep5 PE=1 SV=1	1.00	1.00	0.02	1.00
Q60931	Voltage-dependent anion-selective channel protein 3 OS=Mus musculus GN=Vdac3 PE=1 SV=1	1.00	1.00	1.00	0.07
Q60932	Voltage-dependent anion-selective channel protein 1 OS=Mus musculus GN=Vdac1 PE=1 SV=3	1.00	0.00	0.00	0.00
Q60997	Deleted in malignant brain tumors 1 protein OS=Mus musculus GN=Dmbt1 PE=1 SV=2	1.00	1.00	0.00	1.00

Q61029	Lamina-associated polypeptide 2, isoforms beta/delta/epsilon/gamma OS=Mus musculus GN=Tmpo PE=1 SV=4	1.00	0.02	0.00	0.00
Q61035	Histidyl-tRNA synthetase, cytoplasmic OS=Mus musculus GN=Hars PE=2 SV=2	1.00	0.02	0.02	1.00
Q61171	Peroxiredoxin-2 OS=Mus musculus GN=Prdx2 PE=1 SV=3	0.02	0.00	0.02	0.00
Q61207	Sulfated glycoprotein 1 OS=Mus musculus GN=Psap PE=1 SV=2	1.00	0.02	0.08	1.00
Q61409	cGMP-inhibited 3',5'-cyclic phosphodiesterase B OS=Mus musculus GN=Pde3b PE=1 SV=2	1.00	0.02	1.00	1.00
Q61425	Hydroxyacyl-coenzyme A dehydrogenase, mitochondrial OS=Mus musculus GN=Hadh PE=1 SV=2	1.00	0.00	1.00	1.00
Q61545	RNA-binding protein EWS OS=Mus musculus GN=Ewsr1 PE=1 SV=2	1.00	0.00	1.00	1.00
Q61553	Fascin OS=Mus musculus GN=Fscn1 PE=1 SV=4	1.00	0.00	0.00	0.00
Q61598	Rab GDP dissociation inhibitor beta OS=Mus musculus GN=Gdi2 PE=1 SV=1	1.00	1.00	1.00	0.02
Q61599	Rho GDP-dissociation inhibitor 2 OS=Mus musculus GN=Arhgdib PE=1 SV=3	1.00	1.00	1.00	0.02
Q61753	D-3-phosphoglycerate dehydrogenase OS=Mus musculus GN=Phgdh PE=1 SV=3	1.00	0.03	0.00	1.00
Q61792	LIM and SH3 domain protein 1 OS=Mus musculus GN=Lasp1 PE=1 SV=1	1.00	1.00	1.00	0.02
Q61879	Myosin-10 OS=Mus musculus GN=Myh10 PE=1 SV=2	1.00	1.00	1.00	0.02
Q61937	Nucleophosmin OS=Mus musculus GN=Npm1 PE=1 SV=1	0.00	0.00	0.00	0.00
Q61990	Poly(rC)-binding protein 2 OS=Mus musculus GN=Pcbp2 PE=1 SV=1	1.00	1.00	1.00	0.00
Q62048	Astrocytic phosphoprotein PEA-15 OS=Mus musculus GN=Pea15 PE=1 SV=1	1.00	0.02	0.02	0.02
Q62093	Serine/arginine-rich splicing factor 2 OS=Mus musculus GN=Srsf2 PE=1 SV=4	1.00	0.02	1.00	0.02
Q62148	Retinal dehydrogenase 2 OS=Mus musculus GN=Aldh1a2 PE=1 SV=2	1.00	0.03	1.00	0.02
Q62167	ATP-dependent RNA helicase DDX3X OS=Mus musculus GN=Ddx3x PE=1 SV=3	0.08	1.00	0.05	1.00
Q62188	Dihydropyrimidinase-related protein 3 OS=Mus musculus GN=Dpysl3 PE=1 SV=1	1.00	0.03	0.02	0.00
Q62189	U1 small nuclear ribonucleoprotein A OS=Mus musculus GN=Snrpa PE=2 SV=3	1.00	0.02	0.03	1.00
Q62261	Spectrin beta chain, brain 1 OS=Mus musculus GN=Sptbn1 PE=1 SV=2	1.00	0.05	0.00	0.00
Q62318	Transcription intermediary factor 1-beta OS=Mus musculus GN=Trim28 PE=1 SV=3	1.00	0.03	1.00	1.00
Q62376	U1 small nuclear ribonucleoprotein 70 kDa OS=Mus musculus GN=Snrnp70 PE=1 SV=2	1.00	0.08	1.00	1.00
Q62446	Peptidyl-prolyl cis-trans isomerase FKBP3 OS=Mus musculus GN=Fkbp3 PE=1 SV=2	1.00	0.02	1.00	1.00
Q62465	Synaptic vesicle membrane protein VAT-1 homolog OS=Mus musculus GN=Vat1 PE=1 SV=3	1.00	0.02	0.00	0.00
Q63836	Selenium-binding protein 2 OS=Mus musculus GN=Selenbp2 PE=1 SV=2	1.00	0.02	0.02	0.02
Q63918	Serum deprivation-response protein OS=Mus musculus GN=Sdpr PE=1 SV=3	1.00	1.00	1.00	0.03
Q64012	RNA-binding protein Raly OS=Mus musculus GN=Raly PE=1 SV=3	0.04	1.00	0.07	1.00
Q640L3	Cell cycle progression protein 1 OS=Mus musculus GN=Ccpg1 PE=1 SV=2	1.00	0.04	1.00	1.00
Q64285	Bile salt-activated lipase OS=Mus musculus GN=Cel PE=1 SV=1	1.00	0.00	0.02	0.00
Q64314	Hematopoietic progenitor cell antigen CD34 OS=Mus musculus GN=Cd34 PE=1 SV=1	1.00	0.07	1.00	1.00
Q64433	10 kDa heat shock protein, mitochondrial OS=Mus musculus GN=Hspe1 PE=1 SV=2	0.02	0.00	0.00	0.00
Q64522	Histone H2A type 2-B OS=Mus musculus GN=Hist2h2ab PE=1 SV=3	0.00	0.00	1.00	0.00
Q64727	Vinculin OS=Mus musculus GN=Vcl PE=1 SV=4	1.00	0.02	0.00	0.00
Q68FD5	Clathrin heavy chain 1 OS=Mus musculus GN=Cltc PE=1 SV=3	1.00	1.00	1.00	0.00
Q6A0C2	MKIAA0136 protein (Fragment) OS=Mus musculus GN=Morc3 PE=2 SV=1	1.00	0.05	1.00	1.00
Q6B966	NACHT, LRR and PYD domains-containing protein 14 OS=Mus musculus GN=Nlrp14 PE=2 SV=2	1.00	1.00	1.00	0.03
Q6IE03	Sentrin/SUMO-specific protease 15 OS=Mus musculus GN=Gm9839 PE=2 SV=1	0.07	0.08	1.00	0.07
Q6IFZ6	Keratin, type II cytoskeletal 1b OS=Mus musculus GN=Krt77 PE=1 SV=1	0.08	1.00	1.00	0.00
Q6IRU2	Tropomyosin alpha-4 chain OS=Mus musculus GN=Tpm4 PE=2 SV=3	1.00	0.00	0.00	0.00
Q6P069	Sorcin OS=Mus musculus GN=Sri PE=1 SV=1	1.00	1.00	0.04	1.00
Q6P5G0	Mitogen-activated protein kinase 4 OS=Mus musculus GN=Mapk4 PE=2 SV=1	1.00	1.00	1.00	0.08
Q6P9N8	Trafficking protein, kinesin binding 2 OS=Mus musculus GN=Trak2 PE=2 SV=1	1.00	0.03	1.00	0.05
Q6PDG5	SWI/SNF complex subunit SMARCC2 OS=Mus musculus GN=Smarcc2 PE=1 SV=2	1.00	0.08	1.00	1.00
Q6PDM2	Serine/arginine-rich splicing factor 1 OS=Mus musculus GN=Srsf1 PE=1 SV=3	0.00	0.00	0.02	1.00
Q6PDN3	Myosin light chain kinase, smooth muscle OS=Mus musculus GN=Mylk PE=1 SV=3	1.00	1.00	0.08	1.00
Q6PGA0	REST corepressor 3 OS=Mus musculus GN=Rcor3 PE=2 SV=2	1.00	1.00	0.08	1.00
Q6PHZ5	Putative RNA-binding protein 15B OS=Mus musculus GN=Rbm15b PE=1 SV=2	1.00	1.00	0.07	1.00
Q6PJ18	Tpm2 protein OS=Mus musculus GN=Tpm2 PE=2 SV=1	1.00	0.02	1.00	0.02
Q6WKZ8	E3 ubiquitin-protein ligase UBR2 OS=Mus musculus GN=Ubr2 PE=1 SV=2	1.00	0.01	1.00	0.01
Q6WVG3	BTB/POZ domain-containing protein KCTD12 OS=Mus musculus GN=Kctd12 PE=1 SV=1	1.00	0.00	0.02	0.02
Q6ZPY7	Lysine-specific demethylase 3B OS=Mus musculus GN=Kdm3b PE=1 SV=2	0.04	0.05	1.00	1.00
Q6ZWN5	40S ribosomal protein S9 OS=Mus musculus GN=Rps9 PE=2 SV=3	1.00	0.02	0.02	0.03
Q6ZWU9	40S ribosomal protein S27 OS=Mus musculus GN=Rps27 PE=1 SV=3	1.00	1.00	0.02	1.00
Q6ZWV3	60S ribosomal protein L10 OS=Mus musculus GN=Rpl10 PE=2 SV=3	1.00	1.00	1.00	0.00
Q6ZWV7	60S ribosomal protein L35 OS=Mus musculus GN=Rpl35 PE=2 SV=1	0.02	0.02	0.02	0.02
Q6ZWX6	Eukaryotic translation initiation factor 2 subunit 1 OS=Mus musculus GN=Eif2s1 PE=1 SV=3	1.00	1.00	0.02	1.00
Q78PY7	Staphylococcal nuclease domain-containing protein 1 OS=Mus musculus GN=Snd1 PE=1 SV=1	1.00	0.00	0.00	1.00
Q792Y9	MCG140783 OS=Mus musculus GN=Gm5771 PE=2 SV=1	1.00	0.02	0.02	1.00
Q792Z0	Trypsinogen 11 OS=Mus musculus GN=Prss3 PE=2 SV=1	1.00	0.00	0.00	1.00
Q792Z1	MCG140784 OS=Mus musculus GN=Try10 PE=2 SV=1	1.00	0.00	0.02	0.02
Q7TMK9	Heterogeneous nuclear ribonucleoprotein Q OS=Mus musculus GN=Syncrip PE=1 SV=2	1.00	0.00	0.02	0.00
Q7TNC4	Putative RNA-binding protein Luc7-like 2 OS=Mus musculus GN=Luc7l2 PE=1 SV=1	1.00	0.02	1.00	1.00
Q7TPR4	Alpha-actinin-1 OS=Mus musculus GN=Actn1 PE=1 SV=1	0.03	1.00	1.00	0.00
Q7TPZ8	Carboxypeptidase A1 OS=Mus musculus GN=Cpa1 PE=2 SV=1	0.02	0.00	0.00	0.00
Q80TN7	Neuron navigator 3 OS=Mus musculus GN=Nav3 PE=1 SV=2	0.04	1.00	1.00	1.00
Q80VM4	Zinc finger protein 579 OS=Mus musculus GN=Znf579 PE=1 SV=1	1.00	1.00	1.00	0.08
Q80WJ7	Protein LYRIC OS=Mus musculus GN=Mtdh PE=1 SV=1	1.00	1.00	0.05	1.00
Q80WT0	Junctophilin-4 OS=Mus musculus GN=Jph4 PE=2 SV=1	1.00	0.07	0.07	1.00
Q80X90	Filamin-B OS=Mus musculus GN=Flnb PE=1 SV=3	1.00	1.00	0.07	0.00

Q80XD0	BC051142 protein OS=Mus musculus GN=BC051142 PE=2 SV=1	1.00	0.01	1.00	1.00
Q80XU3	Nuclear ubiquitous casein and cyclin-dependent kinases substrate OS=Mus musculus GN=Nucks1 PE=1 SV=1	1.00	0.02	1.00	1.00
Q8BFR5	Elongation factor Tu, mitochondrial OS=Mus musculus GN=Tufm PE=1 SV=1	1.00	1.00	0.03	1.00
Q8BFS6	Calcineurin-like phosphoesterase domain-containing protein 1 OS=Mus musculus GN=Cpped1 PE=2 SV=1	1.00	1.00	0.05	1.00
Q8BG05	Heterogeneous nuclear ribonucleoprotein A3 OS=Mus musculus GN=Hnrnpa3 PE=1 SV=1	0.00	0.00	0.00	0.00
Q8BGD9	Eukaryotic translation initiation factor 4B OS=Mus musculus GN=Eif4b PE=1 SV=1	0.04	1.00	1.00	1.00
Q8BGS0	Protein MAK16 homolog OS=Mus musculus GN=Mak16 PE=2 SV=1	1.00	1.00	1.00	0.07
Q8BH64	EH domain-containing protein 2 OS=Mus musculus GN=Ehd2 PE=1 SV=1	1.00	1.00	1.00	0.00
Q8BH95	Enoyl-CoA hydratase, mitochondrial OS=Mus musculus GN=Echs1 PE=1 SV=1	1.00	1.00	0.02	1.00
Q8BH97	Reticulocalbin-3 OS=Mus musculus GN=Rcn3 PE=2 SV=1	1.00	0.00	0.02	0.00
Q8BHN3	Neutral alpha-glucosidase AB OS=Mus musculus GN=Ganab PE=1 SV=1	1.00	0.04	1.00	1.00
Q8BJU0	Small glutamine-rich tetratricopeptide repeat-containing protein alpha OS=Mus musculus GN=Sgta PE=1 SV=2	1.00	0.07	1.00	1.00
Q8BK67	Protein RCC2 OS=Mus musculus GN=Rcc2 PE=2 SV=1	1.00	0.06	1.00	1.00
Q8BL97	Serine/arginine-rich splicing factor 7 OS=Mus musculus GN=Srsf7 PE=1 SV=1	1.00	0.00	0.03	1.00
Q8BMK4	Cytoskeleton-associated protein 4 OS=Mus musculus GN=Ckap4 PE=2 SV=2	0.00	0.00	0.00	0.00
Q8BMS1	Trifunctional enzyme subunit alpha, mitochondrial OS=Mus musculus GN=Hadha PE=1 SV=1	1.00	1.00	0.00	0.00
Q8BP67	60S ribosomal protein L24 OS=Mus musculus GN=Rpl24 PE=2 SV=2	1.00	1.00	0.07	0.02
Q8BP92	Reticulocalbin-2 OS=Mus musculus GN=Rcn2 PE=2 SV=1	1.00	1.00	1.00	0.02
Q8BSY0	Aspartyl/asparaginyl beta-hydroxylase OS=Mus musculus GN=Asph PE=2 SV=1	1.00	1.00	1.00	0.02
Q8BTF7	DNA ligase 4 OS=Mus musculus GN=Lig4 PE=2 SV=1	1.00	1.00	0.07	1.00
Q8BTM8	Filamin-A OS=Mus musculus GN=Flna PE=1 SV=5	1.00	0.00	0.00	0.00
Q8BUE4	Apoptosis-inducing factor 2 OS=Mus musculus GN=Aifm2 PE=2 SV=1	1.00	1.00	0.05	0.02
Q8BWT1	3-ketoacyl-CoA thiolase, mitochondrial OS=Mus musculus GN=Acaa2 PE=1 SV=3	1.00	1.00	1.00	0.00
Q8BX57	PX domain-containing protein kinase-like protein OS=Mus musculus GN=Pxk PE=1 SV=2	1.00	0.08	1.00	1.00
Q8C0M9	L-asparaginase OS=Mus musculus GN=Asrgl1 PE=1 SV=1	1.00	1.00	1.00	0.04
Q8C138	Uncharacterized protein C6orf105 homolog OS=Mus musculus PE=2 SV=2	1.00	1.00	1.00	0.03
Q8C1B7	Septin-11 OS=Mus musculus GN=Sept11 PE=1 SV=4	1.00	0.00	0.02	0.02
Q8C635	Glycerol kinase-like 1 OS=Mus musculus GN=Gykl1 PE=2 SV=1	1.00	1.00	0.07	1.00
Q8CCT4	Transcription elongation factor A protein-like 5 OS=Mus musculus GN=Tceal5 PE=1 SV=1	0.02	1.00	1.00	1.00
Q8CDN6	Thioredoxin-like protein 1 OS=Mus musculus GN=Txnl1 PE=1 SV=3	1.00	0.02	0.00	1.00
Q8CE23	Orexigenic neuropeptide QRFP OS=Mus musculus GN=Qrfp PE=2 SV=1	1.00	0.08	1.00	1.00
Q8CGP2	Histone H2B type 1-P OS=Mus musculus GN=Hist1h2bp PE=1 SV=3	0.00	0.00	0.00	0.00
Q8CIE6	Coatomer subunit alpha OS=Mus musculus GN=Copa PE=1 SV=2	1.00	1.00	1.00	0.02
Q8K0E8	Fibrinogen beta chain OS=Mus musculus GN=Fgb PE=2 SV=1	1.00	1.00	1.00	0.02
Q8K0Z5	Tropomyosin 3, gamma OS=Mus musculus GN=Tpm3 PE=2 SV=1	1.00	0.03	0.05	0.08
Q8K2B3	Succinate dehydrogenase [ubiquinone] flavoprotein subunit, mitochondrial OS=Mus musculus GN=Sdha PE=1 S	0.03	0.02	0.02	0.03
Q8K3P1	P2X purinoceptor OS=Mus musculus GN=P2rx2 PE=2 SV=2	0.05	0.08	1.00	1.00
Q8K4L4	Protein POF1B OS=Mus musculus GN=Pof1b PE=1 SV=3	1.00	1.00	0.02	1.00
Q8K4Z0	Leucine-rich repeat LGI family member 2 OS=Mus musculus GN=Lgi2 PE=2 SV=1	1.00	1.00	1.00	0.01
Q8QZT1	Acetyl-CoA acetyltransferase, mitochondrial OS=Mus musculus GN=Acat1 PE=1 SV=1	1.00	0.00	1.00	0.00
Q8R050	Eukaryotic peptide chain release factor GTP-binding subunit ERF3A OS=Mus musculus GN=Gspt1 PE=1 SV=1	1.00	0.07	1.00	1.00
Q8R081	Heterogeneous nuclear ribonucleoprotein L OS=Mus musculus GN=Hnrnpl PE=1 SV=2	1.00	0.00	1.00	1.00
Q8R1M2	Histone H2A.J OS=Mus musculus GN=H2afj PE=2 SV=1	0.02	0.02	1.00	0.02
Q8R207	Small subunit of serine palmitoyltransferase A OS=Mus musculus GN=Ssspta PE=2 SV=2	1.00	0.02	0.02	0.02
Q8R370	Usher syndrome type-1C protein-binding protein 1 OS=Mus musculus GN=Ushbp1 PE=1 SV=2	1.00	1.00	0.03	1.00
Q8R5C5	Beta-centractin OS=Mus musculus GN=Actr1b PE=1 SV=1	1.00	0.02	0.03	1.00
Q8VCQ6	Phosphatidylcholine:ceramide cholinephosphotransferase 1 OS=Mus musculus GN=Sgms1 PE=1 SV=2	0.02	1.00	1.00	1.00
Q8VCQ8	Caldesmon 1 OS=Mus musculus GN=Cald1 PE=2 SV=1	1.00	0.00	0.02	0.00
Q8VDD5	Myosin-9 OS=Mus musculus GN=Myh9 PE=1 SV=4	1.00	1.00	0.00	0.00
Q8VDN2	Sodium/potassium-transporting ATPase subunit alpha-1 OS=Mus musculus GN=Atp1a1 PE=1 SV=1	1.00	1.00	1.00	0.02
Q8VDP4	Protein KIAA1967 homolog OS=Mus musculus PE=1 SV=2	1.00	1.00	1.00	0.02
Q8VE97	Serine/arginine-rich splicing factor 4 OS=Mus musculus GN=Srsf4 PE=2 SV=1	0.07	0.03	0.02	0.02
Q8VEK3	Heterogeneous nuclear ribonucleoprotein U OS=Mus musculus GN=Hnrnpu PE=1 SV=1	0.03	0.00	0.00	0.00
Q8VF84	MCG60141 OS=Mus musculus GN=Olfr1317 PE=3 SV=1	1.00	1.00	0.08	1.00
Q8VFZ5	MCG141584 OS=Mus musculus GN=Olfr516 PE=3 SV=1	1.00	0.04	1.00	1.00
Q8VHM5	Heterogeneous nuclear ribonucleoprotein R OS=Mus musculus GN=Hnrnpr PE=2 SV=1	1.00	0.00	0.05	0.00
Q8VIJ6	Splicing factor, proline- and glutamine-rich OS=Mus musculus GN=Sfpq PE=1 SV=1	0.02	0.00	0.00	0.02
Q91V14	Solute carrier family 12 member 5 OS=Mus musculus GN=Slc12a5 PE=1 SV=2	1.00	0.01	1.00	1.00
Q91V41	Ras-related protein Rab-14 OS=Mus musculus GN=Rab14 PE=1 SV=3	1.00	0.02	1.00	1.00
Q91V55	Ribosomal protein S5 OS=Mus musculus GN=Rps5 PE=2 SV=1	1.00	1.00	1.00	0.02
Q91V64	Isochorismatase domain-containing protein 1 OS=Mus musculus GN=Isoc1 PE=2 SV=1	1.00	1.00	1.00	0.02
Q91VB8	Alpha globin 1 OS=Mus musculus GN=Hba-a1 PE=2 SV=1	0.02	0.02	0.00	0.00
Q91VI7	Ribonuclease inhibitor OS=Mus musculus GN=Rnh1 PE=1 SV=1	1.00	1.00	0.00	0.02
Q91VM5	Heterogeneous nuclear ribonucleoprotein G-like 1 OS=Mus musculus GN=Rbmxl1 PE=2 SV=1	1.00	0.08	0.02	0.00
Q91VM9	Inorganic pyrophosphatase 2, mitochondrial OS=Mus musculus GN=Ppa2 PE=2 SV=1	1.00	1.00	0.08	1.00
Q91W90	Thioredoxin domain-containing protein 5 OS=Mus musculus GN=Txndc5 PE=1 SV=2	1.00	0.00	0.02	0.00
Q91WJ8	Far upstream element-binding protein 1 OS=Mus musculus GN=Fubp1 PE=1 SV=1	1.00	0.03	1.00	0.02
Q91WQ3	Tyrosyl-tRNA synthetase, cytoplasmic OS=Mus musculus GN=Yars PE=2 SV=3	1.00	1.00	0.05	1.00
Q91X79	Elastase 1, pancreatic OS=Mus musculus GN=Cela1 PE=2 SV=1	1.00	0.00	0.02	0.00
Q91XV3	Brain acid soluble protein 1 OS=Mus musculus GN=Basp1 PE=1 SV=3	0.00	0.00	0.00	0.00
Q91Y47	Coagulation factor XI OS=Mus musculus GN=F11 PE=2 SV=2	1.00	0.03	1.00	1.00

Q91YD9	Neural Wiskott-Aldrich syndrome protein OS=Mus musculus GN=Wasl PE=1 SV=1	1.00	1.00	0.05	1.00
Q91YQ5	Dolichyl-diphosphooligosaccharide--protein glycosyltransferase subunit 1 OS=Mus musculus GN=Rpn1 PE=2 SV	1.00	0.04	0.03	0.00
Q921B4	Neurotrophin receptor-interacting factor 2 OS=Mus musculus GN=Nrif2 PE=1 SV=1	0.03	0.04	0.05	0.03
Q921F2	TAR DNA-binding protein 43 OS=Mus musculus GN=Tardbp PE=1 SV=1	1.00	0.02	1.00	0.02
Q921I1	Serotransferrin OS=Mus musculus GN=Tf PE=1 SV=1	1.00	0.07	1.00	1.00
Q922F4	Tubulin beta-6 chain OS=Mus musculus GN=Tubb6 PE=1 SV=1	1.00	1.00	1.00	0.04
Q922I7	MCG13402, isoform CRA_c OS=Mus musculus GN=Ptbp1 PE=2 SV=1	0.02	0.00	0.00	0.02
Q922U2	Keratin, type II cytoskeletal 5 OS=Mus musculus GN=Krt5 PE=1 SV=1	0.02	0.00	0.00	0.00
Q923D4	Splicing factor 3B subunit 5 OS=Mus musculus GN=Sf3b5 PE=2 SV=1	1.00	0.02	1.00	1.00
Q93092	Transaldolase OS=Mus musculus GN=Taldo1 PE=1 SV=2	1.00	0.04	1.00	0.00
Q99020	Heterogeneous nuclear ribonucleoprotein A/B OS=Mus musculus GN=Hnrnpab PE=1 SV=1	1.00	0.00	1.00	0.00
Q99J77	N-acetylneuraminic acid synthase (Sialic acid synthase) OS=Mus musculus GN=Nans PE=2 SV=1	1.00	1.00	1.00	0.02
Q99JF8	PC4 and SFRS1-interacting protein OS=Mus musculus GN=Psip1 PE=1 SV=1	1.00	1.00	0.04	1.00
Q99JI6	Ras-related protein Rap-1b OS=Mus musculus GN=Rap1b PE=2 SV=2	1.00	0.02	0.02	0.02
Q99JP7	Gamma-glutamyltransferase 7 OS=Mus musculus GN=Ggt7 PE=1 SV=2	1.00	0.07	1.00	1.00
Q99JY9	Actin-related protein 3 OS=Mus musculus GN=Actr3 PE=1 SV=3	1.00	1.00	1.00	0.00
Q99K48	Non-POU domain-containing octamer-binding protein OS=Mus musculus GN=Nono PE=1 SV=3	1.00	0.00	0.03	1.00
Q99KC8	von Willebrand factor A domain-containing protein 5A OS=Mus musculus GN=Vwa5a PE=1 SV=2	1.00	1.00	1.00	0.02
Q99KF0	Caspase recruitment domain-containing protein 14 OS=Mus musculus GN=Card14 PE=2 SV=2	1.00	1.00	0.03	1.00
Q99KF1	Transmembrane emp24 domain-containing protein 9 OS=Mus musculus GN=Tmed9 PE=2 SV=2	1.00	0.00	0.02	0.00
Q99KI0	Aconitate hydratase, mitochondrial OS=Mus musculus GN=Aco2 PE=1 SV=1	1.00	1.00	0.02	0.04
Q99KJ8	Dynactin subunit 2 OS=Mus musculus GN=Dctn2 PE=1 SV=3	1.00	1.00	1.00	0.02
Q99KV1	DnaJ homolog subfamily B member 11 OS=Mus musculus GN=Dnajb11 PE=1 SV=1	1.00	1.00	1.00	0.04
Q99L47	Hsc70-interacting protein OS=Mus musculus GN=St13 PE=2 SV=1	1.00	1.00	1.00	0.02
Q99LC5	Electron transfer flavoprotein subunit alpha, mitochondrial OS=Mus musculus GN=Etfa PE=1 SV=2	1.00	0.02	1.00	0.02
Q99LD8	N(G),N(G)-dimethylarginine dimethylaminohydrolase 2 OS=Mus musculus GN=Ddah2 PE=1 SV=1	1.00	1.00	0.02	1.00
Q99LJ6	Glutathione peroxidase 7 OS=Mus musculus GN=Gpx7 PE=2 SV=1	1.00	0.08	1.00	1.00
Q99LX0	Protein DJ-1 OS=Mus musculus GN=Park7 PE=1 SV=1	1.00	1.00	0.04	0.00
Q99M74	Keratin, type II cuticular Hb2 OS=Mus musculus GN=Krt82 PE=2 SV=2	1.00	0.03	0.06	1.00
Q99MD9	Nuclear autoantigenic sperm protein OS=Mus musculus GN=Nasp PE=1 SV=2	1.00	0.00	0.03	1.00
Q99MR6	Serrate RNA effector molecule homolog OS=Mus musculus GN=Srrt PE=1 SV=1	0.06	1.00	1.00	1.00
Q99P72	Reticulon-4 OS=Mus musculus GN=Rtn4 PE=1 SV=2	1.00	0.02	1.00	0.00
Q99PL5	Ribosome-binding protein 1 OS=Mus musculus GN=Rrbp1 PE=2 SV=2	1.00	0.00	0.00	0.00
Q99PS0	Keratin, type I cytoskeletal 23 OS=Mus musculus GN=Krt23 PE=2 SV=1	1.00	1.00	1.00	0.02
Q99PT1	Rho GDP-dissociation inhibitor 1 OS=Mus musculus GN=Arhgdia PE=1 SV=3	0.03	0.00	1.00	0.00
Q9CPN9	RIKEN cDNA 2210010C04, isoform CRA_b OS=Mus musculus GN=2210010C04Rik PE=2 SV=1	1.00	0.06	0.03	0.00
Q9CPQ1	Cytochrome c oxidase subunit 6C OS=Mus musculus GN=Cox6c PE=1 SV=3	0.04	0.02	1.00	1.00
Q9CPQ5	Centromere protein Q OS=Mus musculus GN=Cenpq PE=2 SV=1	1.00	1.00	1.00	0.04
Q9CPR4	60S ribosomal protein L17 OS=Mus musculus GN=Rpl17 PE=2 SV=3	1.00	0.02	0.00	0.00
Q9CPV4	Glyoxalase domain-containing protein 4 OS=Mus musculus GN=Glod4 PE=2 SV=1	1.00	1.00	0.08	1.00
Q9CPW4	Actin-related protein 2/3 complex subunit 5 OS=Mus musculus GN=Arpc5 PE=2 SV=3	1.00	1.00	0.02	1.00
Q9CQ52	Elastase 3, pancreatic OS=Mus musculus GN=Cela3b PE=2 SV=1	1.00	0.00	0.00	0.00
Q9CQ65	S-methyl-5'-thioadenosine phosphorylase OS=Mus musculus GN=Mtap PE=2 SV=1	1.00	1.00	1.00	0.02
Q9CQC2	Colipase OS=Mus musculus GN=Clps PE=2 SV=1	1.00	0.00	1.00	1.00
Q9CQF9	Prenylcysteine oxidase OS=Mus musculus GN=Pcyox1 PE=1 SV=1	1.00	1.00	1.00	0.02
Q9CQI6	Coactosin-like protein OS=Mus musculus GN=Cotl1 PE=1 SV=3	1.00	0.07	1.00	1.00
Q9CQQ7	ATP synthase subunit b, mitochondrial OS=Mus musculus GN=Atp5f1 PE=1 SV=1	1.00	1.00	0.05	1.00
Q9CQR2	40S ribosomal protein S21 OS=Mus musculus GN=Rps21 PE=2 SV=1	1.00	0.00	0.00	0.00
Q9CQV8	14-3-3 protein beta/alpha OS=Mus musculus GN=Ywhab PE=1 SV=3	0.02	0.00	0.00	0.00
Q9CR35	Chymotrypsinogen B OS=Mus musculus GN=Ctrb1 PE=2 SV=1	0.00	0.00	0.00	0.00
Q9CR57	60S ribosomal protein L14 OS=Mus musculus GN=Rpl14 PE=2 SV=3	1.00	0.00	0.00	0.00
Q9CRB6	Tubulin polymerization-promoting protein family member 3 OS=Mus musculus GN=Tppp3 PE=1 SV=1	1.00	0.02	0.00	0.00
Q9CS72	Filamin-A-interacting protein 1 OS=Mus musculus GN=Filip1 PE=1 SV=2	0.07	0.08	1.00	1.00
Q9CWF2	Tubulin beta-2B chain OS=Mus musculus GN=Tubb2b PE=1 SV=1	1.00	0.00	0.02	0.00
Q9CWZ3	RNA-binding protein 8A OS=Mus musculus GN=Rbm8a PE=1 SV=3	1.00	0.02	1.00	1.00
Q9CX80	Cytoglobin OS=Mus musculus GN=Cygb PE=2 SV=1	1.00	1.00	1.00	0.02
Q9CX86	Uncharacterized protein OS=Mus musculus GN=Hnrnpa0 PE=2 SV=1	1.00	1.00	1.00	0.02
Q9CXW4	60S ribosomal protein L11 OS=Mus musculus GN=Rpl11 PE=1 SV=4	1.00	0.04	1.00	0.02
Q9CY50	Translocon-associated protein subunit alpha OS=Mus musculus GN=Ssr1 PE=1 SV=1	0.06	0.00	0.02	0.00
Q9CY58	Plasminogen activator inhibitor 1 RNA-binding protein OS=Mus musculus GN=Serbp1 PE=1 SV=2	0.07	0.00	1.00	0.00
Q9CYZ2	Tumor protein D54 OS=Mus musculus GN=Tpd52l2 PE=1 SV=1	1.00	1.00	0.02	0.04
Q9CZ13	Cytochrome b-c1 complex subunit 1, mitochondrial OS=Mus musculus GN=Uqcrc1 PE=1 SV=2	1.00	1.00	0.02	1.00
Q9CZ69	CKLF-like MARVEL transmembrane domain-containing protein 6 OS=Mus musculus GN=Cmtm6 PE=2 SV=1	1.00	1.00	1.00	0.04
Q9CZM2	60S ribosomal protein L15 OS=Mus musculus GN=Rpl15 PE=2 SV=4	1.00	1.00	0.07	1.00
Q9CZN7	Serine hydroxymethyltransferase OS=Mus musculus GN=Shmt2 PE=2 SV=1	1.00	0.06	1.00	1.00
Q9CZX8	40S ribosomal protein S19 OS=Mus musculus GN=Rps19 PE=1 SV=3	0.00	0.00	0.02	0.00
Q9D051	Pyruvate dehydrogenase E1 component subunit beta, mitochondrial OS=Mus musculus GN=Pdhb PE=1 SV=1	1.00	1.00	0.06	1.00
Q9D0E1	Heterogeneous nuclear ribonucleoprotein M OS=Mus musculus GN=Hnrnpm PE=1 SV=3	1.00	0.08	0.02	0.07
Q9D0F9	Phosphoglucomutase-1 OS=Mus musculus GN=Pgm1 PE=1 SV=4	1.00	0.08	0.02	1.00
Q9D0J8	Parathymosin OS=Mus musculus GN=Ptms PE=2 SV=3	0.02	0.02	0.02	0.02
Q9D0K2	Succinyl-CoA:3-ketoacid-coenzyme A transferase 1, mitochondrial OS=Mus musculus GN=Oxct1 PE=1 SV=1	1.00	0.02	1.00	1.00

Q9D0T1	NHP2-like protein 1 OS=Mus musculus GN=Nhp2l1 PE=2 SV=4	1.00	1.00	0.04	1.00
Q9D1D4	Transmembrane emp24 domain-containing protein 10 OS=Mus musculus GN=Tmed10 PE=2 SV=1	1.00	0.00	1.00	0.00
Q9D1D6	Collagen triple helix repeat-containing protein 1 OS=Mus musculus GN=Cthrc1 PE=2 SV=1	1.00	0.05	1.00	1.00
Q9D1J3	SAP domain-containing ribonucleoprotein OS=Mus musculus GN=Sarnp PE=1 SV=3	1.00	0.03	0.02	1.00
Q9D1M0	Protein SEC13 homolog OS=Mus musculus GN=Sec13 PE=2 SV=3	1.00	1.00	0.02	1.00
Q9D1R9	60S ribosomal protein L34 OS=Mus musculus GN=Rpl34 PE=3 SV=2	1.00	0.02	0.02	0.06
Q9D281	Protein Noxp20 OS=Mus musculus GN=Fam114a1 PE=2 SV=1	1.00	1.00	1.00	0.02
Q9D2M8	Ubiquitin-conjugating enzyme E2 variant 2 OS=Mus musculus GN=Ube2v2 PE=2 SV=4	1.00	0.02	0.02	0.04
Q9D358	Low molecular weight phosphotyrosine protein phosphatase OS=Mus musculus GN=Acp1 PE=2 SV=3	1.00	0.04	1.00	1.00
Q9D3D9	ATP synthase subunit delta, mitochondrial OS=Mus musculus GN=Atp5d PE=1 SV=1	0.02	0.00	0.03	1.00
Q9D662	Protein transport protein Sec23B OS=Mus musculus GN=Sec23b PE=2 SV=1	1.00	1.00	1.00	0.02
Q9D6N5	Dr1-associated corepressor OS=Mus musculus GN=Drap1 PE=2 SV=3	1.00	0.05	1.00	1.00
Q9D773	39S ribosomal protein L2, mitochondrial OS=Mus musculus GN=Mrpl2 PE=2 SV=1	1.00	1.00	0.03	0.08
Q9D8B3	Charged multivesicular body protein 4b OS=Mus musculus GN=Chmp4b PE=2 SV=2	1.00	0.02	0.02	0.02
Q9D8E6	60S ribosomal protein L4 OS=Mus musculus GN=Rpl4 PE=1 SV=3	1.00	1.00	0.02	0.02
Q9D8N0	Elongation factor 1-gamma OS=Mus musculus GN=Eef1g PE=1 SV=3	1.00	0.02	0.00	0.04
Q9D8U4	Complement C1q tumor necrosis factor-related protein 2 OS=Mus musculus GN=C1qtnf2 PE=2 SV=1	1.00	0.02	0.03	1.00
Q9DAK9	14 kDa phosphohistidine phosphatase OS=Mus musculus GN=Phpt1 PE=1 SV=1	1.00	0.04	1.00	1.00
Q9DAW9	Calponin-3 OS=Mus musculus GN=Cnn3 PE=2 SV=1	1.00	0.00	1.00	1.00
Q9DB77	Cytochrome b-c1 complex subunit 2, mitochondrial OS=Mus musculus GN=Uqcrc2 PE=1 SV=1	0.04	0.02	0.02	1.00
Q9DBG5	Perilipin-3 OS=Mus musculus GN=Plin3 PE=1 SV=1	1.00	1.00	0.03	1.00
Q9DBG6	Dolichyl-diphosphooligosaccharide--protein glycosyltransferase subunit 2 OS=Mus musculus GN=Rpn2 PE=2 SV	0.03	1.00	1.00	0.02
Q9DBJ1	Phosphoglycerate mutase 1 OS=Mus musculus GN=Pgam1 PE=1 SV=3	0.00	0.00	0.00	0.00
Q9DBP5	UMP-CMP kinase OS=Mus musculus GN=Cmpk1 PE=1 SV=1	1.00	1.00	0.05	1.00
Q9DBZ1	Inhibitor of nuclear factor kappa-B kinase-interacting protein OS=Mus musculus GN=Ikbip PE=1 SV=2	1.00	1.00	1.00	0.07
Q9DC29	ATP-binding cassette sub-family B member 6, mitochondrial OS=Mus musculus GN=Abcb6 PE=1 SV=1	1.00	0.03	1.00	1.00
Q9DCD0	6-phosphogluconate dehydrogenase, decarboxylating OS=Mus musculus GN=Pgd PE=2 SV=3	1.00	1.00	0.02	0.02
Q9DCF9	Translocon-associated protein subunit gamma OS=Mus musculus GN=Ssr3 PE=1 SV=1	1.00	1.00	1.00	0.02
Q9DCL9	Multifunctional protein ADE2 OS=Mus musculus GN=Paics PE=1 SV=4	1.00	1.00	1.00	0.02
Q9DCN2	NADH-cytochrome b5 reductase 3 OS=Mus musculus GN=Cyb5r3 PE=1 SV=3	1.00	0.04	0.00	0.00
Q9DCQ2	Putative L-aspartate dehydrogenase OS=Mus musculus GN=Aspdh PE=1 SV=1	1.00	0.04	0.08	1.00
Q9DCV7	Keratin, type II cytoskeletal 7 OS=Mus musculus GN=Krt7 PE=1 SV=1	1.00	1.00	1.00	0.00
Q9DCW4	Electron transfer flavoprotein subunit beta OS=Mus musculus GN=Etfb PE=1 SV=3	1.00	0.00	0.00	0.00
Q9DCX2	ATP synthase subunit d, mitochondrial OS=Mus musculus GN=Atp5h PE=1 SV=3	1.00	1.00	0.02	1.00
Q9EPC2	Fibroblast growth factor 23 OS=Mus musculus GN=Fgf23 PE=1 SV=1	1.00	0.03	1.00	1.00
Q9EQU5	Protein SET OS=Mus musculus GN=Set PE=1 SV=1	0.02	0.00	0.02	0.00
Q9ER05	Chymopasin OS=Mus musculus GN=Ctrl PE=2 SV=1	1.00	0.02	0.02	0.00
Q9EST1	Gasdermin-A OS=Mus musculus GN=Gsdma PE=2 SV=1	1.00	1.00	0.06	0.00
Q9EST5	Acidic leucine-rich nuclear phosphoprotein 32 family member B OS=Mus musculus GN=Anp32b PE=1 SV=1	1.00	0.02	0.03	1.00
Q9JHJ0	Tropomodulin-3 OS=Mus musculus GN=Tmod3 PE=1 SV=1	1.00	1.00	1.00	0.05
Q9JHU4	Cytoplasmic dynein 1 heavy chain 1 OS=Mus musculus GN=Dync1h1 PE=1 SV=2	1.00	1.00	1.00	0.02
Q9JHU9	Inositol-3-phosphate synthase 1 OS=Mus musculus GN=Isyna1 PE=2 SV=1	0.07	1.00	1.00	1.00
Q9JII6	Alcohol dehydrogenase [NADP+] OS=Mus musculus GN=Akr1a1 PE=1 SV=3	1.00	0.00	0.04	0.07
Q9JIW9	Ras-related protein Ral-B OS=Mus musculus GN=Ralb PE=2 SV=1	1.00	0.05	1.00	1.00
Q9JJG7	Calcium-binding protein 8 OS=Mus musculus GN=Caln1 PE=2 SV=1	1.00	0.02	1.00	1.00
Q9JJU8	SH3 domain-binding glutamic acid-rich-like protein OS=Mus musculus GN=Sh3bgrl PE=2 SV=1	1.00	0.02	0.02	0.02
Q9JJW6	RNA and export factor-binding protein 2 OS=Mus musculus GN=Refbp2 PE=1 SV=1	1.00	0.02	0.02	0.06
Q9JK81	UPF0160 protein MYG1, mitochondrial OS=Mus musculus GN=Myg1 PE=2 SV=1	1.00	0.03	1.00	1.00
Q9JK88	Serpin I2 OS=Mus musculus GN=Serpini2 PE=1 SV=1	1.00	0.03	1.00	1.00
Q9JLJ2	4-trimethylaminobutyraldehyde dehydrogenase OS=Mus musculus GN=Aldh9a1 PE=1 SV=1	1.00	0.03	1.00	1.00
Q9JMA4	Killer cell lectin-like receptor, subfamily A, member 17 OS=Mus musculus GN=Klra17 PE=2 SV=1	1.00	1.00	0.02	1.00
Q9JMG7	Hepatoma-derived growth factor-related protein 3 OS=Mus musculus GN=Hdgfrp3 PE=1 SV=2	1.00	1.00	0.06	1.00
Q9QUI0	Transforming protein RhoA OS=Mus musculus GN=Rhoa PE=1 SV=1	1.00	0.04	0.00	0.02
Q9QUM9	Proteasome subunit alpha type-6 OS=Mus musculus GN=Psma6 PE=1 SV=1	1.00	1.00	0.02	0.02
Q9QXL1	Kinesin-like protein KIF21B OS=Mus musculus GN=Kif21b PE=1 SV=2	1.00	1.00	0.08	1.00
Q9QXS1	Plectin OS=Mus musculus GN=Plec PE=1 SV=2	1.00	1.00	0.04	1.00
Q9QXT0	Protein canopy homolog 2 OS=Mus musculus GN=Cnpy2 PE=2 SV=1	1.00	0.03	1.00	0.02
Q9QYB1	Chloride intracellular channel protein 4 OS=Mus musculus GN=Clic4 PE=1 SV=3	1.00	1.00	1.00	0.02
Q9QYB5	Gamma-adducin OS=Mus musculus GN=Add3 PE=1 SV=2	1.00	1.00	1.00	0.00
Q9QYC0	Alpha-adducin OS=Mus musculus GN=Add1 PE=1 SV=2	1.00	1.00	0.00	0.00
Q9QZD9	Eukaryotic translation initiation factor 3 subunit I OS=Mus musculus GN=Eif3i PE=1 SV=1	1.00	0.08	1.00	1.00
Q9R0N0	Galactokinase OS=Mus musculus GN=Galk1 PE=2 SV=1	1.00	0.03	1.00	1.00
Q9R0P3	S-formylglutathione hydrolase OS=Mus musculus GN=Esd PE=2 SV=1	1.00	1.00	0.05	1.00
Q9R0P5	Destrin OS=Mus musculus GN=Dstn PE=1 SV=3	1.00	1.00	1.00	0.00
Q9R0P6	Signal peptidase complex catalytic subunit SEC11A OS=Mus musculus GN=Sec11a PE=2 SV=1	1.00	0.03	1.00	1.00
Q9R0Q3	Transmembrane emp24 domain-containing protein 2 OS=Mus musculus GN=Tmed2 PE=1 SV=1	0.02	0.00	0.02	0.03
Q9R0T7	MCG15085 OS=Mus musculus GN=Try4 PE=2 SV=1	1.00	1.00	1.00	0.00
Q9R0T8	Inhibitor of nuclear factor kappa-B kinase subunit epsilon OS=Mus musculus GN=Ikbke PE=2 SV=2	1.00	0.03	0.04	0.05
Q9R0Y5	Adenylate kinase isoenzyme 1 OS=Mus musculus GN=Ak1 PE=1 SV=1	1.00	1.00	1.00	0.03
Q9R1P0	Proteasome subunit alpha type-4 OS=Mus musculus GN=Psma4 PE=1 SV=1	1.00	0.00	1.00	1.00
Q9R1P3	Proteasome subunit beta type-2 OS=Mus musculus GN=Psmb2 PE=1 SV=1	1.00	0.02	0.02	0.02

Q9R1P4	Proteasome subunit alpha type-1 OS=Mus musculus GN=Psma1 PE=1 SV=1	1.00	0.00	0.07	1.00
Q9WTL8	Aryl hydrocarbon receptor nuclear translocator-like protein 1 OS=Mus musculus GN=Arntl PE=1 SV=2	1.00	1.00	1.00	0.07
Q9WTP6	Adenylate kinase 2, mitochondrial OS=Mus musculus GN=Ak2 PE=1 SV=5	1.00	0.00	1.00	1.00
Q9WTQ5	A-kinase anchor protein 12 OS=Mus musculus GN=Akap12 PE=1 SV=1	1.00	0.00	0.00	0.00
Q9WTX5	S-phase kinase-associated protein 1 OS=Mus musculus GN=Skp1 PE=1 SV=3	1.00	0.02	0.02	1.00
Q9WUK2	Eukaryotic translation initiation factor 4H OS=Mus musculus GN=Eif4h PE=1 SV=3	1.00	1.00	1.00	0.02
Q9WUM4	Coronin-1C OS=Mus musculus GN=Coro1c PE=1 SV=2	1.00	1.00	1.00	0.02
Q9WUU7	Cathepsin Z OS=Mus musculus GN=Ctsz PE=2 SV=1	1.00	0.02	1.00	1.00
Q9WV80	Sorting nexin-1 OS=Mus musculus GN=Snx1 PE=1 SV=1	1.00	1.00	0.02	0.02
Q9WVA4	Transgelin-2 OS=Mus musculus GN=Tagln2 PE=1 SV=4	1.00	0.00	0.00	0.00
Q9WVK4	EH domain-containing protein 1 OS=Mus musculus GN=Ehd1 PE=1 SV=1	1.00	1.00	1.00	0.00
Q9Z0E6	Interferon-induced guanylate-binding protein 2 OS=Mus musculus GN=Gbp2 PE=1 SV=1	1.00	1.00	1.00	0.00
Q9Z0X1	Apoptosis-inducing factor 1, mitochondrial OS=Mus musculus GN=Aifm1 PE=1 SV=1	1.00	0.00	1.00	0.06
Q9Z1N5	Spliceosome RNA helicase Ddx39b OS=Mus musculus GN=Ddx39b PE=1 SV=1	0.05	0.03	1.00	1.00
Q9Z1Q5	Chloride intracellular channel protein 1 OS=Mus musculus GN=Clic1 PE=1 SV=3	1.00	0.00	0.02	0.02
Q9Z1Q9	Valyl-tRNA synthetase OS=Mus musculus GN=Vars PE=2 SV=1	1.00	0.02	0.02	1.00
Q9Z1R9	MCG124046 OS=Mus musculus GN=Prss1 PE=2 SV=1	1.00	0.02	1.00	0.02
Q9Z1T2	Thrombospondin-4 OS=Mus musculus GN=Thbs4 PE=2 SV=1	1.00	0.05	1.00	1.00
Q9Z1Z2	Serine-threonine kinase receptor-associated protein OS=Mus musculus GN=Strap PE=1 SV=2	1.00	0.02	1.00	1.00
Q9Z204	Heterogeneous nuclear ribonucleoproteins C1/C2 OS=Mus musculus GN=Hnrnpc PE=1 SV=1	1.00	0.00	1.00	0.00
Q9Z2M7	Phosphomannomutase 2 OS=Mus musculus GN=Pmm2 PE=2 SV=1	1.00	1.00	0.06	1.00
Q9Z2U0	Proteasome subunit alpha type-7 OS=Mus musculus GN=Psma7 PE=1 SV=1	1.00	0.00	0.00	0.00
Q9Z2U1	Proteasome subunit alpha type-5 OS=Mus musculus GN=Psma5 PE=1 SV=1	1.00	1.00	1.00	0.02
Q9Z2W0	Aspartyl aminopeptidase OS=Mus musculus GN=Dnpep PE=2 SV=2	1.00	1.00	0.02	1.00
Q9Z2X1	Heterogeneous nuclear ribonucleoprotein F OS=Mus musculus GN=Hnrnpf PE=1 SV=3	1.00	0.02	1.00	0.02

Unique Peptides				Spectral Counts				Normalized Spectral Counts				
e15.5	e17.5	p2	p14	e15.5	e17.5	p2	p14	e15.5	e17.5	p2	p14	gene
0	0	1	0	0	0	1	0	0.0000	0.0000	0.0003	0.0000	Tbc1d16
0	1	0	0	0	2	0	0	0.0000	0.0003	0.0000	0.0000	Fsip2
1	0	0	0	1	0	0	0	0.0008	0.0000	0.0000	0.0000	Agrn
0	5	3	10	0	23	14	47	0.0000	0.0032	0.0040	0.0132	Hbb-b1
0	11	7	8	0	92	32	18	0.0000	0.0129	0.0091	0.0051	Cpb1
0	0	0	1	0	0	0	1	0.0000	0.0000	0.0000	0.0003	Zcchc11
0	1	1	1	0	2	4	2	0.0000	0.0003	0.0011	0.0006	Tpm1
1	1	1	0	1	6	2	0	0.0008	0.0008	0.0006	0.0000	Gm9858
1	0	0	0	3	0	0	0	0.0023	0.0000	0.0000	0.0000	Stard13
0	1	3	3	0	1	6	4	0.0000	0.0001	0.0017	0.0011	Pdia2
0	1	0	0	0	1	0	0	0.0000	0.0001	0.0000	0.0000	4930485B16Rik
0	0	0	1	0	0	0	1	0.0000	0.0000	0.0000	0.0003	Gstm5
0	0	0	1	0	0	0	2	0.0000	0.0000	0.0000	0.0006	Ccdc144b
0	1	0	1	0	1	0	2	0.0000	0.0001	0.0000	0.0006	Ryr1
0	0	1	0	0	0	1	0	0.0000	0.0000	0.0003	0.0000	Olfr288
0	3	0	0	0	9	0	0	0.0000	0.0013	0.0000	0.0000	5430421N21Rik
0	3	8	35	0	12	22	74	0.0000	0.0017	0.0062	0.0208	Spna2
0	1	1	3	0	2	3	3	0.0000	0.0003	0.0009	0.0008	Tpm1
0	2	3	19	0	3	5	48	0.0000	0.0004	0.0014	0.0135	Dsp
0	14	11	44	0	46	33	99	0.0000	0.0065	0.0094	0.0279	Ahnak
1	1	1	0	1	1	1	0	0.0008	0.0001	0.0003	0.0000	Urb2
0	1	0	0	0	6	0	0	0.0000	0.0008	0.0000	0.0000	Fcgbp
1	1	0	0	11	3	0	0	0.0085	0.0004	0.0000	0.0000	Upf2
0	1	0	0	0	2	0	0	0.0000	0.0003	0.0000	0.0000	D1Ertd622e
1	1	1	0	5	6	2	0	0.0039	0.0008	0.0006	0.0000	ncharacterized
0	0	1	0	0	0	1	0	0.0000	0.0000	0.0003	0.0000	Hpn
0	0	0	4	0	0	0	6	0.0000	0.0000	0.0000	0.0017	Ahnak2
0	0	0	1	0	0	0	1	0.0000	0.0000	0.0000	0.0003	Sec22b
0	1	1	7	0	14	6	12	0.0000	0.0020	0.0017	0.0034	Dpysl2
0	0	1	0	0	0	1	0	0.0000	0.0000	0.0003	0.0000	Dld
1	2	0	1	2	2	0	2	0.0015	0.0003	0.0000	0.0006	Prkcsh
0	2	1	0	0	5	6	0	0.0000	0.0007	0.0017	0.0000	Prdx4
0	0	0	2	0	0	0	7	0.0000	0.0000	0.0000	0.0020	Ptgis
0	0	0	1	0	0	0	1	0.0000	0.0000	0.0000	0.0003	Dpysl4
0	1	1	0	0	7	2	0	0.0000	0.0010	0.0006	0.0000	Phb2
0	0	1	0	0	0	2	0	0.0000	0.0000	0.0006	0.0000	Srsf5
0	0	0	1	0	0	0	1	0.0000	0.0000	0.0000	0.0003	Capn1
0	3	0	0	0	5	0	0	0.0000	0.0007	0.0000	0.0000	Anp32a
0	0	0	4	0	0	0	7	0.0000	0.0000	0.0000	0.0020	Anxa3
0	0	0	1	0	0	0	1	0.0000	0.0000	0.0000	0.0003	Anxa8
0	0	0	1	0	0	0	1	0.0000	0.0000	0.0000	0.0003	Myadm
0	1	0	0	0	2	0	0	0.0000	0.0003	0.0000	0.0000	Nudc
0	1	0	0	0	2	0	0	0.0000	0.0003	0.0000	0.0000	Bcat2
0	3	3	3	0	7	8	6	0.0000	0.0010	0.0023	0.0017	Calu
0	0	1	8	0	0	6	25	0.0000	0.0000	0.0017	0.0070	Ptrf
0	1	0	2	0	2	0	4	0.0000	0.0003	0.0000	0.0011	Ddost
1	0	0	0	2	0	0	0	0.0015	0.0000	0.0000	0.0000	Aox1
0	0	1	0	0	0	2	0	0.0000	0.0000	0.0006	0.0000	Hmgb3
1	0	0	0	1	0	0	0	0.0008	0.0000	0.0000	0.0000	Banf1
0	1	0	0	0	3	0	0	0.0000	0.0004	0.0000	0.0000	Pgrmc1
0	0	0	1	0	0	0	1	0.0000	0.0000	0.0000	0.0003	Impa1
0	0	0	2	0	0	0	2	0.0000	0.0000	0.0000	0.0006	Entpd2
0	0	0	1	0	0	0	2	0.0000	0.0000	0.0000	0.0006	Copb2
0	0	3	1	0	0	6	1	0.0000	0.0000	0.0017	0.0003	7-Sep
0	0	1	1	0	0	4	2	0.0000	0.0000	0.0011	0.0006	Rpl35a
0	1	2	0	0	4	5	0	0.0000	0.0006	0.0014	0.0000	Psmb5
1	2	0	2	2	12	0	3	0.0015	0.0017	0.0000	0.0008	Eef1b
0	1	0	0	0	1	0	0	0.0000	0.0001	0.0000	0.0000	Epb41l2
0	1	1	0	0	8	2	0	0.0000	0.0011	0.0006	0.0000	Psma3
0	0	1	0	0	0	1	0	0.0000	0.0000	0.0003	0.0000	Sfn
0	2	2	2	0	9	8	4	0.0000	0.0013	0.0023	0.0011	Ugdh
0	0	1	0	0	0	2	0	0.0000	0.0000	0.0006	0.0000	Snx3
0	0	1	0	0	0	2	0	0.0000	0.0000	0.0006	0.0000	Wdr1
0	0	0	1	0	0	0	1	0.0000	0.0000	0.0000	0.0003	Capns1
0	2	0	0	0	5	0	0	0.0000	0.0007	0.0000	0.0000	Dync1i2
7	7	6	7	37	88	28	19	0.0285	0.0124	0.0079	0.0054	Hnrnpa2b1
0	2	2	3	0	4	5	5	0.0000	0.0006	0.0014	0.0014	Idh1
0	0	0	1	0	0	0	1	0.0000	0.0000	0.0000	0.0003	Cyp8b1
1	1	1	0	1	11	4	0	0.0008	0.0015	0.0011	0.0000	Rbm3

0	1	1	0	0	1	4	0	0.0000	0.0001	0.0011	0.0000	Rnase1
0	22	8	11	0	324	68	41	0.0000	0.0456	0.0193	0.0115	Amy2
0	1	0	1	0	1	0	10	0.0000	0.0001	0.0000	0.0028	Krt1
0	0	0	3	0	0	0	6	0.0000	0.0000	0.0000	0.0017	Fabp4
1	9	4	2	2	35	15	4	0.0015	0.0049	0.0043	0.0011	Aldoa
0	0	1	0	0	0	1	0	0.0000	0.0000	0.0003	0.0000	Got2
0	5	2	6	0	58	13	15	0.0000	0.0082	0.0037	0.0042	Cela2a
2	0	0	6	6	0	0	10	0.0046	0.0000	0.0000	0.0028	Tuba1b
1	3	2	3	3	25	5	4	0.0023	0.0035	0.0014	0.0011	Ldha
0	0	2	1	0	0	9	1	0.0000	0.0000	0.0026	0.0003	Gpi
0	2	1	3	0	14	11	5	0.0000	0.0020	0.0031	0.0014	Prss2
2	6	4	14	11	60	40	66	0.0085	0.0084	0.0114	0.0186	Anxa2
0	1	0	4	0	8	0	6	0.0000	0.0011	0.0000	0.0017	Alb
3	4	3	3	10	30	16	8	0.0077	0.0042	0.0045	0.0023	Hsp90aa1
3	5	4	4	6	24	9	6	0.0046	0.0034	0.0026	0.0017	Pdia4
4	10	6	15	10	58	23	43	0.0077	0.0082	0.0065	0.0121	Hsp90b1
0	1	1	2	0	7	7	6	0.0000	0.0010	0.0020	0.0017	S100a10
1	4	3	4	6	34	20	15	0.0046	0.0048	0.0057	0.0042	Sod1
0	9	3	6	0	15	23	11	0.0000	0.0021	0.0065	0.0031	Mdh2
1	0	0	1	3	0	0	1	0.0023	0.0000	0.0000	0.0003	Krt13
0	0	0	1	0	0	0	1	0.0000	0.0000	0.0000	0.0003	Gnai2
0	1	1	1	0	2	1	1	0.0000	0.0003	0.0003	0.0003	Itgb1
8	12	7	14	37	108	44	41	0.0285	0.0152	0.0125	0.0115	P4hb
2	6	6	6	8	29	19	9	0.0062	0.0041	0.0054	0.0025	Ncl
0	1	0	1	0	1	0	1	0.0000	0.0001	0.0000	0.0003	Pgk1
0	2	0	0	0	4	0	0	0.0000	0.0006	0.0000	0.0000	Hmgn2
0	0	4	10	0	0	26	25	0.0000	0.0000	0.0074	0.0070	Anxa1
4	13	6	13	44	109	51	50	0.0340	0.0153	0.0145	0.0141	Eef1a1
1	3	2	4	2	14	7	10	0.0015	0.0020	0.0020	0.0028	Txn
0	2	2	4	0	7	15	10	0.0000	0.0010	0.0043	0.0028	Gstm1
1	2	2	3	5	16	9	9	0.0039	0.0023	0.0026	0.0025	H1f0
1	2	1	0	2	6	5	0	0.0015	0.0008	0.0014	0.0000	Sub1
1	2	2	2	1	13	7	4	0.0008	0.0018	0.0020	0.0011	Hsp90ab1
0	1	1	0	0	1	2	0	0.0000	0.0001	0.0006	0.0000	Itga5
0	2	1	2	0	5	1	2	0.0000	0.0007	0.0003	0.0006	Rpl7a
1	3	4	3	2	20	19	5	0.0015	0.0028	0.0054	0.0014	Gsn
1	1	1	1	6	10	6	4	0.0046	0.0014	0.0017	0.0011	S100a6
0	1	0	1	0	2	0	2	0.0000	0.0003	0.0000	0.0006	Rpl27a
0	2	1	1	0	4	1	1	0.0000	0.0006	0.0003	0.0003	Rps16
0	1	0	1	0	3	0	2	0.0000	0.0004	0.0000	0.0006	Rpl7
1	2	2	4	8	16	8	10	0.0062	0.0023	0.0023	0.0028	Mdh1
2	4	2	4	13	49	13	10	0.0100	0.0069	0.0037	0.0028	Rpsa
2	7	5	9	6	53	21	22	0.0046	0.0075	0.0060	0.0062	Calr
0	1	0	0	0	1	0	0	0.0000	0.0001	0.0000	0.0000	H2-Q9
2	5	5	8	3	21	15	16	0.0023	0.0030	0.0043	0.0045	Lmnb1
1	9	6	10	2	25	18	18	0.0015	0.0035	0.0051	0.0051	Anxa6
1	2	1	4	1	2	6	8	0.0008	0.0003	0.0017	0.0023	Rplp0
0	1	2	3	0	1	3	7	0.0000	0.0001	0.0009	0.0020	Gstm2
4	8	8	4	32	186	72	42	0.0247	0.0262	0.0204	0.0118	Hist1h1c
0	0	1	0	0	0	1	0	0.0000	0.0000	0.0003	0.0000	Klk1
1	6	4	7	7	37	23	40	0.0054	0.0052	0.0065	0.0113	Lgals1
1	4	1	5	2	27	9	10	0.0015	0.0038	0.0026	0.0028	Gapdh
0	0	1	0	0	0	1	0	0.0000	0.0000	0.0003	0.0000	Lamp2
2	7	4	10	14	36	26	22	0.0108	0.0051	0.0074	0.0062	Eno1
1	7	2	6	1	33	18	18	0.0008	0.0046	0.0051	0.0051	Ppia
3	4	5	6	5	45	25	16	0.0039	0.0063	0.0071	0.0045	Tpi1
0	0	0	1	0	0	0	1	0.0000	0.0000	0.0000	0.0003	Lyz1
1	2	1	0	4	8	4	0	0.0031	0.0011	0.0011	0.0000	Pcna
0	0	0	1	0	0	0	2	0.0000	0.0000	0.0000	0.0006	Ctsd
0	0	1	1	0	0	2	2	0.0000	0.0000	0.0006	0.0006	Bsg
2	4	1	6	3	35	16	16	0.0023	0.0049	0.0045	0.0045	Cfl1
0	1	0	0	0	1	0	0	0.0000	0.0001	0.0000	0.0000	Krt19
0	1	0	3	0	1	0	7	0.0000	0.0001	0.0000	0.0020	Gstp1
0	2	1	2	0	4	6	5	0.0000	0.0006	0.0017	0.0014	Rpl13a
2	9	4	7	4	49	20	28	0.0031	0.0069	0.0057	0.0079	Serpinh1
3	13	6	16	6	51	11	44	0.0046	0.0072	0.0031	0.0124	Hspa5
0	0	1	0	0	0	1	0	0.0000	0.0000	0.0003	0.0000	Tmsb4x
0	0	1	0	0	0	2	0	0.0000	0.0000	0.0006	0.0000	Prdx3
1	7	9	25	1	35	45	105	0.0008	0.0049	0.0128	0.0296	Vim
0	0	0	6	0	0	0	12	0.0000	0.0000	0.0000	0.0034	Tgm2
0	3	3	1	0	5	16	2	0.0000	0.0007	0.0045	0.0006	Cbx3

0	1	1	1	0	1	3	1	0.0000	0.0001	0.0009	0.0003	Pnp	
0	1	0	0	0	1	0	0	0.0000	0.0001	0.0000	0.0000	Cat	
1	6	2	6	3	34	33	24	0.0023	0.0048	0.0094	0.0068	Ppib	
1	0	0	0	2	0	0	0	0.0015	0.0000	0.0000	0.0000	Mcm3	
0	1	2	2	0	11	6	2	0.0000	0.0015	0.0017	0.0006	Rps2	
0	1	1	5	0	2	1	9	0.0000	0.0003	0.0003	0.0025	Tln1	
0	1	1	2	0	12	4	2	0.0000	0.0017	0.0011	0.0006	Ezr	
1	5	3	4	3	18	11	7	0.0023	0.0025	0.0031	0.0020	Msn	
0	2	0	0	0	4	0	0	0.0000	0.0006	0.0000	0.0000	Rdx	
3	6	4	3	13	51	11	6	0.0100	0.0072	0.0031	0.0017	Ptma	
0	0	1	0	0	0	1	0	0.0000	0.0000	0.0003	0.0000	Glud1	
0	1	0	0	0	1	0	0	0.0000	0.0001	0.0000	0.0000	Sars	
5	6	4	3	33	68	24	9	0.0255	0.0096	0.0068	0.0025	Marcks	
0	1	4	2	0	2	8	2	0.0000	0.0003	0.0023	0.0006	Map4	
0	1	1	2	0	4	4	2	0.0000	0.0006	0.0011	0.0006	Rpl3	
9	14	10	16	33	148	55	56	0.0255	0.0208	0.0156	0.0158	Pdia3	
0	0	1	0	0	0	3	0	0.0000	0.0000	0.0009	0.0000	Apex1	
1	0	0	0	1	0	0	0	0.0008	0.0000	0.0000	0.0000	Nap1l1	
0	1	0	0	0	1	0	0	0.0000	0.0001	0.0000	0.0000	Nckap1	
2	2	1	0	6	7	2	0	0.0046	0.0010	0.0006	0.0000	Marcksl1	
0	0	0	1	0	0	0	2	0.0000	0.0000	0.0000	0.0006	Dpp4	
1	0	1	0	1	0	2	0	0.0008	0.0000	0.0006	0.0000	Pabpc1	
0	1	2	3	0	5	9	6	0.0000	0.0007	0.0026	0.0017	Oat	
0	1	0	1	0	1	0	3	0.0000	0.0001	0.0000	0.0008	Ppic	
0	1	1	0	0	1	1	0	0.0000	0.0001	0.0003	0.0000	Fkbp4	
1	5	2	1	3	14	7	1	0.0023	0.0020	0.0020	0.0003	Hmgb2	
0	1	4	2	0	18	10	8	0.0000	0.0025	0.0028	0.0023	Des	
0	0	1	0	0	0	1	0	0.0000	0.0000	0.0003	0.0000	Dbi	
2	1	1	1	5	5	1	1	0.0039	0.0007	0.0003	0.0003	Ranbp1	
1	1	0	0	2	2	0	0	0.0015	0.0003	0.0000	0.0000	Mif	
0	0	0	3	0	0	0	4	0.0000	0.0000	0.0000	0.0011	Rab5c	
0	1	0	0	0	1	0	0	0.0000	0.0001	0.0000	0.0000	Pdha1	
0	0	0	1	0	0	0	1	0.0000	0.0000	0.0000	0.0003	Hal	
0	2	0	8	0	8	0	14	0.0000	0.0011	0.0000	0.0039	Canx	
2	5	4	8	4	36	20	25	0.0031	0.0051	0.0057	0.0070	Prdx1	
0	3	2	4	0	21	16	10	0.0000	0.0030	0.0045	0.0028	Rpl12	
0	3	2	3	0	8	12	11	0.0000	0.0011	0.0034	0.0031	Rpl18	
0	1	4	2	0	1	10	4	0.0000	0.0001	0.0028	0.0011	Tagln	
0	0	1	0	0	0	1	0	0.0000	0.0000	0.0003	0.0000	Hmgcl	
0	1	1	1	0	1	4	2	0.0000	0.0001	0.0011	0.0006	Cap1	
1	2	3	3	2	4	7	4	0.0015	0.0006	0.0020	0.0011	Tkt	
0	1	0	4	0	2	0	7	0.0000	0.0003	0.0000	0.0020		2-Sep
0	1	0	1	0	2	0	1	0.0000	0.0003	0.0000	0.0003	Cct8	
0	0	2	3	0	0	3	6	0.0000	0.0000	0.0009	0.0017	Reg1	
0	2	1	2	0	22	7	8	0.0000	0.0031	0.0020	0.0023	Hist1h1e	
3	3	2	3	10	47	24	12	0.0077	0.0066	0.0068	0.0034	Hist1h1a	
3	6	4	4	5	57	43	22	0.0039	0.0080	0.0122	0.0062	Hist1h1b	
0	1	0	0	0	1	0	0	0.0000	0.0001	0.0000	0.0000	Hist1h1d	
0	0	0	1	0	0	0	1	0.0000	0.0000	0.0000	0.0003	Akr1b1	
0	0	0	1	0	0	0	1	0.0000	0.0000	0.0000	0.0003	Cfl2	
0	1	0	0	0	4	0	0	0.0000	0.0006	0.0000	0.0000	Fkbp2	
0	3	2	3	0	21	7	10	0.0000	0.0030	0.0020	0.0028	Aldh2	
0	0	0	1	0	0	0	1	0.0000	0.0000	0.0000	0.0003	Aldh3a2	
0	1	1	1	0	1	2	2	0.0000	0.0001	0.0006	0.0006	Capza1	
0	1	1	1	0	1	1	2	0.0000	0.0001	0.0003	0.0006	Capzb	
0	1	0	0	0	2	0	0	0.0000	0.0003	0.0000	0.0000	Igfbp3	
0	0	1	2	0	0	2	4	0.0000	0.0000	0.0006	0.0011	Rpl6	
0	1	0	0	0	2	0	0	0.0000	0.0003	0.0000	0.0000	Rpl29	
0	1	0	1	0	4	0	2	0.0000	0.0006	0.0000	0.0006	Rplp1	
1	2	2	1	1	7	11	1	0.0008	0.0010	0.0031	0.0003	Rpl5	
0	3	2	1	0	12	7	2	0.0000	0.0017	0.0020	0.0006	Rpl13	
0	2	1	0	0	4	2	0	0.0000	0.0006	0.0006	0.0000	Rpl36	
1	7	5	11	2	53	28	36	0.0015	0.0075	0.0079	0.0101	Anxa5	
3	14	11	9	4	62	38	14	0.0031	0.0087	0.0108	0.0039	Lmna	
0	1	0	0	0	3	0	0	0.0000	0.0004	0.0000	0.0000	Brca1	
0	1	0	0	0	3	0	0	0.0000	0.0004	0.0000	0.0000	Cbr1	
0	1	0	0	0	2	0	0	0.0000	0.0003	0.0000	0.0000	Cox7a2	
1	1	0	0	9	7	0	0	0.0069	0.0010	0.0000	0.0000	Dnase1	
4	6	3	3	27	32	11	5	0.0208	0.0045	0.0031	0.0014	Hnrnpa1	
0	1	0	0	0	1	0	0	0.0000	0.0001	0.0000	0.0000	Psma2	
0	0	1	2	0	0	2	9	0.0000	0.0000	0.0006	0.0025	Cav1	

0	1	0	0	0	4	0	0	0.0000	0.0006	0.0000	0.0000	Cdkn1c
1	4	3	7	1	15	14	18	0.0008	0.0021	0.0040	0.0051	Ahcy
0	1	0	1	0	3	0	4	0.0000	0.0004	0.0000	0.0011	S100a11
2	3	4	0	5	5	9	0	0.0039	0.0007	0.0026	0.0000	Pa2g4
0	1	1	2	0	1	4	3	0.0000	0.0001	0.0011	0.0008	Rab7a
0	0	1	1	0	0	1	1	0.0000	0.0000	0.0003	0.0003	Acadl
0	0	0	2	0	0	0	3	0.0000	0.0000	0.0000	0.0008	Rpl9
0	0	1	0	0	0	3	0	0.0000	0.0000	0.0009	0.0000	Hsd17b4
0	3	1	1	0	14	4	1	0.0000	0.0020	0.0011	0.0003	Hdgf
3	10	3	10	5	62	14	21	0.0039	0.0087	0.0040	0.0059	Pkm2
0	1	0	0	0	1	0	0	0.0000	0.0001	0.0000	0.0000	Efna1
1	1	0	0	1	1	0	0	0.0008	0.0001	0.0000	0.0000	Hmga2
0	1	1	1	0	1	2	1	0.0000	0.0001	0.0006	0.0003	Rpl10a
0	3	4	6	0	13	15	12	0.0000	0.0018	0.0043	0.0034	Idh2
1	3	0	0	2	3	0	0	0.0015	0.0004	0.0000	0.0000	Stmn1
0	0	0	1	0	0	0	1	0.0000	0.0000	0.0000	0.0003	Cyb5a
4	6	3	9	21	22	9	17	0.0162	0.0031	0.0026	0.0048	Atp5b
1	1	0	1	2	1	0	1	0.0015	0.0001	0.0000	0.0003	Ucp3
1	3	3	2	2	13	13	4	0.0015	0.0018	0.0037	0.0011	Fus
0	1	0	0	0	2	0	0	0.0000	0.0003	0.0000	0.0000	Erp29
0	1	1	2	0	1	7	3	0.0000	0.0001	0.0020	0.0008	Eef1d
0	1	1	5	0	2	3	6	0.0000	0.0003	0.0009	0.0017	Actn4
1	11	7	11	4	35	31	19	0.0031	0.0049	0.0088	0.0054	Eef2
1	2	2	2	2	14	12	3	0.0015	0.0020	0.0034	0.0008	Tpm1
0	0	0	1	0	0	0	1	0.0000	0.0000	0.0000	0.0003	Tnks1bp1
0	1	0	0	0	1	0	0	0.0000	0.0001	0.0000	0.0000	Slc12a3
0	0	1	0	0	0	1	0	0.0000	0.0000	0.0003	0.0000	Ruvbl1
0	1	1	1	0	13	5	2	0.0000	0.0018	0.0014	0.0006	Pcbp1
0	2	1	0	0	3	3	0	0.0000	0.0004	0.0009	0.0000	Cdc42
0	0	0	1	0	0	0	1	0.0000	0.0000	0.0000	0.0003	Cirbp
1	4	1	2	2	9	3	5	0.0015	0.0013	0.0009	0.0014	Eif4a1
1	2	2	2	1	17	11	10	0.0008	0.0024	0.0031	0.0028	Rps20
1	2	1	2	4	18	5	2	0.0031	0.0025	0.0014	0.0006	Ube2n
1	1	0	0	3	5	0	0	0.0023	0.0007	0.0000	0.0000	Drd2
2	2	1	0	5	11	1	0	0.0039	0.0015	0.0003	0.0000	Rpl26
0	1	1	1	0	2	4	2	0.0000	0.0003	0.0011	0.0006	Rpl27
1	1	1	1	1	13	4	2	0.0008	0.0018	0.0011	0.0006	Arf4
0	1	0	2	0	1	0	4	0.0000	0.0001	0.0000	0.0011	Dad1
0	1	2	0	0	17	10	0	0.0000	0.0024	0.0028	0.0000	Sumo2
4	12	8	10	21	84	36	23	0.0162	0.0118	0.0102	0.0065	Hnrnpk
0	1	2	3	0	6	8	7	0.0000	0.0008	0.0023	0.0020	Ywhag
0	0	0	1	0	0	0	2	0.0000	0.0000	0.0000	0.0006	Rras2
0	2	0	3	0	4	0	4	0.0000	0.0006	0.0000	0.0011	Rps7
0	1	0	0	0	7	0	0	0.0000	0.0010	0.0000	0.0000	Ppp1ca
0	1	0	0	0	3	0	0	0.0000	0.0004	0.0000	0.0000	Ppp1cb
0	2	1	2	0	4	2	4	0.0000	0.0006	0.0006	0.0011	Calm1
1	2	2	3	2	8	8	8	0.0015	0.0011	0.0023	0.0023	Rps8
0	1	1	2	0	5	2	3	0.0000	0.0007	0.0006	0.0008	Rps15a
3	7	2	3	9	32	7	12	0.0069	0.0045	0.0020	0.0034	Ywhae
1	2	2	1	1	20	10	2	0.0008	0.0028	0.0028	0.0006	Rps14
2	4	4	1	8	22	13	1	0.0062	0.0031	0.0037	0.0003	Rps18
0	0	1	0	0	0	1	0	0.0000	0.0000	0.0003	0.0000	Rps11
0	1	0	0	0	3	0	0	0.0000	0.0004	0.0000	0.0000	Rps13
0	2	0	1	0	4	0	1	0.0000	0.0006	0.0000	0.0003	Snrpe
0	2	1	0	0	8	4	0	0.0000	0.0011	0.0011	0.0000	Snrpf
1	0	0	0	1	0	0	0	0.0008	0.0000	0.0000	0.0000	Snrpg
0	1	0	1	0	1	0	2	0.0000	0.0001	0.0000	0.0006	Snrpd1
0	1	2	0	0	10	3	0	0.0000	0.0014	0.0009	0.0000	Snrpd2
0	1	0	0	0	2	0	0	0.0000	0.0003	0.0000	0.0000	Snrpd3
0	2	3	2	0	3	6	3	0.0000	0.0004	0.0017	0.0008	Rab11a
0	1	1	0	0	2	4	0	0.0000	0.0003	0.0011	0.0000	Tsc22d1
1	3	1	2	1	8	2	6	0.0008	0.0011	0.0006	0.0017	Rps4x
0	0	1	0	0	0	4	0	0.0000	0.0000	0.0011	0.0000	Rpl18a
1	3	4	2	3	8	13	4	0.0023	0.0011	0.0037	0.0011	Rpl23a
0	2	0	0	0	4	0	0	0.0000	0.0006	0.0000	0.0000	Rps6
5	7	5	6	91	206	83	67	0.0702	0.0290	0.0236	0.0189	Hist1h4a
0	0	0	1	0	0	0	2	0.0000	0.0000	0.0000	0.0006	Rab1A
2	3	2	2	8	19	9	7	0.0062	0.0027	0.0026	0.0020	Ran
0	0	0	2	0	0	0	6	0.0000	0.0000	0.0000	0.0017	Rpl23
0	1	1	1	0	11	6	1	0.0000	0.0015	0.0017	0.0003	Rps15
1	0	0	1	1	0	0	2	0.0008	0.0000	0.0000	0.0006	Rps24

0	2	1	2	0	10	7	3	0.0000	0.0014	0.0020	0.0008	Rps25
0	2	1	1	0	10	6	2	0.0000	0.0014	0.0017	0.0006	Rps26
0	2	1	1	0	13	4	3	0.0000	0.0018	0.0011	0.0008	Rps28
0	1	0	0	0	8	0	0	0.0000	0.0011	0.0000	0.0000	Tceb2
0	1	0	1	0	1	0	1	0.0000	0.0001	0.0000	0.0003	Gnb2
0	1	0	2	0	3	0	3	0.0000	0.0004	0.0000	0.0008	Rpl30
1	1	1	1	2	3	3	3	0.0015	0.0004	0.0009	0.0008	Rpl31
1	1	1	3	2	1	7	7	0.0015	0.0001	0.0020	0.0020	Rps3
0	0	1	1	0	0	4	2	0.0000	0.0000	0.0011	0.0006	Rpl32
2	2	2	2	12	10	8	2	0.0093	0.0014	0.0023	0.0006	Rpl8
0	2	1	2	0	11	6	5	0.0000	0.0015	0.0017	0.0014	Ybx1
1	5	1	5	1	19	1	12	0.0008	0.0027	0.0003	0.0034	Pfn1
0	1	0	0	0	1	0	0	0.0000	0.0001	0.0000	0.0000	Crabp1
2	2	2	2	3	19	6	6	0.0023	0.0027	0.0017	0.0017	Uba52
0	0	0	1	0	0	0	1	0.0000	0.0000	0.0000	0.0003	Tra2b
0	0	1	0	0	0	2	0	0.0000	0.0000	0.0006	0.0000	Pafah1b1
3	19	6	14	13	77	31	38	0.0100	0.0108	0.0088	0.0107	Hspa8
0	0	0	1	0	0	0	2	0.0000	0.0000	0.0000	0.0006	Tpt1
3	9	6	3	18	58	26	8	0.0139	0.0082	0.0074	0.0023	Hspd1
0	1	0	1	0	7	0	1	0.0000	0.0010	0.0000	0.0003	Mapk1
5	11	3	9	14	87	19	29	0.0108	0.0122	0.0054	0.0082	Ywhaz
4	10	7	4	13	36	47	24	0.0100	0.0051	0.0133	0.0068	Hmgb1
1	0	0	0	1	0	0	0	0.0008	0.0000	0.0000	0.0000	Snrpn
1	2	1	4	8	7	2	8	0.0062	0.0010	0.0006	0.0023	Eif5a
0	1	1	1	0	5	6	4	0.0000	0.0007	0.0017	0.0011	Crip1
2	12	5	12	8	99	67	86	0.0062	0.0139	0.0190	0.0242	Actg1
0	1	0	1	0	2	0	1	0.0000	0.0003	0.0000	0.0003	Rps17
0	1	1	1	0	7	2	1	0.0000	0.0010	0.0006	0.0003	Rps12
1	1	1	0	7	2	1	0	0.0054	0.0003	0.0003	0.0000	Rps10
0	0	1	0	0	0	1	0	0.0000	0.0000	0.0003	0.0000	Phb
0	0	1	0	0	0	2	0	0.0000	0.0000	0.0006	0.0000	Csnk2b
0	2	2	2	0	6	16	5	0.0000	0.0008	0.0045	0.0014	Rpl22
0	1	1	0	0	2	2	0	0.0000	0.0003	0.0006	0.0000	Ube2l3
0	0	1	1	0	0	3	2	0.0000	0.0000	0.0009	0.0006	Gnb2l1
3	3	4	5	62	90	42	18	0.0478	0.0127	0.0119	0.0051	Acta1
2	2	1	4	4	21	8	13	0.0031	0.0030	0.0023	0.0037	Ywhaq
1	4	1	4	1	9	9	11	0.0008	0.0013	0.0026	0.0031	Tuba1a
0	0	0	2	0	0	0	4	0.0000	0.0000	0.0000	0.0011	Tubb2c
2	2	1	1	4	3	4	2	0.0031	0.0004	0.0011	0.0006	Hist1h3a
1	3	2	4	1	19	6	5	0.0008	0.0027	0.0017	0.0014	Ywhah
0	1	0	0	0	2	0	0	0.0000	0.0003	0.0000	0.0000	Zhx1
0	0	1	3	0	0	1	5	0.0000	0.0000	0.0003	0.0014	Kpnb1
0	2	1	0	0	3	3	0	0.0000	0.0004	0.0009	0.0000	Psmb7
0	1	0	3	0	7	0	6	0.0000	0.0010	0.0000	0.0017	Pebp1
0	2	1	2	0	10	10	8	0.0000	0.0014	0.0028	0.0023	Hnrnph2
0	0	1	1	0	0	8	4	0.0000	0.0000	0.0023	0.0011	Hint1
0	1	1	0	0	9	3	0	0.0000	0.0013	0.0009	0.0000	Elavl1
0	0	1	2	0	0	4	5	0.0000	0.0000	0.0011	0.0014	Slc9a3r1
0	0	1	0	0	0	1	0	0.0000	0.0000	0.0003	0.0000	Cct7
0	1	0	1	0	1	0	1	0.0000	0.0001	0.0000	0.0003	Cct4
0	0	0	1	0	0	0	1	0.0000	0.0000	0.0000	0.0003	Cct5
0	1	1	1	0	3	3	2	0.0000	0.0004	0.0009	0.0006	Cbx1
0	4	1	4	0	18	2	9	0.0000	0.0025	0.0006	0.0025	Arf1
1	1	1	2	4	6	3	4	0.0031	0.0008	0.0009	0.0011	Rpl19
1	1	0	1	2	2	0	2	0.0015	0.0003	0.0000	0.0006	Srsf3
1	1	0	1	11	2	0	5	0.0085	0.0003	0.0000	0.0014	Hist1h3b
1	1	0	1	2	2	0	2	0.0015	0.0003	0.0000	0.0006	H3f3a
0	0	1	1	0	0	1	1	0.0000	0.0000	0.0003	0.0003	Nptn
0	0	1	0	0	0	1	0	0.0000	0.0000	0.0003	0.0000	Mcm2
0	0	1	0	0	0	1	0	0.0000	0.0000	0.0003	0.0000	Csrp1
0	0	0	1	0	0	0	2	0.0000	0.0000	0.0000	0.0006	Pkp1
0	2	3	1	0	2	4	2	0.0000	0.0003	0.0011	0.0006	Rps3a
0	0	1	1	0	0	2	2	0.0000	0.0000	0.0006	0.0006	S100a13
1	0	1	1	2	0	1	5	0.0015	0.0000	0.0003	0.0014	Psme1
0	0	0	1	0	0	0	1	0.0000	0.0000	0.0000	0.0003	Psme2
0	1	2	4	0	1	11	9	0.0000	0.0001	0.0031	0.0025	Anxa4
0	0	1	1	0	0	3	1	0.0000	0.0000	0.0009	0.0003	Fhl1
0	0	1	2	0	0	2	3	0.0000	0.0000	0.0006	0.0008	Anpep
0	1	0	0	0	2	0	0	0.0000	0.0003	0.0000	0.0000	Atp5j
0	1	0	0	0	1	0	0	0.0000	0.0001	0.0000	0.0000	Shbg
0	1	0	2	0	2	0	2	0.0000	0.0003	0.0000	0.0006	Fh

0	0	0	2	0	0	0	3	0.0000	0.0000	0.0000	0.0008	G3bp1
0	1	2	8	0	6	9	26	0.0000	0.0008	0.0026	0.0073	Tubb5
0	2	0	0	0	2	0	0	0.0000	0.0003	0.0000	0.0000	Psmb4
3	2	1	2	6	18	3	5	0.0046	0.0025	0.0009	0.0014	Rplp2
0	1	0	3	0	2	0	5	0.0000	0.0003	0.0000	0.0014	Prdx5
0	0	0	1	0	0	0	1	0.0000	0.0000	0.0000	0.0003	Xdh
0	1	1	3	0	2	1	8	0.0000	0.0003	0.0003	0.0023	Rbp1
0	0	1	0	0	0	4	0	0.0000	0.0000	0.0011	0.0000	Hnrnpul2
0	0	0	1	0	0	0	2	0.0000	0.0000	0.0000	0.0006	Rsu1
1	5	4	6	2	28	35	14	0.0015	0.0039	0.0099	0.0039	Nme2
1	4	0	5	1	12	0	11	0.0008	0.0017	0.0000	0.0031	Vcp
1	3	3	8	1	15	9	11	0.0008	0.0021	0.0026	0.0031	Uba1
0	0	0	8	0	0	0	18	0.0000	0.0000	0.0000	0.0051	Jup
1	3	1	5	2	6	10	9	0.0015	0.0008	0.0028	0.0025	Atp5a1
0	0	0	2	0	0	0	2	0.0000	0.0000	0.0000	0.0006	Ckb
0	0	1	1	0	0	2	1	0.0000	0.0000	0.0006	0.0003	Col6a1
0	0	1	1	0	0	4	2	0.0000	0.0000	0.0011	0.0006	Rac2
1	2	1	2	1	6	1	6	0.0008	0.0008	0.0003	0.0017	Rcn1
1	2	2	3	2	13	13	4	0.0015	0.0018	0.0037	0.0011	Fabp5
0	1	1	0	0	2	5	0	0.0000	0.0003	0.0014	0.0000	Atp5i
1	8	4	0	2	16	12	0	0.0015	0.0023	0.0034	0.0000	Igf2r
0	0	1	0	0	0	2	0	0.0000	0.0000	0.0006	0.0000	P311
0	0	0	1	0	0	0	2	0.0000	0.0000	0.0000	0.0006	Tgm3
0	1	0	0	0	2	0	0	0.0000	0.0003	0.0000	0.0000	Dlk1
0	0	0	2	0	0	0	3	0.0000	0.0000	0.0000	0.0008	Krt80
0	0	0	1	0	0	0	4	0.0000	0.0000	0.0000	0.0011	Flg2
0	0	0	1	0	0	0	2	0.0000	0.0000	0.0000	0.0006	Hp1bp3
0	3	3	3	0	14	11	10	0.0000	0.0020	0.0031	0.0028	Myl12b
1	1	0	3	2	1	0	4	0.0015	0.0001	0.0000	0.0011	H2afv
0	1	0	0	0	2	0	0	0.0000	0.0003	0.0000	0.0000	Llgl2
1	7	2	5	2	29	9	14	0.0015	0.0041	0.0026	0.0039	Pdia6
0	1	1	0	0	2	4	0	0.0000	0.0003	0.0011	0.0000	Srsf6
1	0	0	0	1	0	0	0	0.0008	0.0000	0.0000	0.0000	Mphosph8
0	3	2	1	0	11	3	1	0.0000	0.0015	0.0009	0.0003	Khsrp
0	0	1	0	0	0	1	0	0.0000	0.0000	0.0003	0.0000	Ddb1
0	0	0	1	0	0	0	2	0.0000	0.0000	0.0000	0.0006	Esyt1
0	1	0	0	0	2	0	0	0.0000	0.0003	0.0000	0.0000	Agap2
0	0	0	1	0	0	0	1	0.0000	0.0000	0.0000	0.0003	Pdap1
1	0	0	0	1	0	0	0	0.0008	0.0000	0.0000	0.0000	Sf3b2
0	1	0	0	0	1	0	0	0.0000	0.0001	0.0000	0.0000	Plcl1
0	0	0	2	0	0	0	3	0.0000	0.0000	0.0000	0.0008	Fam129a
0	0	1	0	0	0	1	0	0.0000	0.0000	0.0003	0.0000	Gm1661
0	0	0	1	0	0	0	2	0.0000	0.0000	0.0000	0.0006	Dnah12
0	0	0	1	0	0	0	1	0.0000	0.0000	0.0000	0.0003	Lepre1
1	0	0	0	1	0	0	0	0.0008	0.0000	0.0000	0.0000	Psg28
0	0	0	1	0	0	0	2	0.0000	0.0000	0.0000	0.0006	Xirp2
0	1	0	0	0	2	0	0	0.0000	0.0003	0.0000	0.0000	Ppp3ca
1	1	0	2	5	9	0	3	0.0039	0.0013	0.0000	0.0008	Ddx17
0	8	1	0	0	26	3	0	0.0000	0.0037	0.0009	0.0000	Cpa2
0	0	0	1	0	0	0	2	0.0000	0.0000	0.0000	0.0006	DXBay18
0	5	5	7	0	32	18	12	0.0000	0.0045	0.0051	0.0034	Tpm3
1	20	8	12	10	287	55	38	0.0077	0.0404	0.0156	0.0107	Pnliprp1
0	1	0	0	0	1	0	0	0.0000	0.0001	0.0000	0.0000	Rgag4
0	1	0	0	0	1	0	0	0.0000	0.0001	0.0000	0.0000	Trpm3
0	0	0	1	0	0	0	1	0.0000	0.0000	0.0000	0.0003	Arcn1
0	1	2	3	0	6	16	9	0.0000	0.0008	0.0045	0.0025	Myl6
1	1	0	1	3	16	0	2	0.0023	0.0023	0.0000	0.0006	Akap4
0	2	1	2	0	2	2	5	0.0000	0.0003	0.0006	0.0014	Hnrnpd
0	2	1	1	0	16	9	2	0.0000	0.0023	0.0026	0.0006	Psmb6
0	0	0	3	0	0	0	5	0.0000	0.0000	0.0000	0.0014	P4ha1
0	1	1	0	0	6	2	0	0.0000	0.0008	0.0006	0.0000	P4ha2
0	1	1	1	0	1	6	2	0.0000	0.0001	0.0017	0.0006	Bag1
0	1	0	0	0	2	0	0	0.0000	0.0003	0.0000	0.0000	Igf1r
0	2	0	1	0	3	0	2	0.0000	0.0004	0.0000	0.0006	Naca
0	0	0	2	0	0	0	4	0.0000	0.0000	0.0000	0.0011	Serpinb6
0	1	1	0	0	1	1	0	0.0000	0.0001	0.0003	0.0000	Stip1
0	0	0	1	0	0	0	1	0.0000	0.0000	0.0000	0.0003	Caprin1
0	0	1	0	0	0	1	0	0.0000	0.0000	0.0003	0.0000	Reep5
0	0	0	1	0	0	0	1	0.0000	0.0000	0.0000	0.0003	Vdac3
0	2	3	2	0	13	5	4	0.0000	0.0018	0.0014	0.0011	Vdac1
0	0	2	0	0	0	3	0	0.0000	0.0000	0.0009	0.0000	Dmbt1

0	1	2	2	0	1	5	3	0.0000	0.0001	0.0014	0.0008	Tmpo
0	1	1	0	0	2	1	0	0.0000	0.0003	0.0003	0.0000	Hars
1	5	1	4	3	14	4	7	0.0023	0.0020	0.0011	0.0020	Prdx2
0	1	1	0	0	13	1	0	0.0000	0.0018	0.0003	0.0000	Psap
0	1	0	0	0	2	0	0	0.0000	0.0003	0.0000	0.0000	Pde3b
0	2	0	0	0	6	0	0	0.0000	0.0008	0.0000	0.0000	Hadh
0	2	0	0	0	8	0	0	0.0000	0.0011	0.0000	0.0000	Ewsr1
0	3	3	2	0	5	9	2	0.0000	0.0007	0.0026	0.0006	Fscn1
0	0	0	1	0	0	0	1	0.0000	0.0000	0.0000	0.0003	Gdi2
0	0	0	1	0	0	0	2	0.0000	0.0000	0.0000	0.0006	Arhgdib
0	1	2	0	0	1	4	0	0.0000	0.0001	0.0011	0.0000	Phgdh
0	0	0	1	0	0	0	2	0.0000	0.0000	0.0000	0.0006	Lasp1
0	0	0	1	0	0	0	2	0.0000	0.0000	0.0000	0.0006	Myh10
2	4	2	4	11	33	15	10	0.0085	0.0046	0.0043	0.0028	Npm1
0	0	0	2	0	0	0	3	0.0000	0.0000	0.0000	0.0008	Pcbp2
0	1	1	1	0	3	6	2	0.0000	0.0004	0.0017	0.0006	Pea15
0	1	0	1	0	9	0	2	0.0000	0.0013	0.0000	0.0006	Srsf2
0	1	0	1	0	1	0	2	0.0000	0.0001	0.0000	0.0006	Aldh1a2
1	0	1	0	1	0	2	0	0.0008	0.0000	0.0006	0.0000	Ddx3x
0	1	1	2	0	2	4	3	0.0000	0.0003	0.0011	0.0008	Dpysl3
0	1	1	0	0	3	1	0	0.0000	0.0004	0.0003	0.0000	Snrpa
0	1	8	27	0	1	23	57	0.0000	0.0001	0.0065	0.0161	Sptbn1
0	1	0	0	0	2	0	0	0.0000	0.0003	0.0000	0.0000	Trim28
0	1	0	0	0	1	0	0	0.0000	0.0001	0.0000	0.0000	Snrnp70
0	1	0	0	0	10	0	0	0.0000	0.0014	0.0000	0.0000	Fkbp3
0	1	2	3	0	3	6	6	0.0000	0.0004	0.0017	0.0017	Vat1
0	1	1	1	0	14	6	2	0.0000	0.0020	0.0017	0.0006	Selenbp2
0	0	0	1	0	0	0	1	0.0000	0.0000	0.0000	0.0003	Sdpr
1	0	1	0	1	0	1	0	0.0008	0.0000	0.0003	0.0000	Raly
0	1	0	0	0	1	0	0	0.0000	0.0001	0.0000	0.0000	Ccpg1
0	6	1	4	0	25	3	9	0.0000	0.0035	0.0009	0.0025	Cel
0	1	0	0	0	2	0	0	0.0000	0.0003	0.0000	0.0000	Cd34
1	3	2	2	10	26	17	4	0.0077	0.0037	0.0048	0.0011	Hspe1
2	2	0	3	30	42	0	14	0.0231	0.0059	0.0000	0.0039	Hist2h2ab
0	1	4	8	0	4	7	14	0.0000	0.0006	0.0020	0.0039	Vcl
0	0	0	2	0	0	0	2	0.0000	0.0000	0.0000	0.0006	Cltc
0	1	0	0	0	3	0	0	0.0000	0.0004	0.0000	0.0000	Morc3
0	0	0	1	0	0	0	1	0.0000	0.0000	0.0000	0.0003	Nlrp14
1	1	0	1	2	2	0	1	0.0015	0.0003	0.0000	0.0003	Gm9839
1	0	0	3	1	0	0	5	0.0008	0.0000	0.0000	0.0014	Krt77
0	6	4	6	0	34	16	10	0.0000	0.0048	0.0045	0.0028	Tpm4
0	0	1	0	0	0	1	0	0.0000	0.0000	0.0003	0.0000	Sri
0	0	0	1	0	0	0	1	0.0000	0.0000	0.0000	0.0003	Mapk4
0	1	0	1	0	5	0	1	0.0000	0.0007	0.0000	0.0003	Trak2
0	1	0	0	0	1	0	0	0.0000	0.0001	0.0000	0.0000	Smarcc2
2	4	1	0	7	4	6	0	0.0054	0.0006	0.0017	0.0000	Srsf1
0	0	1	0	0	0	1	0	0.0000	0.0000	0.0003	0.0000	Mylk
0	0	1	0	0	0	2	0	0.0000	0.0000	0.0006	0.0000	Rcor3
0	0	1	0	0	0	1	0	0.0000	0.0000	0.0003	0.0000	Rbm15b
0	1	0	1	0	2	0	2	0.0000	0.0003	0.0000	0.0006	Tpm2
0	1	0	1	0	3	0	2	0.0000	0.0004	0.0000	0.0006	Ubr2
0	2	1	1	0	3	7	2	0.0000	0.0004	0.0020	0.0006	Kctd12
1	1	0	0	1	1	0	0	0.0008	0.0001	0.0000	0.0000	Kdm3b
0	1	1	1	0	6	2	1	0.0000	0.0008	0.0006	0.0003	Rps9
0	0	1	0	0	0	2	0	0.0000	0.0000	0.0006	0.0000	Rps27
0	0	0	2	0	0	0	3	0.0000	0.0000	0.0000	0.0008	Rpl10
1	1	1	1	5	17	3	2	0.0039	0.0024	0.0009	0.0006	Rpl35
0	0	1	0	0	0	1	0	0.0000	0.0000	0.0003	0.0000	Eif2s1
0	3	3	0	0	4	8	0	0.0000	0.0006	0.0023	0.0000	Snd1
0	1	1	0	0	6	7	0	0.0000	0.0008	0.0020	0.0000	Gm5771
0	3	2	0	0	22	6	0	0.0000	0.0031	0.0017	0.0000	Prss3
0	3	1	1	0	7	5	4	0.0000	0.0010	0.0014	0.0011	Try10
0	2	1	3	0	5	6	6	0.0000	0.0007	0.0017	0.0017	Syncrip
0	1	0	0	0	5	0	0	0.0000	0.0007	0.0000	0.0000	Luc7l2
1	0	0	3	1	0	0	5	0.0008	0.0000	0.0000	0.0014	Actn1
1	14	6	7	1	158	39	25	0.0008	0.0222	0.0111	0.0070	Cpa1
1	0	0	0	3	0	0	0	0.0023	0.0000	0.0000	0.0000	Nav3
0	0	0	1	0	0	0	1	0.0000	0.0000	0.0000	0.0003	Znf579
0	0	1	0	0	0	2	0	0.0000	0.0000	0.0006	0.0000	Mtdh
0	1	1	0	0	2	1	0	0.0000	0.0003	0.0003	0.0000	Jph4
0	0	1	4	0	0	1	7	0.0000	0.0000	0.0003	0.0020	Flnb

0	1	0	0	0	2	0	0	0.0000	0.0003	0.0000	0.0000	BC051142
0	1	0	0	0	3	0	0	0.0000	0.0004	0.0000	0.0000	Nucks1
0	0	1	0	0	0	2	0	0.0000	0.0000	0.0006	0.0000	Tufm
0	0	1	0	0	0	1	0	0.0000	0.0000	0.0003	0.0000	Cpped1
2	4	4	6	24	36	21	20	0.0185	0.0051	0.0060	0.0056	Hnrnpa3
1	0	0	0	1	0	0	0	0.0008	0.0000	0.0000	0.0000	Eif4b
0	0	0	1	0	0	0	1	0.0000	0.0000	0.0000	0.0003	Mak16
0	0	0	3	0	0	0	7	0.0000	0.0000	0.0000	0.0020	Ehd2
0	0	1	0	0	0	3	0	0.0000	0.0000	0.0009	0.0000	Echs1
0	2	1	4	0	4	3	7	0.0000	0.0006	0.0009	0.0020	Rcn3
0	1	0	0	0	1	0	0	0.0000	0.0001	0.0000	0.0000	Ganab
0	1	0	0	0	1	0	0	0.0000	0.0001	0.0000	0.0000	Sgta
0	1	0	0	0	1	0	0	0.0000	0.0001	0.0000	0.0000	Rcc2
0	2	1	0	0	4	2	0	0.0000	0.0006	0.0006	0.0000	Srsf7
2	8	6	9	6	38	22	23	0.0046	0.0053	0.0062	0.0065	Ckap4
0	0	3	2	0	0	5	4	0.0000	0.0000	0.0014	0.0011	Hadha
0	0	1	1	0	0	1	2	0.0000	0.0000	0.0003	0.0006	Rpl24
0	0	0	1	0	0	0	1	0.0000	0.0000	0.0000	0.0003	Rcn2
0	0	0	1	0	0	0	2	0.0000	0.0000	0.0000	0.0006	Asph
0	0	1	0	0	0	1	0	0.0000	0.0000	0.0003	0.0000	Lig4
0	10	12	6	0	30	36	9	0.0000	0.0042	0.0102	0.0025	Flna
0	0	1	1	0	0	2	2	0.0000	0.0000	0.0006	0.0006	Aifm2
0	0	0	4	0	0	0	6	0.0000	0.0000	0.0000	0.0017	Acaa2
0	1	0	0	0	1	0	0	0.0000	0.0001	0.0000	0.0000	Pxk
0	0	0	1	0	0	0	1	0.0000	0.0000	0.0000	0.0003	Asrgl1
0	0	0	1	0	0	0	2	0.0000	0.0000	0.0000	0.0006	ncharacterized
0	3	1	1	0	10	2	1	0.0000	0.0014	0.0006	0.0003	11-Sep
0	0	1	0	0	0	1	0	0.0000	0.0000	0.0003	0.0000	Gykl1
1	0	0	0	1	0	0	0	0.0008	0.0000	0.0000	0.0000	Tceal5
0	1	2	0	0	3	6	0	0.0000	0.0004	0.0017	0.0000	Txnl1
0	1	0	0	0	1	0	0	0.0000	0.0001	0.0000	0.0000	Qrfp
4	6	5	6	117	171	37	52	0.0903	0.0241	0.0105	0.0146	Hist1h2bp
0	0	0	1	0	0	0	1	0.0000	0.0000	0.0000	0.0003	Copa
0	0	0	1	0	0	0	2	0.0000	0.0000	0.0000	0.0006	Fgb
0	1	1	1	0	6	2	2	0.0000	0.0008	0.0006	0.0006	Tpm3
1	1	1	1	3	3	6	2	0.0023	0.0004	0.0017	0.0006	Sdha
1	1	0	0	1	1	0	0	0.0008	0.0001	0.0000	0.0000	P2rx2
0	0	1	0	0	0	2	0	0.0000	0.0000	0.0006	0.0000	Pof1b
0	0	0	1	0	0	0	1	0.0000	0.0000	0.0000	0.0003	Lgi2
0	2	0	3	0	2	0	6	0.0000	0.0003	0.0000	0.0017	Acat1
0	1	0	0	0	1	0	0	0.0000	0.0001	0.0000	0.0000	Gspt1
0	4	0	0	0	7	0	0	0.0000	0.0010	0.0000	0.0000	Hnrnpl
1	1	0	1	11	36	0	16	0.0085	0.0051	0.0000	0.0045	H2afj
0	1	1	1	0	3	6	6	0.0000	0.0004	0.0017	0.0017	Ssspta
0	0	1	0	0	0	2	0	0.0000	0.0000	0.0006	0.0000	Ushbp1
0	1	1	0	0	1	2	0	0.0000	0.0001	0.0006	0.0000	Actr1b
1	0	0	0	1	0	0	0	0.0008	0.0000	0.0000	0.0000	Sgms1
0	3	1	3	0	9	4	6	0.0000	0.0013	0.0011	0.0017	Cald1
0	0	3	15	0	0	7	26	0.0000	0.0000	0.0020	0.0073	Myh9
0	0	0	1	0	0	0	1	0.0000	0.0000	0.0000	0.0003	Atp1a1
0	0	0	1	0	0	0	1	0.0000	0.0000	0.0000	0.0003	rotein
1	1	1	1	1	4	5	1	0.0008	0.0006	0.0014	0.0003	Srsf4
1	3	3	5	2	15	11	9	0.0015	0.0021	0.0031	0.0025	Hnrnpu
0	0	1	0	0	0	1	0	0.0000	0.0000	0.0003	0.0000	Olfr1317
0	1	0	0	0	1	0	0	0.0000	0.0001	0.0000	0.0000	Olfr516
0	2	1	2	0	3	2	2	0.0000	0.0004	0.0006	0.0006	Hnrnpr
1	3	3	1	4	18	14	5	0.0031	0.0025	0.0040	0.0014	Sfpq
0	1	0	0	0	2	0	0	0.0000	0.0003	0.0000	0.0000	Slc12a5
0	1	0	0	0	2	0	0	0.0000	0.0003	0.0000	0.0000	Rab14
0	0	0	1	0	0	0	3	0.0000	0.0000	0.0000	0.0008	Rps5
0	0	0	1	0	0	0	1	0.0000	0.0000	0.0000	0.0003	Isoc1
1	1	2	3	3	16	13	16	0.0023	0.0023	0.0037	0.0045	Hba-a1
0	0	4	1	0	0	7	2	0.0000	0.0000	0.0020	0.0006	Rnh1
0	1	1	2	0	1	2	2	0.0000	0.0001	0.0006	0.0006	Rbmxl1
0	0	1	0	0	0	1	0	0.0000	0.0000	0.0003	0.0000	Ppa2
0	3	1	3	0	6	6	9	0.0000	0.0008	0.0017	0.0025	Txndc5
0	1	0	1	0	4	0	1	0.0000	0.0006	0.0000	0.0003	Fubp1
0	0	1	0	0	0	1	0	0.0000	0.0000	0.0003	0.0000	Yars
0	4	1	4	0	28	4	11	0.0000	0.0039	0.0011	0.0031	Cela1
3	8	6	4	6	82	27	6	0.0046	0.0115	0.0077	0.0017	Basp1
0	1	0	0	0	2	0	0	0.0000	0.0003	0.0000	0.0000	F11

0	0	1	0	0	0	1	0	0.0000	0.0000	0.0003	0.0000	Wasl
0	1	1	3	0	1	4	6	0.0000	0.0001	0.0011	0.0017	Rpn1
1	1	1	1	7	3	1	1	0.0054	0.0004	0.0003	0.0003	Nrif2
0	1	0	1	0	3	0	3	0.0000	0.0004	0.0000	0.0008	Tardbp
0	1	0	0	0	2	0	0	0.0000	0.0003	0.0000	0.0000	Tf
0	0	0	1	0	0	0	2	0.0000	0.0000	0.0000	0.0006	Tubb6
1	3	2	1	1	8	3	2	0.0008	0.0011	0.0009	0.0006	Ptbp1
1	4	2	5	6	22	14	24	0.0046	0.0031	0.0040	0.0068	Krt5
0	1	0	0	0	4	0	0	0.0000	0.0006	0.0000	0.0000	Sf3b5
0	1	0	3	0	2	0	4	0.0000	0.0003	0.0000	0.0011	Taldo1
0	2	0	2	0	17	0	6	0.0000	0.0024	0.0000	0.0017	Hnrnpab
0	0	0	1	0	0	0	2	0.0000	0.0000	0.0000	0.0006	Nans
0	0	1	0	0	0	1	0	0.0000	0.0000	0.0003	0.0000	Psip1
0	1	1	1	0	4	3	2	0.0000	0.0006	0.0009	0.0006	Rap1b
0	1	0	0	0	1	0	0	0.0000	0.0001	0.0000	0.0000	Ggt7
0	0	0	3	0	0	0	4	0.0000	0.0000	0.0000	0.0011	Actr3
0	2	1	0	0	4	4	0	0.0000	0.0006	0.0011	0.0000	Nono
0	0	0	1	0	0	0	1	0.0000	0.0000	0.0000	0.0003	Vwa5a
0	0	1	0	0	0	2	0	0.0000	0.0000	0.0006	0.0000	Card14
0	2	1	2	0	4	2	5	0.0000	0.0006	0.0006	0.0014	Tmed9
0	0	1	1	0	0	2	2	0.0000	0.0000	0.0006	0.0006	Aco2
0	0	0	1	0	0	0	1	0.0000	0.0000	0.0000	0.0003	Dctn2
0	0	0	1	0	0	0	2	0.0000	0.0000	0.0000	0.0006	Dnajb11
0	0	0	1	0	0	0	2	0.0000	0.0000	0.0000	0.0006	St13
0	1	0	1	0	2	0	3	0.0000	0.0003	0.0000	0.0008	Etfa
0	0	1	0	0	0	2	0	0.0000	0.0000	0.0006	0.0000	Ddah2
0	1	0	0	0	1	0	0	0.0000	0.0001	0.0000	0.0000	Gpx7
0	0	1	2	0	0	1	3	0.0000	0.0000	0.0003	0.0008	Park7
0	1	1	0	0	10	3	0	0.0000	0.0014	0.0009	0.0000	Krt82
0	2	1	0	0	9	2	0	0.0000	0.0013	0.0006	0.0000	Nasp
1	0	0	0	1	0	0	0	0.0008	0.0000	0.0000	0.0000	Srrt
0	1	0	3	0	1	0	4	0.0000	0.0001	0.0000	0.0011	Rtn4
0	8	6	11	0	34	20	20	0.0000	0.0048	0.0057	0.0056	Rrbp1
0	0	0	1	0	0	0	3	0.0000	0.0000	0.0000	0.0008	Krt23
1	2	0	4	2	5	0	8	0.0015	0.0007	0.0000	0.0023	Arhgdia
0	1	1	2	0	2	4	3	0.0000	0.0003	0.0011	0.0008	2210010C04Rik
1	1	0	0	2	2	0	0	0.0015	0.0003	0.0000	0.0000	Cox6c
0	0	0	1	0	0	0	1	0.0000	0.0000	0.0000	0.0003	Cenpq
0	1	2	3	0	5	11	7	0.0000	0.0007	0.0031	0.0020	Rpl17
0	0	1	0	0	0	2	0	0.0000	0.0000	0.0006	0.0000	Glod4
0	0	1	0	0	0	2	0	0.0000	0.0000	0.0006	0.0000	Arpc5
0	5	2	5	0	61	14	11	0.0000	0.0086	0.0040	0.0031	Cela3b
0	0	0	1	0	0	0	1	0.0000	0.0000	0.0000	0.0003	Mtap
0	4	0	0	0	18	0	0	0.0000	0.0025	0.0000	0.0000	Clps
0	0	0	1	0	0	0	1	0.0000	0.0000	0.0000	0.0003	Pcyox1
0	1	0	0	0	1	0	0	0.0000	0.0001	0.0000	0.0000	Cotl1
0	0	1	0	0	0	1	0	0.0000	0.0000	0.0003	0.0000	Atp5f1
0	2	3	2	0	16	13	2	0.0000	0.0023	0.0037	0.0006	Rps21
1	5	3	7	2	41	12	17	0.0015	0.0058	0.0034	0.0048	Ywhab
2	8	2	4	21	233	9	14	0.0162	0.0328	0.0026	0.0039	Ctrb1
0	2	2	3	0	15	11	8	0.0000	0.0021	0.0031	0.0023	Rpl14
0	1	2	7	0	2	4	11	0.0000	0.0003	0.0011	0.0031	Tppp3
1	1	0	0	2	1	0	0	0.0015	0.0001	0.0000	0.0000	Filip1
0	3	1	5	0	6	11	14	0.0000	0.0008	0.0031	0.0039	Tubb2b
0	1	0	0	0	2	0	0	0.0000	0.0003	0.0000	0.0000	Rbm8a
0	0	0	1	0	0	0	2	0.0000	0.0000	0.0000	0.0006	Cygb
0	0	0	1	0	0	0	2	0.0000	0.0000	0.0000	0.0006	Hnrnpa0
0	1	0	1	0	1	0	1	0.0000	0.0001	0.0000	0.0003	Rpl11
1	2	1	2	2	5	11	4	0.0015	0.0007	0.0031	0.0011	Ssr1
1	2	0	2	1	8	0	4	0.0008	0.0011	0.0000	0.0011	Serbp1
0	0	1	1	0	0	1	1	0.0000	0.0000	0.0003	0.0003	Tpd52l2
0	0	1	0	0	0	1	0	0.0000	0.0000	0.0003	0.0000	Uqcrc1
0	0	0	1	0	0	0	1	0.0000	0.0000	0.0000	0.0003	Cmtm6
0	0	1	0	0	0	1	0	0.0000	0.0000	0.0003	0.0000	Rpl15
0	1	0	0	0	1	0	0	0.0000	0.0001	0.0000	0.0000	Shmt2
2	4	1	3	8	19	4	4	0.0062	0.0027	0.0011	0.0011	Rps19
0	0	1	0	0	0	1	0	0.0000	0.0000	0.0003	0.0000	Pdhb
0	1	1	1	0	1	3	2	0.0000	0.0001	0.0009	0.0006	Hnrnpm
0	1	1	0	0	1	1	0	0.0000	0.0001	0.0003	0.0000	Pgm1
1	1	1	1	2	15	9	5	0.0015	0.0021	0.0026	0.0014	Ptms
0	1	0	0	0	2	0	0	0.0000	0.0003	0.0000	0.0000	Oxct1

0	0	1	0	0	0	1	0	0.0000	0.0000	0.0003	0.0000	Nhp2l1
0	2	0	3	0	4	0	4	0.0000	0.0006	0.0000	0.0011	Tmed10
0	1	0	0	0	2	0	0	0.0000	0.0003	0.0000	0.0000	Cthrc1
0	1	1	0	0	1	2	0	0.0000	0.0001	0.0006	0.0000	Sarnp
0	0	1	0	0	0	2	0	0.0000	0.0000	0.0006	0.0000	Sec13
0	1	1	1	0	5	3	1	0.0000	0.0007	0.0009	0.0003	Rpl34
0	0	0	1	0	0	0	2	0.0000	0.0000	0.0000	0.0006	Fam114a1
0	1	1	1	0	2	6	2	0.0000	0.0003	0.0017	0.0006	Ube2v2
0	1	0	0	0	1	0	0	0.0000	0.0001	0.0000	0.0000	Acp1
1	2	1	0	9	8	1	0	0.0069	0.0011	0.0003	0.0000	Atp5d
0	0	0	1	0	0	0	1	0.0000	0.0000	0.0000	0.0003	Sec23b
0	1	0	0	0	1	0	0	0.0000	0.0001	0.0000	0.0000	Drap1
0	0	1	1	0	0	3	1	0.0000	0.0000	0.0009	0.0003	Mrpl2
0	1	1	1	0	3	5	1	0.0000	0.0004	0.0014	0.0003	Chmp4b
0	0	1	1	0	0	4	2	0.0000	0.0000	0.0011	0.0006	Rpl4
0	1	3	1	0	2	10	2	0.0000	0.0003	0.0028	0.0006	Eef1g
0	1	1	0	0	4	2	0	0.0000	0.0006	0.0006	0.0000	C1qtnf2
0	1	0	0	0	1	0	0	0.0000	0.0001	0.0000	0.0000	Phpt1
0	2	0	0	0	5	0	0	0.0000	0.0007	0.0000	0.0000	Cnn3
1	1	1	0	1	2	1	0	0.0008	0.0003	0.0003	0.0000	Uqcrc2
0	0	1	0	0	0	1	0	0.0000	0.0000	0.0003	0.0000	Plin3
1	0	0	1	3	0	0	2	0.0023	0.0000	0.0000	0.0006	Rpn2
2	5	3	3	5	23	6	6	0.0039	0.0032	0.0017	0.0017	Pgam1
0	0	1	0	0	0	1	0	0.0000	0.0000	0.0003	0.0000	Cmpk1
0	0	0	1	0	0	0	1	0.0000	0.0000	0.0000	0.0003	Ikbip
0	1	0	0	0	2	0	0	0.0000	0.0003	0.0000	0.0000	Abcb6
0	0	1	1	0	0	5	1	0.0000	0.0000	0.0014	0.0003	Pgd
0	0	0	1	0	0	0	2	0.0000	0.0000	0.0000	0.0006	Ssr3
0	0	0	1	0	0	0	2	0.0000	0.0000	0.0000	0.0006	Paics
0	1	3	4	0	2	13	8	0.0000	0.0003	0.0037	0.0023	Cyb5r3
0	1	1	0	0	2	1	0	0.0000	0.0003	0.0003	0.0000	Aspdh
0	0	0	3	0	0	0	6	0.0000	0.0000	0.0000	0.0017	Krt7
0	2	3	2	0	5	8	2	0.0000	0.0007	0.0023	0.0006	Etfb
0	0	1	0	0	0	8	0	0.0000	0.0000	0.0023	0.0000	Atp5h
0	1	0	0	0	2	0	0	0.0000	0.0003	0.0000	0.0000	Fgf23
1	3	1	3	4	19	2	5	0.0031	0.0027	0.0006	0.0014	Set
0	1	1	5	0	4	4	10	0.0000	0.0006	0.0011	0.0028	Ctrl
0	0	1	2	0	0	1	5	0.0000	0.0000	0.0003	0.0014	Gsdma
0	1	1	0	0	2	4	0	0.0000	0.0003	0.0011	0.0000	Anp32b
0	0	0	1	0	0	0	1	0.0000	0.0000	0.0000	0.0003	Tmod3
0	0	0	1	0	0	0	1	0.0000	0.0000	0.0000	0.0003	Dync1h1
1	0	0	0	1	0	0	0	0.0008	0.0000	0.0000	0.0000	Isyna1
0	2	1	1	0	3	2	1	0.0000	0.0004	0.0006	0.0003	Akr1a1
0	1	0	0	0	1	0	0	0.0000	0.0001	0.0000	0.0000	Ralb
0	1	0	0	0	1	0	0	0.0000	0.0001	0.0000	0.0000	Caln1
0	1	1	1	0	9	3	1	0.0000	0.0013	0.0009	0.0003	Sh3bgrl
0	1	1	1	0	2	6	1	0.0000	0.0003	0.0017	0.0003	Refbp2
0	1	0	0	0	1	0	0	0.0000	0.0001	0.0000	0.0000	Myg1
0	1	0	0	0	2	0	0	0.0000	0.0003	0.0000	0.0000	Serpini2
0	1	0	0	0	3	0	0	0.0000	0.0004	0.0000	0.0000	Aldh9a1
0	0	1	0	0	0	1	0	0.0000	0.0000	0.0003	0.0000	Klra17
0	0	1	0	0	0	1	0	0.0000	0.0000	0.0003	0.0000	Hdgfrp3
0	1	2	1	0	1	7	2	0.0000	0.0001	0.0020	0.0006	Rhoa
0	0	1	1	0	0	6	3	0.0000	0.0000	0.0017	0.0008	Psma6
0	0	1	0	0	0	1	0	0.0000	0.0000	0.0003	0.0000	Kif21b
0	0	1	0	0	0	1	0	0.0000	0.0000	0.0003	0.0000	Plec
0	1	0	1	0	3	0	2	0.0000	0.0004	0.0000	0.0006	Cnpy2
0	0	0	1	0	0	0	1	0.0000	0.0000	0.0000	0.0003	Clic4
0	0	0	3	0	0	0	5	0.0000	0.0000	0.0000	0.0014	Add3
0	0	2	2	0	0	5	4	0.0000	0.0000	0.0014	0.0011	Add1
0	1	0	0	0	1	0	0	0.0000	0.0001	0.0000	0.0000	Eif3i
0	1	0	0	0	1	0	0	0.0000	0.0001	0.0000	0.0000	Galk1
0	0	1	0	0	0	1	0	0.0000	0.0000	0.0003	0.0000	Esd
0	0	0	4	0	0	0	7	0.0000	0.0000	0.0000	0.0020	Dstn
0	1	0	0	0	1	0	0	0.0000	0.0001	0.0000	0.0000	Sec11a
1	2	1	1	1	7	2	2	0.0008	0.0010	0.0006	0.0006	Tmed2
0	0	0	2	0	0	0	3	0.0000	0.0000	0.0000	0.0008	Try4
0	1	1	1	0	5	3	1	0.0000	0.0007	0.0009	0.0003	Ikbke
0	0	0	1	0	0	0	1	0.0000	0.0000	0.0000	0.0003	Ak1
0	2	0	0	0	4	0	0	0.0000	0.0006	0.0000	0.0000	Psma4
0	1	1	1	0	4	3	2	0.0000	0.0006	0.0009	0.0006	Psmb2

0	2	1	0	0	5	1	0	0.0000	0.0007	0.0003	0.0000	Psma1
0	0	0	1	0	0	0	1	0.0000	0.0000	0.0000	0.0003	Arntl
0	2	0	0	0	7	0	0	0.0000	0.0010	0.0000	0.0000	Ak2
0	8	9	10	0	52	36	14	0.0000	0.0073	0.0102	0.0039	Akap12
0	1	1	0	0	5	4	0	0.0000	0.0007	0.0011	0.0000	Skp1
0	0	0	1	0	0	0	2	0.0000	0.0000	0.0000	0.0006	Eif4h
0	0	0	1	0	0	0	1	0.0000	0.0000	0.0000	0.0003	Coro1c
0	1	0	0	0	3	0	0	0.0000	0.0004	0.0000	0.0000	Ctsz
0	0	1	1	0	0	5	1	0.0000	0.0000	0.0014	0.0003	Snx1
0	2	2	5	0	10	7	15	0.0000	0.0014	0.0020	0.0042	Tagln2
0	0	0	2	0	0	0	3	0.0000	0.0000	0.0000	0.0008	Ehd1
0	0	0	2	0	0	0	3	0.0000	0.0000	0.0000	0.0008	Gbp2
0	2	0	1	0	2	0	1	0.0000	0.0003	0.0000	0.0003	Aifm1
1	1	0	0	1	2	0	0	0.0008	0.0003	0.0000	0.0000	Ddx39b
0	2	1	1	0	3	4	1	0.0000	0.0004	0.0011	0.0003	Clic1
0	1	1	0	0	3	3	0	0.0000	0.0004	0.0009	0.0000	Vars
0	1	0	1	0	2	0	5	0.0000	0.0003	0.0000	0.0014	Prss1
0	1	0	0	0	2	0	0	0.0000	0.0003	0.0000	0.0000	Thbs4
0	1	0	0	0	2	0	0	0.0000	0.0003	0.0000	0.0000	Strap
0	4	0	2	0	6	0	2	0.0000	0.0008	0.0000	0.0006	Hnrnpc
0	0	1	0	0	0	2	0	0.0000	0.0000	0.0006	0.0000	Pmm2
0	2	2	2	0	6	9	6	0.0000	0.0008	0.0026	0.0017	Psma7
0	0	0	1	0	0	0	2	0.0000	0.0000	0.0000	0.0006	Psma5
0	0	1	0	0	0	1	0	0.0000	0.0000	0.0003	0.0000	Dnpep
0	1	0	1	0	4	0	1	0.0000	0.0006	0.0000	0.0003	Hnrnpf

protein	annotation	FDR e15.5	e17.5	p2	p14	Unique Petides e15.5	e17.5	p2
E9Q447	Uncharacterized protein OS=Mus musculus GN=Spna2 PE=4 SV=1	1.00	0.00	0.00	0.00	0	3	8
O08547	Vesicle-trafficking protein SEC22b OS=Mus musculus GN=Sec22b PE=1 SV=3	1.00	1.00	1.00	0.02	0	0	0
O35074	Prostacyclin synthase OS=Mus musculus GN=Ptgis PE=2 SV=1	1.00	1.00	1.00	0.00	0	0	0
O35129	Prohibitin-2 OS=Mus musculus GN=Phb2 PE=1 SV=1	1.00	0.02	0.02	1.00	0	1	1
O35682	Myeloid-associated differentiation marker OS=Mus musculus GN=Myadm PE=2 SV=2	1.00	1.00	1.00	0.02	0	0	0
O54734	Dolichyl-diphosphooligosaccharide--protein glycosyltransferase 48 kDa subunit OS=Mus musculu	1.00	0.02	1.00	0.00	0	1	0
O55022	Membrane-associated progesterone receptor component 1 OS=Mus musculus GN=Pgrmc1 PE=	1.00	0.02	1.00	1.00	0	1	0
O55026	Ectonucleoside triphosphate diphosphohydrolase 2 OS=Mus musculus GN=Entpd2 PE=1 SV=2	1.00	1.00	1.00	0.00	0	0	0
O55029	Coatomer subunit beta' OS=Mus musculus GN=Copb2 PE=2 SV=2	1.00	1.00	1.00	0.02	0	0	0
O88962	7-alpha-hydroxycholest-4-en-3-one 12-alpha-hydroxylase OS=Mus musculus GN=Cyp8b1 PE=2	1.00	1.00	1.00	0.07	0	0	0
P05064	Fructose-bisphosphate aldolase A OS=Mus musculus GN=Aldoa PE=1 SV=2	0.02	0.00	0.00	0.00	1	9	4
P11688	Integrin alpha-5 OS=Mus musculus GN=Itga5 PE=1 SV=3	1.00	0.04	0.02	1.00	0	1	1
P14431	H-2 class I histocompatibility antigen, Q9 alpha chain (Fragment) OS=Mus musculus GN=H2-Q9	1.00	0.04	1.00	1.00	0	1	0
P14733	Lamin-B1 OS=Mus musculus GN=Lmnb1 PE=1 SV=3	0.00	0.00	0.00	0.00	2	5	5
P21981	Protein-glutamine gamma-glutamyltransferase 2 OS=Mus musculus GN=Tgm2 PE=1 SV=4	1.00	1.00	1.00	0.00	0	0	0
P26645	Myristoylated alanine-rich C-kinase substrate OS=Mus musculus GN=Marcks PE=1 SV=2	0.00	0.00	0.00	0.00	5	6	4
P35564	Calnexin OS=Mus musculus GN=Canx PE=1 SV=1	1.00	0.00	1.00	0.00	0	2	0
P45878	Peptidyl-prolyl cis-trans isomerase FKBP2 OS=Mus musculus GN=Fkbp2 PE=1 SV=1	1.00	0.02	1.00	1.00	0	1	0
P47740	Fatty aldehyde dehydrogenase OS=Mus musculus GN=Aldh3a2 PE=2 SV=2	1.00	1.00	1.00	0.02	0	0	0
P47753	F-actin-capping protein subunit alpha-1 OS=Mus musculus GN=Capza1 PE=1 SV=4	1.00	0.02	0.02	0.02	0	1	1
P47757	F-actin-capping protein subunit beta OS=Mus musculus GN=Capzb PE=1 SV=3	1.00	0.03	0.03	0.02	0	1	1
P48771	Cytochrome c oxidase subunit 7A2, mitochondrial OS=Mus musculus GN=Cox7a2 PE=1 SV=2	1.00	0.02	1.00	1.00	0	1	0
P56395	Cytochrome b5 OS=Mus musculus GN=Cyb5a PE=1 SV=2	1.00	1.00	1.00	0.02	0	0	0
P56501	Mitochondrial uncoupling protein 3 OS=Mus musculus GN=Ucp3 PE=2 SV=1	0.05	0.03	1.00	0.08	1	1	0
P61168	D(2) dopamine receptor OS=Mus musculus GN=Drd2 PE=2 SV=2	0.04	0.03	1.00	1.00	1	1	0
P61804	Dolichyl-diphosphooligosaccharide--protein glycosyltransferase subunit DAD1 OS=Mus musculus	1.00	0.03	1.00	0.00	0	1	0
P63005	Platelet-activating factor acetylhydrolase IB subunit alpha OS=Mus musculus GN=Pafah1b1 PE=	1.00	1.00	0.02	1.00	0	0	1
P63242	Eukaryotic translation initiation factor 5A-1 OS=Mus musculus GN=Eif5a PE=1 SV=2	0.02	0.00	0.04	0.00	1	2	1
P67778	Prohibitin OS=Mus musculus GN=Phb PE=1 SV=1	1.00	1.00	0.02	1.00	0	0	1
P70441	Na(+)/H(+) exchange regulatory cofactor NHE-RF1 OS=Mus musculus GN=Slc9a3r1 PE=1 SV=3	1.00	1.00	0.02	0.00	0	0	1
P97449	Aminopeptidase N OS=Mus musculus GN=Anpep PE=1 SV=4	1.00	1.00	0.03	0.00	0	0	1
P97450	ATP synthase-coupling factor 6, mitochondrial OS=Mus musculus GN=Atp5j PE=1 SV=1	1.00	0.04	1.00	1.00	0	1	0
Q02257	Junction plakoglobin OS=Mus musculus GN=Jup PE=1 SV=3	1.00	1.00	1.00	0.00	0	0	0
Q05144	Ras-related C3 botulinum toxin substrate 2 OS=Mus musculus GN=Rac2 PE=2 SV=1	1.00	1.00	0.02	0.02	0	0	1
Q06185	ATP synthase subunit e, mitochondrial OS=Mus musculus GN=Atp5i PE=1 SV=2	1.00	0.02	0.02	1.00	0	1	1
Q07113	Cation-independent mannose-6-phosphate receptor OS=Mus musculus GN=Igf2r PE=1 SV=1	0.02	0.00	0.00	1.00	1	8	4
Q09163	Protein delta homolog 1 OS=Mus musculus GN=Dlk1 PE=1 SV=1	1.00	0.04	1.00	1.00	0	1	0
Q3U7R1	Extended synaptotagmin-1 OS=Mus musculus GN=Esyt1 PE=2 SV=2	1.00	1.00	1.00	0.03	0	0	0
Q5F4T0	Transient receptor potential cation channel, subfamily M, member 3 OS=Mus musculus GN=Trpm	1.00	0.02	1.00	1.00	0	1	0
Q5XJY5	Coatomer subunit delta OS=Mus musculus GN=Arcn1 PE=2 SV=2	1.00	1.00	1.00	0.02	0	0	0
Q60870	Receptor expression-enhancing protein 5 OS=Mus musculus GN=Reep5 PE=1 SV=1	1.00	1.00	0.02	1.00	0	0	1
Q60931	Voltage-dependent anion-selective channel protein 3 OS=Mus musculus GN=Vdac3 PE=1 SV=1	1.00	1.00	1.00	0.07	0	0	0
Q60932	Voltage-dependent anion-selective channel protein 1 OS=Mus musculus GN=Vdac1 PE=1 SV=3	1.00	0.00	0.00	0.00	0	2	3
Q60997	Deleted in malignant brain tumors 1 protein OS=Mus musculus GN=Dmbt1 PE=1 SV=2	1.00	1.00	0.00	1.00	0	0	2
Q61029	Lamina-associated polypeptide 2, isoforms beta/delta/epsilon/gamma OS=Mus musculus GN=Tm	1.00	0.02	0.00	0.00	0	1	2
Q61409	cGMP-inhibited 3',5'-cyclic phosphodiesterase B OS=Mus musculus GN=Pde3b PE=1 SV=2	1.00	0.02	1.00	1.00	0	1	0
Q61598	Rab GDP dissociation inhibitor beta OS=Mus musculus GN=Gdi2 PE=1 SV=1	1.00	1.00	1.00	0.02	0	0	0
Q62261	Spectrin beta chain, brain 1 OS=Mus musculus GN=Sptbn1 PE=1 SV=2	1.00	0.05	0.00	0.00	0	1	8
Q63836	Selenium-binding protein 2 OS=Mus musculus GN=Selenbp2 PE=1 SV=2	1.00	0.02	0.02	0.02	0	1	1
Q640L3	Cell cycle progression protein 1 OS=Mus musculus GN=Ccpg1 PE=1 SV=2	1.00	0.04	1.00	1.00	0	1	0
Q64314	Hematopoietic progenitor cell antigen CD34 OS=Mus musculus GN=Cd34 PE=1 SV=1	1.00	0.07	1.00	1.00	0	1	0
Q68FD5	Clathrin heavy chain 1 OS=Mus musculus GN=Cltc PE=1 SV=3	1.00	1.00	1.00	0.00	0	0	0
Q80TN7	Neuron navigator 3 OS=Mus musculus GN=Nav3 PE=1 SV=2	0.04	1.00	1.00	1.00	1	0	0
Q80WJ7	Protein LYRIC OS=Mus musculus GN=Mtdh PE=1 SV=1	1.00	1.00	0.05	1.00	0	0	1
Q8BH64	EH domain-containing protein 2 OS=Mus musculus GN=Ehd2 PE=1 SV=1	1.00	1.00	1.00	0.00	0	0	0
Q8BSY0	Aspartyl/asparaginyl beta-hydroxylase OS=Mus musculus GN=Asph PE=2 SV=1	1.00	1.00	1.00	0.02	0	0	0
Q8BUE4	Apoptosis-inducing factor 2 OS=Mus musculus GN=Aifm2 PE=2 SV=1	1.00	1.00	0.05	0.02	0	0	1
Q8CIE6	Coatomer subunit alpha OS=Mus musculus GN=Copa PE=1 SV=2	1.00	1.00	1.00	0.02	0	0	0
Q8K2B3	Succinate dehydrogenase [ubiquinone] flavoprotein subunit, mitochondrial OS=Mus musculus GN	0.03	0.02	0.02	0.03	1	1	1
Q8R207	Small subunit of serine palmitoyltransferase A OS=Mus musculus GN=Ssspta PE=2 SV=2	1.00	0.02	0.02	0.02	0	1	1
Q91YQ5	Dolichyl-diphosphooligosaccharide--protein glycosyltransferase subunit 1 OS=Mus musculus GN=	1.00	0.04	0.03	0.00	0	1	1
Q99JP7	Gamma-glutamyltransferase 7 OS=Mus musculus GN=Ggt7 PE=1 SV=2	1.00	0.07	1.00	1.00	0	1	0
Q99KF1	Transmembrane emp24 domain-containing protein 9 OS=Mus musculus GN=Tmed9 PE=2 SV=2	1.00	0.00	0.02	0.00	0	2	1
Q99KJ8	Dynactin subunit 2 OS=Mus musculus GN=Dctn2 PE=1 SV=3	1.00	1.00	1.00	0.02	0	0	0
Q99PL5	Ribosome-binding protein 1 OS=Mus musculus GN=Rrbp1 PE=2 SV=2	1.00	0.00	0.00	0.00	0	8	6
Q9CPQ1	Cytochrome c oxidase subunit 6C OS=Mus musculus GN=Cox6c PE=1 SV=3	0.04	0.02	1.00	1.00	1	1	0
Q9CQQ7	ATP synthase subunit b, mitochondrial OS=Mus musculus GN=Atp5f1 PE=1 SV=1	1.00	1.00	0.05	1.00	0	0	1
Q9CY50	Translocon-associated protein subunit alpha OS=Mus musculus GN=Ssr1 PE=1 SV=1	0.06	0.00	0.02	0.00	1	2	1
Q9CZ13	Cytochrome b-c1 complex subunit 1, mitochondrial OS=Mus musculus GN=Uqcrc1 PE=1 SV=2	1.00	1.00	0.02	1.00	0	0	1
Q9CZ69	CKLF-like MARVEL transmembrane domain-containing protein 6 OS=Mus musculus GN=Cmtm6	1.00	1.00	1.00	0.04	0	0	0
Q9D1M0	Protein SEC13 homolog OS=Mus musculus GN=Sec13 PE=2 SV=3	1.00	1.00	0.02	1.00	0	0	1
Q9D3D9	ATP synthase subunit delta, mitochondrial OS=Mus musculus GN=Atp5d PE=1 SV=1	0.02	0.00	0.03	1.00	1	2	1
Q9D662	Protein transport protein Sec23B OS=Mus musculus GN=Sec23b PE=2 SV=1	1.00	1.00	1.00	0.02	0	0	0

Q9D8B3	Charged multivesicular body protein 4b OS=Mus musculus GN=Chmp4b PE=2 SV=2	1.00	0.02	0.02	0.02	0	1	1
Q9DB77	Cytochrome b-c1 complex subunit 2, mitochondrial OS=Mus musculus GN=Uqcrc2 PE=1 SV=1	0.04	0.02	0.02	1.00	1	1	1
Q9DBG5	Perilipin-3 OS=Mus musculus GN=Plin3 PE=1 SV=1	1.00	1.00	0.03	1.00	0	0	1
Q9DBG6	Dolichyl-diphosphooligosaccharide--protein glycosyltransferase subunit 2 OS=Mus musculus GN=	0.03	1.00	1.00	0.02	1	0	0
Q9DBZ1	Inhibitor of nuclear factor kappa-B kinase-interacting protein OS=Mus musculus GN=Ikbip PE=1 S	1.00	1.00	1.00	0.07	0	0	0
Q9DCF9	Translocon-associated protein subunit gamma OS=Mus musculus GN=Ssr3 PE=1 SV=1	1.00	1.00	1.00	0.02	0	0	0
Q9DCN2	NADH-cytochrome b5 reductase 3 OS=Mus musculus GN=Cyb5r3 PE=1 SV=3	1.00	0.04	0.00	0.00	0	1	3
Q9DCX2	ATP synthase subunit d, mitochondrial OS=Mus musculus GN=Atp5h PE=1 SV=3	1.00	1.00	0.02	1.00	0	0	1
Q9QYB5	Gamma-adducin OS=Mus musculus GN=Add3 PE=1 SV=2	1.00	1.00	1.00	0.00	0	0	0
Q9QYC0	Alpha-adducin OS=Mus musculus GN=Add1 PE=1 SV=2	1.00	1.00	0.00	0.00	0	0	2
Q9R0P6	Signal peptidase complex catalytic subunit SEC11A OS=Mus musculus GN=Sec11a PE=2 SV=1	1.00	0.03	1.00	1.00	0	1	0
Q9R0Q3	Transmembrane emp24 domain-containing protein 2 OS=Mus musculus GN=Tmed2 PE=1 SV=1	0.02	0.00	0.02	0.03	1	2	1
Q9WV80	Sorting nexin-1 OS=Mus musculus GN=Snx1 PE=1 SV=1	1.00	1.00	0.02	0.02	0	0	1
Q9Z0X1	Apoptosis-inducing factor 1, mitochondrial OS=Mus musculus GN=Aifm1 PE=1 SV=1	1.00	0.00	1.00	0.06	0	2	0

	Spectral Counts				Normalized Spectral Counts				
p14	e15.5	e17.5	p2	p14	e15.5	e17.5	p2	p14	gene
35	0	12	22	74	0.0000	0.0017	0.0062	0.0208	Spna2
1	0	0	0	1	0.0000	0.0000	0.0000	0.0003	Sec22b
2	0	0	0	7	0.0000	0.0000	0.0000	0.0020	Ptgis
0	0	7	2	0	0.0000	0.0010	0.0006	0.0000	Phb2
1	0	0	0	1	0.0000	0.0000	0.0000	0.0003	Myadm
2	0	2	0	4	0.0000	0.0003	0.0000	0.0011	Ddost
0	0	3	0	0	0.0000	0.0004	0.0000	0.0000	Pgrmc1
2	0	0	0	2	0.0000	0.0000	0.0000	0.0006	Entpd2
1	0	0	0	2	0.0000	0.0000	0.0000	0.0006	Copb2
1	0	0	0	1	0.0000	0.0000	0.0000	0.0003	Cyp8b1
2	2	35	15	4	0.0015	0.0049	0.0043	0.0011	Aldoa
0	0	1	2	0	0.0000	0.0001	0.0006	0.0000	Itga5
0	0	1	0	0	0.0000	0.0001	0.0000	0.0000	H2-Q9
8	3	21	15	16	0.0023	0.0030	0.0043	0.0045	Lmnb1
6	0	0	0	12	0.0000	0.0000	0.0000	0.0034	Tgm2
3	33	68	24	9	0.0255	0.0096	0.0068	0.0025	Marcks
8	0	8	0	14	0.0000	0.0011	0.0000	0.0039	Canx
0	0	4	0	0	0.0000	0.0006	0.0000	0.0000	Fkbp2
1	0	0	0	1	0.0000	0.0000	0.0000	0.0003	Aldh3a2
1	0	1	2	2	0.0000	0.0001	0.0006	0.0006	Capza1
1	0	1	1	2	0.0000	0.0001	0.0003	0.0006	Capzb
0	0	2	0	0	0.0000	0.0003	0.0000	0.0000	Cox7a2
1	0	0	0	1	0.0000	0.0000	0.0000	0.0003	Cyb5a
1	2	1	0	1	0.0015	0.0001	0.0000	0.0003	Ucp3
0	3	5	0	0	0.0023	0.0007	0.0000	0.0000	Drd2
2	0	1	0	4	0.0000	0.0001	0.0000	0.0011	Dad1
0	0	0	2	0	0.0000	0.0000	0.0006	0.0000	Pafah1b1
4	8	7	2	8	0.0062	0.0010	0.0006	0.0023	Eif5a
0	0	0	1	0	0.0000	0.0000	0.0003	0.0000	Phb
2	0	0	4	5	0.0000	0.0000	0.0011	0.0014	Slc9a3r1
2	0	0	2	3	0.0000	0.0000	0.0006	0.0008	Anpep
0	0	2	0	0	0.0000	0.0003	0.0000	0.0000	Atp5j
8	0	0	0	18	0.0000	0.0000	0.0000	0.0051	Jup
1	0	0	4	2	0.0000	0.0000	0.0011	0.0006	Rac2
0	0	2	5	0	0.0000	0.0003	0.0014	0.0000	Atp5i
0	2	16	12	0	0.0015	0.0023	0.0034	0.0000	Igf2r
0	0	2	0	0	0.0000	0.0003	0.0000	0.0000	Dlk1
1	0	0	0	2	0.0000	0.0000	0.0000	0.0006	Esyt1
0	0	1	0	0	0.0000	0.0001	0.0000	0.0000	Trpm3
1	0	0	0	1	0.0000	0.0000	0.0000	0.0003	Arcn1
0	0	0	1	0	0.0000	0.0000	0.0003	0.0000	Reep5
1	0	0	0	1	0.0000	0.0000	0.0000	0.0003	Vdac3
2	0	13	5	4	0.0000	0.0018	0.0014	0.0011	Vdac1
0	0	0	3	0	0.0000	0.0000	0.0009	0.0000	Dmbt1
2	0	1	5	3	0.0000	0.0001	0.0014	0.0008	Tmpo
0	0	2	0	0	0.0000	0.0003	0.0000	0.0000	Pde3b
1	0	0	0	1	0.0000	0.0000	0.0000	0.0003	Gdi2
27	0	1	23	57	0.0000	0.0001	0.0065	0.0161	Sptbn1
1	0	14	6	2	0.0000	0.0020	0.0017	0.0006	Selenbp2
0	0	1	0	0	0.0000	0.0001	0.0000	0.0000	Ccpg1
0	0	2	0	0	0.0000	0.0003	0.0000	0.0000	Cd34
2	0	0	0	2	0.0000	0.0000	0.0000	0.0006	Cltc
0	3	0	0	0	0.0023	0.0000	0.0000	0.0000	Nav3
0	0	0	2	0	0.0000	0.0000	0.0006	0.0000	Mtdh
3	0	0	0	7	0.0000	0.0000	0.0000	0.0020	Ehd2
1	0	0	0	2	0.0000	0.0000	0.0000	0.0006	Asph
1	0	0	2	2	0.0000	0.0000	0.0006	0.0006	Aifm2
1	0	0	0	1	0.0000	0.0000	0.0000	0.0003	Copa
1	3	3	6	2	0.0023	0.0004	0.0017	0.0006	Sdha
1	0	3	6	6	0.0000	0.0004	0.0017	0.0017	Ssspta
3	0	1	4	6	0.0000	0.0001	0.0011	0.0017	Rpn1
0	0	1	0	0	0.0000	0.0001	0.0000	0.0000	Ggt7
2	0	4	2	5	0.0000	0.0006	0.0006	0.0014	Tmed9
1	0	0	0	1	0.0000	0.0000	0.0000	0.0003	Dctn2
11	0	34	20	20	0.0000	0.0048	0.0057	0.0056	Rrbp1
0	2	2	0	0	0.0015	0.0003	0.0000	0.0000	Cox6c
0	0	0	1	0	0.0000	0.0000	0.0003	0.0000	Atp5f1
2	2	5	11	4	0.0015	0.0007	0.0031	0.0011	Ssr1
0	0	0	1	0	0.0000	0.0000	0.0003	0.0000	Uqcrc1
1	0	0	0	1	0.0000	0.0000	0.0000	0.0003	Cmtm6
0	0	0	2	0	0.0000	0.0000	0.0006	0.0000	Sec13
0	9	8	1	0	0.0069	0.0011	0.0003	0.0000	Atp5d
1	0	0	0	1	0.0000	0.0000	0.0000	0.0003	Sec23b

1	0	3	5	1	0.0000	0.0004	0.0014	0.0003	Chmp4b
0	1	2	1	0	0.0008	0.0003	0.0003	0.0000	Uqcrc2
0	0	0	1	0	0.0000	0.0000	0.0003	0.0000	Plin3
1	3	0	0	2	0.0023	0.0000	0.0000	0.0006	Rpn2
1	0	0	0	1	0.0000	0.0000	0.0000	0.0003	Ikbip
1	0	0	0	2	0.0000	0.0000	0.0000	0.0006	Ssr3
4	0	2	13	8	0.0000	0.0003	0.0037	0.0023	Cyb5r3
0	0	0	8	0	0.0000	0.0000	0.0023	0.0000	Atp5h
3	0	0	0	5	0.0000	0.0000	0.0000	0.0014	Add3
2	0	0	5	4	0.0000	0.0000	0.0014	0.0011	Add1
0	0	1	0	0	0.0000	0.0001	0.0000	0.0000	Sec11a
1	1	7	2	2	0.0008	0.0010	0.0006	0.0006	Tmed2
1	0	0	5	1	0.0000	0.0000	0.0014	0.0003	Snx1
1	0	2	0	1	0.0000	0.0003	0.0000	0.0003	Aifm1

		FDR				Unique Petides				Spectral
protein	annotation	e15.5	e17.5	p2	p14	e15.5	e17.5	p2	p14	e15.5
E9Q557	Desmoplakin OS=Mus musculus GN=Dsp PE=3 SV=1	1.00	0.00	0.00	0.00	0	2	3	19	0
P09055	Integrin beta-1 OS=Mus musculus GN=Itgb1 PE=1 SV=1	1.00	0.03	0.08	0.04	0	1	1	1	0
P11688	Integrin alpha-5 OS=Mus musculus GN=Itga5 PE=1 SV=3	1.00	0.04	0.02	1.00	0	1	1	0	0
P13020	Gelsolin OS=Mus musculus GN=Gsn PE=1 SV=3	0.05	0.00	0.00	0.00	1	3	4	3	2
P16045	Galectin-1 OS=Mus musculus GN=Lgals1 PE=1 SV=3	0.02	0.00	0.00	0.00	1	6	4	7	7
P19157	Glutathione S-transferase P 1 OS=Mus musculus GN=Gstp1 PE=1 SV=2	1.00	0.02	1.00	0.00	0	1	0	3	0
P21981	Protein-glutamine gamma-glutamyltransferase 2 OS=Mus musculus GN=Tgm2 PE=1 SV=4	1.00	1.00	1.00	0.00	0	0	0	6	0
P26039	Talin-1 OS=Mus musculus GN=Tln1 PE=1 SV=2	1.00	0.02	0.02	0.00	0	1	1	5	0
P26040	Ezrin OS=Mus musculus GN=Ezr PE=1 SV=3	1.00	0.02	0.02	0.00	0	1	1	2	0
P26041	Moesin OS=Mus musculus GN=Msn PE=1 SV=3	0.03	0.00	0.00	0.00	1	5	3	4	3
P28843	Dipeptidyl peptidase 4 OS=Mus musculus GN=Dpp4 PE=1 SV=3	1.00	1.00	1.00	0.02	0	0	0	1	0
P52793	Ephrin-A1 OS=Mus musculus GN=Efna1 PE=2 SV=1	1.00	0.07	1.00	1.00	0	1	0	0	0
P56480	ATP synthase subunit beta, mitochondrial OS=Mus musculus GN=Atp5b PE=1 SV=2	0.00	0.00	0.00	0.00	4	6	3	9	21
P60766	Cell division control protein 42 homolog OS=Mus musculus GN=Cdc42 PE=1 SV=2	1.00	0.00	0.02	1.00	0	2	1	0	0
P62141	Serine/threonine-protein phosphatase PP1-beta catalytic subunit OS=Mus musculus GN=Ppp1cb PE	1.00	0.02	1.00	1.00	0	1	0	0	0
P97300	Neuroplastin OS=Mus musculus GN=Nptn PE=1 SV=3	1.00	1.00	0.07	0.08	0	0	1	1	0
P97350	Plakophilin-1 OS=Mus musculus GN=Pkp1 PE=1 SV=1	1.00	1.00	1.00	0.02	0	0	0	1	0
Q02257	Junction plakoglobin OS=Mus musculus GN=Jup PE=1 SV=3	1.00	1.00	1.00	0.00	0	0	0	8	0
Q04857	Collagen alpha-1(VI) chain OS=Mus musculus GN=Col6a1 PE=2 SV=1	1.00	1.00	0.02	0.04	0	0	1	1	0
Q64314	Hematopoietic progenitor cell antigen CD34 OS=Mus musculus GN=Cd34 PE=1 SV=1	1.00	0.07	1.00	1.00	0	1	0	0	0
Q64727	Vinculin OS=Mus musculus GN=Vcl PE=1 SV=4	1.00	0.02	0.00	0.00	0	1	4	8	0
Q8VDD5	Myosin-9 OS=Mus musculus GN=Myh9 PE=1 SV=4	1.00	1.00	0.00	0.00	0	0	3	15	0
Q9QUI0	Transforming protein RhoA OS=Mus musculus GN=Rhoa PE=1 SV=1	1.00	0.04	0.00	0.02	0	1	2	1	0
Q9Z1T2	Thrombospondin-4 OS=Mus musculus GN=Thbs4 PE=2 SV=1	1.00	0.05	1.00	1.00	0	1	0	0	0

l Counts			Normalized Spectral Counts				
e17.5	p2	p14	e15.5	e17.5	p2	p14	gene
3	5	48	0.0000	0.0004	0.0014	0.0135	Dsp
2	1	1	0.0000	0.0003	0.0003	0.0003	Itgb1
1	2	0	0.0000	0.0001	0.0006	0.0000	Itga5
20	19	5	0.0015	0.0028	0.0054	0.0014	Gsn
37	23	40	0.0054	0.0052	0.0065	0.0113	Lgals1
1	0	7	0.0000	0.0001	0.0000	0.0020	Gstp1
0	0	12	0.0000	0.0000	0.0000	0.0034	Tgm2
2	1	9	0.0000	0.0003	0.0003	0.0025	Tln1
12	4	2	0.0000	0.0017	0.0011	0.0006	Ezr
18	11	7	0.0023	0.0025	0.0031	0.0020	Msn
0	0	2	0.0000	0.0000	0.0000	0.0006	Dpp4
1	0	0	0.0000	0.0001	0.0000	0.0000	Efna1
22	9	17	0.0162	0.0031	0.0026	0.0048	Atp5b
3	3	0	0.0000	0.0004	0.0009	0.0000	Cdc42
3	0	0	0.0000	0.0004	0.0000	0.0000	Ppp1cb
0	1	1	0.0000	0.0000	0.0003	0.0003	Nptn
0	0	2	0.0000	0.0000	0.0000	0.0006	Pkp1
0	0	18	0.0000	0.0000	0.0000	0.0051	Jup
0	2	1	0.0000	0.0000	0.0006	0.0003	Col6a1
2	0	0	0.0000	0.0003	0.0000	0.0000	Cd34
4	7	14	0.0000	0.0006	0.0020	0.0039	Vcl
0	7	26	0.0000	0.0000	0.0020	0.0073	Myh9
1	7	2	0.0000	0.0001	0.0020	0.0006	Rhoa
2	0	0	0.0000	0.0003	0.0000	0.0000	Thbs4

protein	annotation	FDR e15.5	e17.5	p2	p14	Unique Petides e15.5	e17.5
O08553	Dihydropyrimidinase-related protein 2 OS=Mus musculus GN=Dpysl2 PE=1 SV=2	1.00	0.02	0.02	0.00	0	1
O35639	Annexin A3 OS=Mus musculus GN=Anxa3 PE=1 SV=4	1.00	1.00	1.00	0.00	0	0
O35855	Branched-chain-amino-acid aminotransferase, mitochondrial OS=Mus musculus GN=Bcat2 PE=2 SV=2	1.00	0.03	1.00	1.00	0	1
P07356	Annexin A2 OS=Mus musculus GN=Anxa2 PE=1 SV=2	0.00	0.00	0.00	0.00	2	6
P10107	Annexin A1 OS=Mus musculus GN=Anxa1 PE=1 SV=2	1.00	1.00	0.00	0.00	0	0
P26443	Glutamate dehydrogenase 1, mitochondrial OS=Mus musculus GN=Glud1 PE=1 SV=1	1.00	1.00	0.02	1.00	0	0
P34884	Macrophage migration inhibitory factor OS=Mus musculus GN=Mif PE=1 SV=2	0.04	0.02	1.00	1.00	1	1
P49817	Caveolin-1 OS=Mus musculus GN=Cav1 PE=1 SV=1	1.00	1.00	0.05	0.00	0	0
P52927	High mobility group protein HMGI-C OS=Mus musculus GN=Hmga2 PE=1 SV=1	0.02	0.05	1.00	1.00	1	1
P57759	Endoplasmic reticulum resident protein 29 OS=Mus musculus GN=Erp29 PE=1 SV=2	1.00	0.05	1.00	1.00	0	1
P61168	D(2) dopamine receptor OS=Mus musculus GN=Drd2 PE=2 SV=2	0.04	0.03	1.00	1.00	1	1
P63101	14-3-3 protein zeta/delta OS=Mus musculus GN=Ywhaz PE=1 SV=1	0.00	0.00	0.00	0.00	5	11
P70441	Na(+)/H(+) exchange regulatory cofactor NHE-RF1 OS=Mus musculus GN=Slc9a3r1 PE=1 SV=3	1.00	1.00	0.02	0.00	0	0
P97352	Protein S100-A13 OS=Mus musculus GN=S100a13 PE=1 SV=1	1.00	1.00	0.02	0.02	0	0
P97449	Aminopeptidase N OS=Mus musculus GN=Anpep PE=1 SV=4	1.00	1.00	0.03	0.00	0	0
Q00519	Xanthine dehydrogenase/oxidase OS=Mus musculus GN=Xdh PE=1 SV=5	1.00	1.00	1.00	0.02	0	0
Q3TJ91	Lethal(2) giant larvae protein homolog 2 OS=Mus musculus GN=Llgl2 PE=2 SV=2	1.00	0.04	1.00	1.00	0	1
Q61409	cGMP-inhibited 3',5'-cyclic phosphodiesterase B OS=Mus musculus GN=Pde3b PE=1 SV=2	1.00	0.02	1.00	1.00	0	1
Q61425	Hydroxyacyl-coenzyme A dehydrogenase, mitochondrial OS=Mus musculus GN=Hadh PE=1 SV=2	1.00	0.00	1.00	1.00	0	2
Q61879	Myosin-10 OS=Mus musculus GN=Myh10 PE=1 SV=2	1.00	1.00	1.00	0.02	0	0
Q64285	Bile salt-activated lipase OS=Mus musculus GN=Cel PE=1 SV=1	1.00	0.00	0.02	0.00	0	6
Q91V41	Ras-related protein Rab-14 OS=Mus musculus GN=Rab14 PE=1 SV=3	1.00	0.02	1.00	1.00	0	1
Q99P72	Reticulon-4 OS=Mus musculus GN=Rtn4 PE=1 SV=2	1.00	0.02	1.00	0.00	0	1
Q9D0K2	Succinyl-CoA:3-ketoacid-coenzyme A transferase 1, mitochondrial OS=Mus musculus GN=Oxct1 PE=1 SV=1	1.00	0.02	1.00	1.00	0	1
Q9D1D4	Transmembrane emp24 domain-containing protein 10 OS=Mus musculus GN=Tmed10 PE=2 SV=1	1.00	0.00	1.00	0.00	0	2
Q9EPC2	Fibroblast growth factor 23 OS=Mus musculus GN=Fgf23 PE=1 SV=1	1.00	0.03	1.00	1.00	0	1
Q9JIW9	Ras-related protein Ral-B OS=Mus musculus GN=Ralb PE=2 SV=1	1.00	0.05	1.00	1.00	0	1

		Spectral Counts				Normalized Spectral Counts				
p2	p14	e15.5	e17.5	p2	p14	e15.5	e17.5	p2	p14	gene
1	7	0	14	6	12	0.0000	0.0020	0.0017	0.0033	Dpysl2
0	4	0	0	0	7	0.0000	0.0000	0.0000	0.0000	Anxa3
0	0	0	2	0	0	0.0000	0.0003	0.0000	0.0000	Bcat2
4	14	11	60	40	66	0.0085	0.0084	0.0114	0.0200	Anxa2
4	10	0	0	26	25	0.0000	0.0000	0.0074	0.0011	Anxa1
1	0	0	0	1	0	0.0000	0.0000	0.0003	0.0000	Glud1
0	0	2	2	0	0	0.0015	0.0003	0.0000	0.0000	Mif
1	2	0	0	2	9	0.0000	0.0000	0.0006	0.0000	Cav1
0	0	1	1	0	0	0.0008	0.0001	0.0000	0.0000	Hmga2
0	0	0	2	0	0	0.0000	0.0003	0.0000	0.0000	Erp29
0	0	3	5	0	0	0.0023	0.0007	0.0000	0.0000	Drd2
3	9	14	87	19	29	0.0108	0.0122	0.0054	0.0133	Ywhaz
1	2	0	0	4	5	0.0000	0.0000	0.0011	0.0000	Slc9a3r1
1	1	0	0	2	2	0.0000	0.0000	0.0006	0.0000	S100a13
1	2	0	0	2	3	0.0000	0.0000	0.0006	0.0000	Anpep
0	1	0	0	0	1	0.0000	0.0000	0.0000	0.0000	Xdh
0	0	0	2	0	0	0.0000	0.0003	0.0000	0.0000	Llgl2
0	0	0	2	0	0	0.0000	0.0003	0.0000	0.0000	Pde3b
0	0	0	6	0	0	0.0000	0.0008	0.0000	0.0000	Hadh
0	1	0	0	0	2	0.0000	0.0000	0.0000	0.0000	Myh10
1	4	0	25	3	9	0.0000	0.0035	0.0009	0.0011	Cel
0	0	0	2	0	0	0.0000	0.0003	0.0000	0.0000	Rab14
0	3	0	1	0	4	0.0000	0.0001	0.0000	0.0000	Rtn4
0	0	0	2	0	0	0.0000	0.0003	0.0000	0.0011	Oxct1
0	3	0	4	0	4	0.0000	0.0006	0.0000	0.0000	Tmed10
0	0	0	2	0	0	0.0000	0.0003	0.0000	0.0000	Fgf23
0	0	0	1	0	0	0.0000	0.0001	0.0000	0.0000	Ralb

protein	annotation	FDR e15.5	FDR e17.5	FDR p2	FDR p14	Unique Petides e15.5	Unique Petides e17.5	Unique Petides p2	Unique Petides p14	Spectral e15.5
A2ASQ1	Agrin OS=Mus musculus GN=Agrn PE=2 SV=1	0.07	1.00	1.00	1.00	1	0	0	0	1
O55026	Ectonucleoside triphosphate diphosphohydrolase 2 OS=Mus musculus GN=Entpd2 PE=1 SV=2	1.00	1.00	1.00	0.00	0	0	0	2	0
P07356	Annexin A2 OS=Mus musculus GN=Anxa2 PE=1 SV=2	0.00	0.00	0.00	0.00	2	6	4	14	11
P07724	Serum albumin OS=Mus musculus GN=Alb PE=1 SV=3	1.00	0.02	1.00	0.00	0	1	0	4	0
P08228	Superoxide dismutase [Cu-Zn] OS=Mus musculus GN=Sod1 PE=1 SV=2	0.02	0.00	0.00	0.00	1	4	3	4	6
P09055	Integrin beta-1 OS=Mus musculus GN=Itgb1 PE=1 SV=1	1.00	0.03	0.08	0.04	0	1	1	1	0
P14211	Calreticulin OS=Mus musculus GN=Calr PE=1 SV=1	0.00	0.00	0.00	0.00	2	7	5	9	6
P16045	Galectin-1 OS=Mus musculus GN=Lgals1 PE=1 SV=3	0.02	0.00	0.00	0.00	1	6	4	7	7
P21981	Protein-glutamine gamma-glutamyltransferase 2 OS=Mus musculus GN=Tgm2 PE=1 SV=4	1.00	1.00	1.00	0.00	0	0	0	6	0
Q04857	Collagen alpha-1(VI) chain OS=Mus musculus GN=Col6a1 PE=2 SV=1	1.00	1.00	0.02	0.04	0	0	1	1	0
Q3V1T4	Prolyl 3-hydroxylase 1 OS=Mus musculus GN=Lepre1 PE=2 SV=2	1.00	1.00	1.00	0.02	0	0	0	1	0
Q60997	Deleted in malignant brain tumors 1 protein OS=Mus musculus GN=Dmbt1 PE=1 SV=2	1.00	1.00	0.00	1.00	0	0	2	0	0
Q9D1D6	Collagen triple helix repeat-containing protein 1 OS=Mus musculus GN=Cthrc1 PE=2 SV=1	1.00	0.05	1.00	1.00	0	1	0	0	0
Q9D8U4	Complement C1q tumor necrosis factor-related protein 2 OS=Mus musculus GN=C1qtnf2 PE=2 SV=[illegible]	1.00	0.02	0.03	1.00	0	1	1	0	0
Q9Z1T2	Thrombospondin-4 OS=Mus musculus GN=Thbs4 PE=2 SV=1	1.00	0.05	1.00	1.00	0	1	0	0	0

l Counts			Normalized Spectral Counts				
e17.5	p2	p14	e15.5	e17.5	p2	p14	gene
0	0	0	0.0008	0.0000	0.0000	0.0000	Agrn
0	0	2	0.0000	0.0000	0.0000	0.0006	Entpd2
60	40	66	0.0085	0.0084	0.0114	0.0186	Anxa2
8	0	6	0.0000	0.0011	0.0000	0.0017	Alb
34	20	15	0.0046	0.0048	0.0057	0.0042	Sod1
2	1	1	0.0000	0.0003	0.0003	0.0003	Itgb1
53	21	22	0.0046	0.0075	0.0060	0.0062	Calr
37	23	40	0.0054	0.0052	0.0065	0.0113	Lgals1
0	0	12	0.0000	0.0000	0.0000	0.0034	Tgm2
0	2	1	0.0000	0.0000	0.0006	0.0003	Col6a1
0	0	1	0.0000	0.0000	0.0000	0.0003	Lepre1
0	3	0	0.0000	0.0000	0.0009	0.0000	Dmbt1
2	0	0	0.0000	0.0003	0.0000	0.0000	Cthrc1
4	2	0	0.0000	0.0006	0.0006	0.0000	C1qtnf2
2	0	0	0.0000	0.0003	0.0000	0.0000	Thbs4

protein	annotation	FDR e15.5	e17.5	p2	p14	Unique Petides e15.5	e17.5	p2
P49817	Caveolin-1 OS=Mus musculus GN=Cav1 PE=1 SV=1	1.00	1.00	0.05	0.00	0	0	1
P60766	Cell division control protein 42 homolog OS=Mus musculus GN=Cdc42 PE=1 SV=2	1.00	0.00	0.02	1.00	0	2	1
P61168	D(2) dopamine receptor OS=Mus musculus GN=Drd2 PE=2 SV=2	0.04	0.03	1.00	1.00	1	1	0
P67871	Casein kinase II subunit beta OS=Mus musculus GN=Csnk2b PE=1 SV=1	1.00	1.00	0.06	1.00	0	0	1
P68040	Guanine nucleotide-binding protein subunit beta-2-like 1 OS=Mus musculus GN=Gnb2l1 PE=1 SV=3	1.00	1.00	0.02	0.05	0	0	1
P70441	Na(+)/H(+) exchange regulatory cofactor NHE-RF1 OS=Mus musculus GN=Slc9a3r1 PE=1 SV=3	1.00	1.00	0.02	0.00	0	0	1
Q3U1J4	DNA damage-binding protein 1 OS=Mus musculus GN=Ddb1 PE=1 SV=2	1.00	1.00	0.08	1.00	0	0	1
Q91X79	Elastase 1, pancreatic OS=Mus musculus GN=Cela1 PE=2 SV=1	1.00	0.00	0.02	0.00	0	4	1

	Spectral Counts				Normalized Spectral Counts				
p14	e15.5	e17.5	p2	p14	e15.5	e17.5	p2	p14	gene
2	0	0	2	9	0	0	0.00057	0.00253	Cav1
0	0	3	3	0	0	0.00042	0.00085	0	Cdc42
0	3	5	0	0	0.00231	0.0007	0	0	Drd2
0	0	0	2	0	0	0	0.00057	0	Csnk2b
1	0	0	3	2	0	0	0.00085	0.00056	Gnb2l1
2	0	0	4	5	0	0	0.00114	0.00141	Slc9a3r1
0	0	0	1	0	0	0	0.00028	0	Ddb1
4	0	28	4	11	0	0.00394	0.00114	0.0031	Cela1

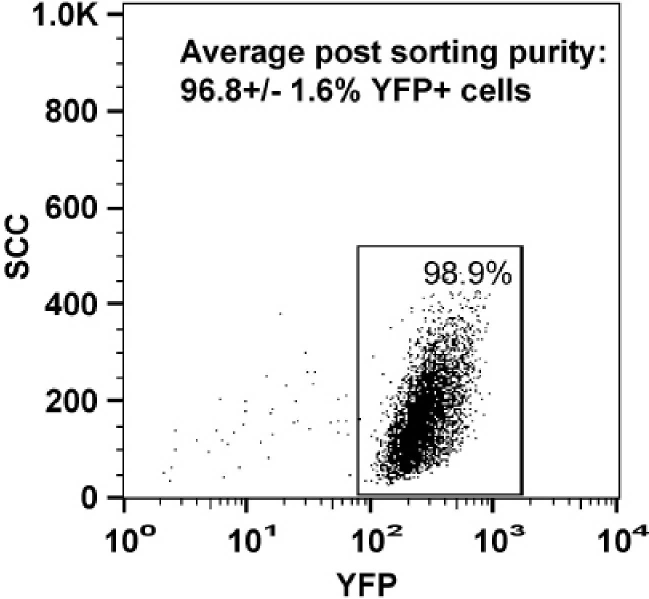

Supplementary Figure 1, Russ et al.

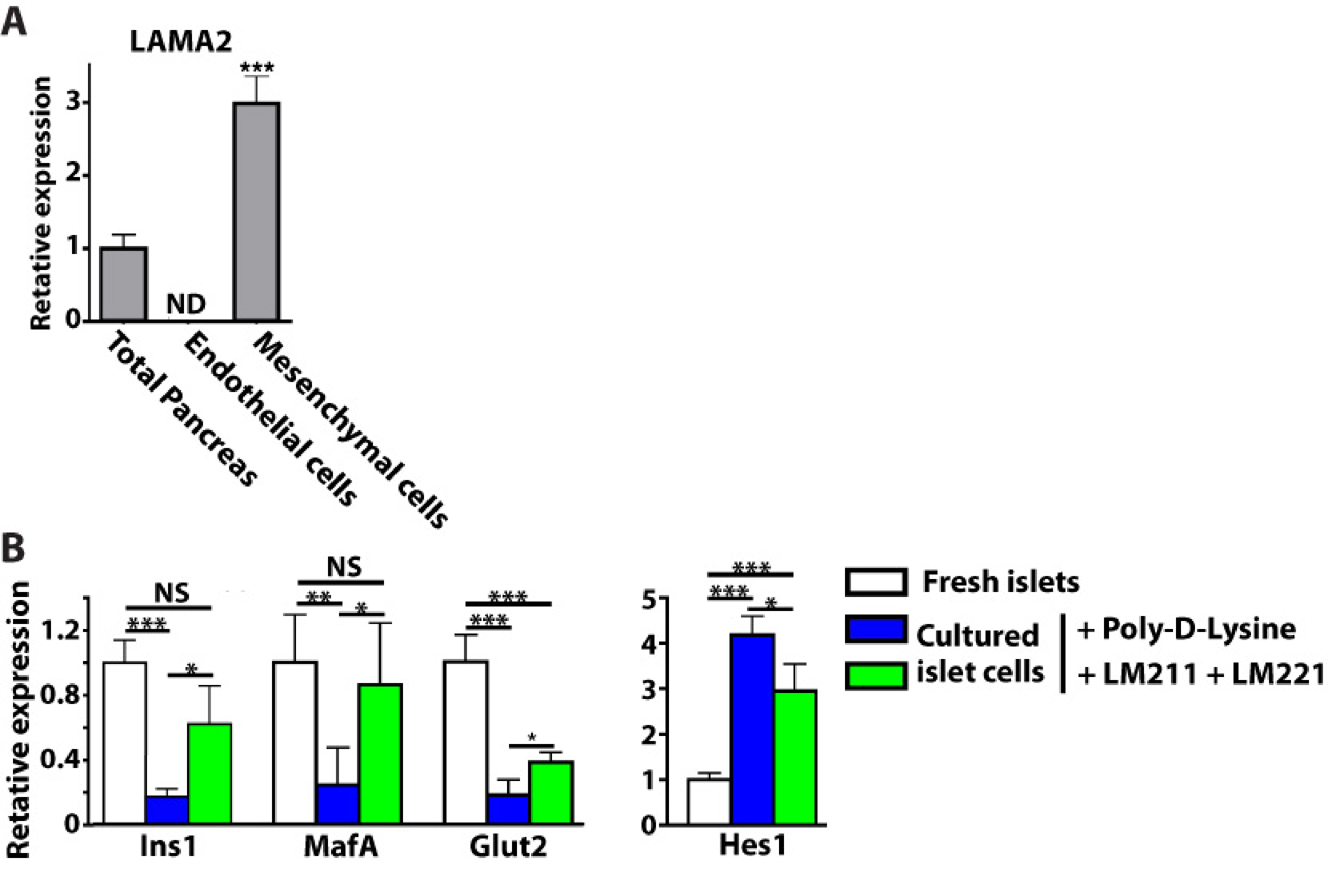

Supplementary Figure 2, Russ et al.

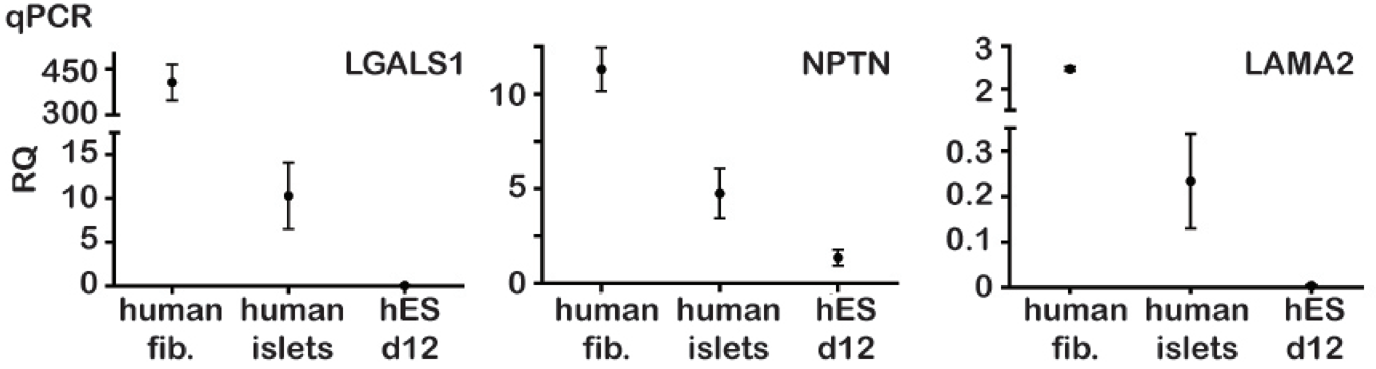

Supplementary Figure 3, Russ et al.
